# Population health and implementation outcomes of self-testing for SARS-CoV-2 using antigen detecting diagnostics: a systematic review and meta-analysis

**DOI:** 10.1016/j.eclinm.2026.103838

**Published:** 2026-04-07

**Authors:** Lukas E. Brümmer, Verena Faehling, Sean McGrath, Ana-Mihaela Zorger, Karina Worbes, Christian Erdmann, Hannah Tolle, Stephan Katzenschlager, Seda Yerlikaya, Maurizio Grilli, Nira R. Pollock, Berra Erkosar, Aurelien Mace, Stefano Ongarello, Cheryl C. Johnson, Jilian A. Sacks, Jane Cunningham, Nicole Skoetz, Claudia M. Denkinger, Rose A. Lee

**Affiliations:** a Department of Infectious Disease and Tropical Medicine, Center for Infectious Diseases, Heidelberg University Hospital, Heidelberg, Germany; b German Center for Infection Research (DZIF), partner site Heidelberg University Hospital, Heidelberg, Germany; c London School of Hygiene and Tropical Medicine, London, United Kingdom; d Department of Biostatistics, Yale School of Public Health, New Haven, CT, United States of America; e Department of Population Medicine, Harvard Medical School and Harvard Pilgrim Health Care Institute, Boston, MA, United States of America; f Institute for Public Health, Faculty of Medicine and University Hospital Cologne, University of Cologne, Cologne, Germany; g FH Muenster University of Applied Sciences, Muenster, Germany; h Department of Anesthesiology, Faculty of Medicine, Heidelberg University, Heidelberg, Germany; i Library, University Medical Center Mannheim, Mannheim, Germany; j Department of Laboratory Medicine, Boston Children's Hospital, Boston, MA, United States of America; k FIND, the global alliance for diagnostics, Geneva, Switzerland; l International Federation of Red Cross and Red Crescent Societies (IFRC), Geneva, Switzerland; m Global HIV, Hepatitis and STIs Programmes, World Health Organization, Geneva, Switzerland; n Health Emergencies Preparedness and Response Programme, World Health Organization, Geneva, Switzerland; o Microbiology Service, Department of Laboratory Medicine, Warren G. Magnuson Clinical Center, National Institutes of Health, Bethesda, MD, United States of America

**Keywords:** COVID-19 self-testing, Antigen-based testing, Systematic review, Meta-analysis, Population health and implementation outcomes

## Abstract

**Background:**

COVID-19 antigen-based rapid diagnostic tests (Ag-RDTs) for self-testing (C19ST) have been widely implemented. However, evidence on population health and implementation outcomes remains limited. We systematically evaluated population health and implementation outcomes of C19ST to inform WHO guidelines and pandemic preparedness.

**Methods:**

We conducted a systematic review and meta-analysis (PROSPERO CRD42022299977), searching Embase, MEDLINE, Web of Science, MedRxiv, clinicaltrials.gov and the Cochrane Library from Dec 1, 2020, to Oct 1, 2025. We included cohort, case–control, cross-sectional, before-and-after, and randomised studies with symptomatic and asymptomatic participants using commercially available C19ST Ag-RDTs. Primary outcomes included C19ST population health (case detection, test positivity, number needed to test (NNT)) and implementation outcomes (uptake, adherence, result reporting). Meta-analyses used binomial-normal generalised linear mixed models; study quality assessment used the JBI Quasi-Experimental Tool.

**Findings:**

Of 19,473 records screened, 61 studies (87 datasets) with 25,288,225 participants (78% asymptomatic) were included. C19ST detected 31 (95% CI 14–65) cases per 1000 individuals, missing 14% (95% CI 1–65%) compared to molecular testing. Test positivity was 7 per 1000 tests (95% CI 3–15); false positives occurred in 0.4% (95% CI 0.2–1.0%). NNT was 75 in symptomatic and 1002 in asymptomatic individuals. Uptake, adherence, and result reporting were high, but estimates were limited by selection bias. C19ST was also reported to improve perceptions of safety, and reduce self-isolation, workplace absenteeism, and other societal disruptions, supporting the continuity of daily activities. Heterogeneity was substantial.

**Interpretation:**

C19ST improves case detection and supports pandemic control with acceptable accuracy and meaningful societal benefits. These findings support the use of antigen-based self-testing as a complementary tool for a pandemic response. Studies included further highlight the limited use of standardised frameworks for evaluating population health and implementation outcomes of novel diagnostics.

**Funding:**

Ministry of Science, Research and Arts of the State of Baden-Wuerttemberg, Germany.

**Research in context**

**Evidence before this study**

We systematically reviewed the literature through October 1, 2025 on population health and implementation outcomes of COVID-19 self-testing (C19ST). Prior systematic reviews emphasised C19ST diagnostic accuracy, reporting pooled sensitivities ∼70–91% and ≥99% specificity in comparison to reverse transcriptase polymerase chain reaction (RT-PCR), and examined technical aspects such as performance by sample type, use of digital interventions, and supervision strategies. Although data on feasibility, acceptability, and epidemiological outcomes have been reported, these analyses drew from a small number of studies and analysed relevant data only narratively. As a result, important gaps remain in understanding how self-testing can be effectively implemented at scale, its performance across diverse settings, and what broader societal and public health impacts might result.

**Added value of this study**

This study offers the most comprehensive synthesis to date of population health and implementation outcomes of C19ST. Across 61 studies (87 datasets) encompassing over 25 million participants, we meta-analysed population health outcomes such as case detection and number needed to test, along with implementation metrics including uptake, adherence, and result reporting. We also synthesised the evidence on potential social harms and broader societal impacts, including improvements in societal functioning such as return to work and reduced duration of self-isolation. The evidence synthesis informed a WHO guidance on the optimised use of C19ST as a public health tool.

**Implications of all the available evidence**

C19ST can enhance case detection and pandemic control as a complement to professional testing and RT-PCR. When implemented programmatically, C19ST achieved case detection rates similar to those reported for RT-PCR in previous systematic reviews, likely due to serial testing and easier access, despite lower sensitivity in head-to-head comparisons. Societal benefits include reduced disruption, greater perceptions of safety, and increased continuity of daily activities. However, even under widespread C19ST-implementation, confirmatory testing and integration with other pandemic control measures remain essential. To optimise implementation of self-testing in the response to future public health emergencies, standardised evaluation frameworks should be applied to assess added utility, cost-effectiveness, and equity of novel diagnostics and testing strategies such as self-testing. Our findings support the use of antigen-based rapid diagnostic self-testing in pandemic preparedness and its integration into structured public health programs.

## Introduction

Antigen-based rapid diagnostic tests (Ag-RDTs) using lateral flow immunoassays (LFIAs) have been widely adopted for COVID-19 self-testing (C19ST) to detect SARS-CoV-2 infection.^1^ Multiple systematic reviews have established the diagnostic accuracy of C19ST compared with RT-PCR, reporting pooled sensitivities of ∼70–91% dependent on underlying factors such as symptom status or sample type, with consistently high specificities ≥99%.^2^, ^3^, ^4^, ^5^ Beyond diagnostic accuracy, evidence on the broader population-level effects of C19ST remains limited. One prior systematic review also addressed C19ST test positivity, feasibility, acceptability, and selected impact outcomes, such as fewer school closures or reduced quarantine.^2^ However, these findings were largely synthesised narratively with reported ranges across heterogeneous studies, reflecting the more limited evidence base available at the time. As a result, gaps persist regarding how C19ST performs at scale, including its contribution to case detection, missed infections, false positives, test efficiency, and implementation performance, as well as its downstream effects on transmission, morbidity, and broader societal outcomes.

In 2022, the World Health Organization (WHO) issued guidance on C19ST implementation,^1^ informed in part by a systematic review conducted by members of the present author team.^6^ At that time, the evidence base for population health and implementation outcomes was limited, precluding robust quantitative synthesis. Since then, the evidence base has expanded considerably, enabling a more comprehensive and methodologically rigorous assessment of these outcomes.

In this updated systematic review and meta-analysis, we synthesise population-level health and implementation outcomes using all available evidence through October 1st, 2025. We quantitatively meta-analyse key population-level C19ST performance outcomes including case detection, number needed to test, missed-case, false-positive, and invalid test proportions, as well as implementation outcomes such as test uptake, adherence, and result reporting. We further narratively synthesise evidence on transmission impact, morbidity, linkage to care, behavioural responses, and broader societal effects. Together, these analyses provide a comprehensive and policy-relevant evaluation of the population-level effects of C19ST to support modelling efforts and guide self-testing implementation for future outbreaks and pandemics.

## Methods

A study protocol (Supplement Text S1) was developed according to standard guidelines for systematic reviews,^7^^,^^8^ reviewed by an independent WHO guidelines methodologist (EA), and registered on PROSPERO (registration number: CRD42022299977). This review was designed in collaboration with the WHO and WHO Guideline Development Group.^1^^,^^6^ Methods from our initial analysis^6^^,^^9^ were updated following best practice guidelines for updating systematic reviews.^10^ We completed the PRISMA checklist.^11^

### Search strategy and selection criteria

This was a systematic review and meta-analysis of implementation studies evaluating C19ST. We included studies implementing C19ST with a commercially available point-of-care Ag-RDT in symptomatic or asymptomatic individuals during the COVID-19 pandemic, in settings without prior widespread use (Supplement Text S2). Studies were required to report at least one of the outcomes designated by the WHO Guideline Development Group (Table 1)^1^ and include ≥100 human participants to improve model fitting and estimate precision. The majority of the study duration had to occur between March 11, 2020 and May 5, 2023, the period during which COVID-19 was classified as a Public Health Emergency of International Concern by WHO. We considered retrospective or prospective cohort or nested cohort studies, case–control or cross-sectional studies, before and after studies, and randomised studies. We excluded studies limited to individuals with confirmed SARS-CoV-2 infection, ease-of-use studies, and diagnostic accuracy studies reporting only on test sensitivity and specificity.

**Table 1:** Prioritised outcomes and sub-outcomes as ranked by the members of the WHO guideline development group.

Prioritised outcomes	Prioritised sub-outcomes	Synthesised related outcomes & definitions	Score and rank
Population health outcomes	Epidemiologic impact	**Case Detection**: Proportion of individuals testing positive on C19ST among all individuals who performed C19ST. **Missed cases proportion:** RT-PCR–positive individuals who tested negative by C19ST among all RT-PCR positive cases (only considering studies where both tests were performed on all participants) **Test Positivity**: Proportion of positive C19ST results out of all C19STs performed, including repeat tests in serial testing protocols. • Number Needed to Test (NNT): Reciprocal of the pooled estimate of the proportion of positive test results (NNT = 1/test positivity); number of C19STs required to identify one positive result. **False positive proportion:** Proportion of false positive C19ST results among paired tests with a negative RT-PCR result (only considering studies where all C19ST were confirmed by RT-PCR) **Invalid test proportion:** Proportion of invalid or uninterpretable C19ST results due to faint bands, missing control lines, or other errors	6: Important
	Impact on virus transmission	Narrative synthesis of data on viral transmission (e.g., changes in incidence, prevalence, cases averted, secondary contacts, secondary attack proportion)	6: Important
	Impact on morbidity and/or mortality	Narrative synthesis of changes in patient morbidity (hospitalisation, disease severity) and/or mortality (deaths averted) attributed to C19ST use	6: Important
Implementation outcomes	Linkage for positive tests/actions after positive test result	Narrative synthesis of actions following a C19ST-positive result (e.g., confirmatory testing, isolation).	7: Critical
	Test uptake	**Test Uptake:** Proportion of individuals who accepted or performed C19STs out of all those offered testing.	6.5: Critical
	Time to diagnosis	Narrative synthesis of changes in time from performing C19ST to final diagnosis of SARS-CoV-2 status or clinical decision based on test result.	6.5: Critical
	Testing frequency	Narrative synthesis of test frequency among studies. **Test Schedule Adherence:** Proportion of tests completed according to the assigned testing frequency (e.g., once weekly, twice weekly) out of all required tests.	6: Important
	Result reporting	**Result Reporting:** Proportion of C19ST results reported by participants to study investigators, health authorities, or digital platforms.	6: Important
	Behaviour change	Narrative synthesis of any reported changes in participant behaviour due to C19ST use (e.g., mask-wearing, social distancing).	5.5: Important
	Linkage for negatives/actions after negative test result	Narrative synthesis of actions of participants with negative C19ST results (e.g., return to work, exemption from self-isolation).	5: Important
Social harm		Narrative synthesis of any reported adverse social impacts of C19ST use (e.g., stigma, anxiety, discrimination).	7: Critical
Broader societal effects		Narrative synthesis of impacts of C19ST use on absenteeism (work, school), schooling continuity, resource utilisation, user costs (including opportunity costs), and time savings.	6: Important

Quantitatively meta-analysed outcomes are marked in bold (also see Table 3). Outcomes measured per individual (case-based denominators) include case detection, missed-case proportion, and test uptake. Outcomes measured per test (test-based denominators) include test positivity (and number needed to test), false-positive proportion, invalid-test proportion, test-schedule adherence, and result reporting.

C19ST = COVID-19 self-testing; NNT = number needed to test; RT-PCR = reverse transcriptase polymerase chain reaction.

An information specialist (MG) searched Embase, MEDLINE (via PubMed), Web of Science, medRxiv, clinicaltrials.gov and the Cochrane Library for articles using terms including “COVID-19”, “SARS-CoV-2”, “antigen test”, “self-testing”, and “home testing”, with no language or geographic restrictions, for studies published between December 1st, 2020 (start of C19ST availability) and October 1st, 2025. The full search strategy is provided in Supplement Text S3. In addition, references of included studies were screened manually by two reviewers (LEB and either HT/VF) and for any study protocol included in the full text review, published studies related to this protocol were also searched for by one reviewer (LEB) using Google.^12^ We did not seek unpublished data beyond contacting study authors for clarification where needed.

### Study selection and data extraction

Two authors (LEB and either AMZ/VF) independently screened all titles and abstracts followed by full-text review (LEB and either AMZ/KW/VF), using Rayyan^13^ for literature organisation and DeepL Translator^14^ and ChatGPT^15^ to translate non-English or non-German articles. Disagreements were resolved by discussion or senior adjudication (RL). Two reviewers (LEB and either AMZ/KW/VF) extracted study-level summary data independently using a standardised form; discrepancies were resolved by discussion or senior adjudication (RL). Study authors were contacted for clarification if needed. When intra-study results were stratified (e.g., Ag-RDT manufacturer or setting), distinct, mutually exclusive datasets were extracted.

### Quality assessment

Study quality was assessed using the revised Joanna Briggs Institute (JBI) Critical Appraisal Tool for Quasi-Experimental Studies^16^^,^^17^ with three customised interpretation guides developed for specific outcome domains (Supplement Text S4). Each interpretation guide criterion was scored as one (“yes”) or zero (“unclear” or “no”). Studies scoring <60% of total points were rated low quality; 60–80% moderate quality; >80% high quality. If multiple data sets were extracted from a study, each was assessed separately. Two reviewers (LEB and either HT/AK/RL/VF) independently assessed quality, resolving disagreements through discussion.

### Data analysis

For outcomes with four or more datasets, we prepared forest plots and conducted meta-analyses and subgroup analyses using binomial-normal generalised linear mixed models (GLMM), reporting point estimates and 95% confidence intervals (Supplement Text S5). GLMMs enable synthesis of near-zero proportions and zero-event data while accounting for within-study uncertainties.^18^ When multiple data sets were extracted from a study, they were treated independently in all analyses. Eight outcomes defined in Table 1 were meta-analysed to address the WHO Guideline Development Group objectives. For outcomes with less than four data sets or substantial methodological heterogeneity, we conducted narrative summaries. Heterogeneity was measured by the inconsistency index (I^2^), which compares the between-study variance to within-study variance. Given the large sample size of the studies included, we expected I^2^ to be very high for most outcomes (because the within-study variance is typically very small), and report prediction intervals as absolute measures of heterogeneity as well.^19^ Publication bias was assessed by visually inspecting funnel plots.^20^ Further details on data analysis including predefined subgroups and cost adjustments are detailed in Supplement Text S6.^21^^,^^22^ Analyses were performed in Python (3.9.13) and R (version 4.1.1) using the “metafor” package.^23^

### Ethics statement

Ethics approval was not required for this study.

### Role of the funding source

The study was funded through the Ministry of Science, Research and Arts of the State of Baden-Wuerttemberg, Germany. Authors also contributed time out of other third-party funded roles, which are outlined in the acknowledgement section. The funders of the study had no role in study design, data collection, data analysis, data interpretation, or writing of the report.

## Results

### Study description

**Fig. 1:**
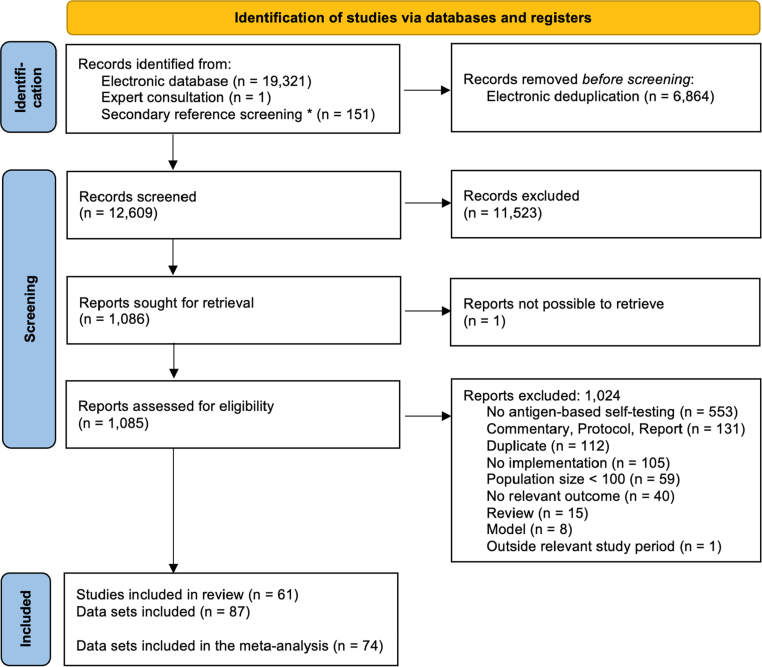
**PRISMA flow diagram.** The PRISMA flow diagram for systematic review based on Page et al.,^11^ ∗ Studies identified through secondary reference screening include any studies related to study protocols found via clinicaltrials.gov and the Cochrane database for clinical trials. n = number of studies/data sets.

The search yielded 19,473 records, with 61 studies eligible for inclusion^24^, ^25^, ^26^, ^27^, ^28^, ^29^, ^30^, ^31^, ^32^, ^33^, ^34^, ^35^, ^36^, ^37^, ^38^, ^39^, ^40^, ^41^, ^42^, ^43^, ^44^, ^45^, ^46^, ^47^, ^48^, ^49^, ^50^, ^51^, ^52^, ^53^, ^54^, ^55^, ^56^, ^57^, ^58^, ^59^, ^60^, ^61^, ^62^, ^63^, ^64^, ^65^, ^66^, ^67^, ^68^, ^69^, ^70^, ^71^, ^72^, ^73^, ^74^, ^75^, ^76^, ^77^, ^78^, ^79^, ^80^, ^81^, ^82^, ^83^, ^84^ (excluded studies in Supplement Table S1; PRISMA diagram, Fig. 1). The included studies contributed 87 datasets across diverse global settings. Study characteristics, including geographic region, target population, testing frequency, and number of participants are summarised in Table 2.

**Table 2:** Characteristics of 87 data sets from 61 studies included in the systematic review.

Author [reference]	Data set ID	Time frame (Month/Year)	Location	Target population	Number of study participants	Frequency of testing	Number of C19STs reported
Agusti, C., et al.^24^	24_1	12/21–2/22	Spain	Healthcare Professionals	1400	Single	111
Agusti, C., et al.^24^	24_2	12/21–2/22	Spain	K-12 students and staff	700	Single	192
Bresser, M., et al.^25^	25_0	2/22–4/22	Zambia	General population	1123	Single	1121
Chan, H.K., et al.^26^	26_0	11/22–2/23	Malaysia	Manufacturing employees	2959	Unclear	Unclear
Chen, S.H., et al.^27^	27_1	5/22–6/22	Taiwan	General population	426	Single	Unclear
Chen, S.H., et al.^27^	27_2	5/22–6/22	Taiwan	General population	201	Single	Unclear
Chong, S.J., et al.^28^	28_1	1/22–2/22	Singapore	General population	342,302	Routine	Unclear
Chong, S.J., et al.^28^	28_2	1/22–2/22	Singapore	General population	297,340	Routine	Unclear
Chong, S.J., et al.^28^	28_3	1/22–2/22	Singapore	General population	73,259	Routine	Unclear
Chong, S.J., et al.^28^	28_4	1/22–2/22	Singapore	General population	564	Routine	Unclear
D'Agostino, E.M., et al.^29^	29_0	11/21–12/21	United States	General population	2469	Unclear	2469
Downs, L. O., et al.^30^	30_0	11/20–1/21	United Kingdom	Healthcare Professionals	8657	Routine	46,503
Du, Z., et al.^31^	31_0	2/22–3/22	Hong Kong	General population	Unclear	Unclear	Unclear
Engels, G., et al.^32^	32_1	5/21–7/21	Germany	K-12 students and staff	836	Routine	2896
Engels, G., et al.^32^	32_2	5/21–7/21	Germany	K-12 students and staff	190	Routine	1022
Gorgels, K.M.F., et al.^33^	33_0	5/21–4/22	Netherlands	General population	3,599,894	Unclear	Unclear
Harmon, A., et al.^34^	34_0	Unclear	United States	General population	257	Routine	2951
Herbert, C., et al. (a)^35^	35_1	8/21–2/22	United States	General population	1083	Routine	6580
Herbert, C., et al. (a)^35^	35_2	8/21–2/22	United States	General population	801	Routine	Unclear
Herbert, C., et al. (b)^36^	36_1	4/21–7/21	United States	General population	1366	Unclear	1900
Herbert, C., et al. (b)^36^	36_2	9/21–11/21	United States	General population	2012	Unclear	3742
Herbert, C., et al. (b)^36^	36_3	9/21–9/21	United States	General population	5007	Unclear	11,154
Herbert, C., et al. (b)^36^	36_4	10/21–11/21	United States	General population	1856	Unclear	5199
Herbert, C., et al. (b)^36^	36_5	10/21–11/21	United States	General population	954	Unclear	2626
Hirst, J. A., et al.^37^	37_1	10/20–1/21	United Kingdom	University students and staff	2132	Routine	2731
Hirst, J. A., et al.^37^	37_2	10/20–1/21	United Kingdom	University students and staff	183	Routine	459
Hoehl, S., et al.^38^	38_0	Unclear	Germany	K-12 students and staff	711	Routine	11,385
Hogg, C. et al.^39^	39_0	5/21–8/21	United Kingdom	Healthcare Professionals	Unclear	Routine	115,593
Hu, Y. et al.^40^	40_0	11/22–11/22	China	Unclear	17,655	Routine	Unclear
Iftner, T., et al.^41^	41_1	5/21–7/21	Germany	Healthcare Professionals	1015	Single	468
Iftner, T., et al.^41^	41_2	5/21–7/21	Germany	Healthcare Professionals		Single	479
Iftner, T., et al.^41^	41_3	5/21–7/21	Germany	Healthcare Professionals		Single	464
Iftner, T., et al.^41^	41_4	5/21–7/21	Germany	Healthcare Professionals		Single	481
Kheiroddin, P., et al.^42^	42_0	4/21–5/21	Germany	K-12 students and staff	Unclear	Routine	Unclear
Kiene, S.M. et al.^43^	43_0	11/21–3/22	United States	K-12 students and staff	761	Routine	Unclear
Kim, A. E. et al.^44^	44_0	2/22–12/22	United States	University students and staff	5575	Unclear	17,572
Koirala, A. et al.^45^	45_0	11/21–12/21	Australia	K-12 students and staff	9887	Routine	35,438
Kwan, T. H. et al.^46^	46_0	4/22–4/22	Hong Kong	General population	21,769	Routine	Unclear
Lamb, G., et al.^47^	47_0	11/20–1/21	United Kingdom	Healthcare Professionals	6702	Routine	45,022
Lau, C.S., et al.^48^	48_0	5/21–3/22	Singapore	General population	161	Routine	Unclear
Love, N.K., et al. (a)^49^	49_0	4/21–7/21	United Kingdom	General population	26,123	Routine	124,010
Love, N.K., et al. (b)^50^	50_0	12/20–1/21	United Kingdom	General population	1760	Routine	2505
Marbán-Castro, E. et al.^51^	51_1	6/22–12/22	Georgia	Unclear	2180	Routine	51,912
Marbán-Castro, E. et al.^51^	51_2	6/22–12/22	Georgia	General population	582	Routine	1073
McDaniels-Davidson, C. et al.^52^	52_0	10/21–1/22	United States	K-12 students and staff	159	Routine	616
Moonan, P.K. et al.^53^	53_0	1/21–3/22	United States	General population	15,923	Unclear	Unclear
Nagasawa M. et al.^54^	54_0	9/22–10/23	Japan	Healthcare Professionals	1481	Routine	Unclear
Nakgul, L. et al.^55^	55_0	12/21–3/22	Thailand	K-12 students and staff	164	Routine	1095
Nodora, J.N. et al.^56^	56_0	6/22–4/23	United States	General population	2189	Unclear	676
Papenburg, J., et al.^57^	57_0	7/21–10/21	Canada	Unclear	647	Routine	451
Pudasaini, S. et al.^58^	58_0	8/21–8/21	Germany	K-12 students and staff	130	Routine	Unclear
Ryan, F. et al.^59^	59_0	12/20–4/21	United Kingdom	Healthcare Professionals	189,364	Routine	1,094,413
Smit, T. et al.^60^	60_0	10/22–5/23	Netherlands	General population	17,030	Routine	Unclear
Soni, A. et al. (a)^61^	61_0	6/21–8/21	United States	General population	Unclear	Routine	Unclear
Soni, A. et al. (b)^62^	62_0	10/21–2/22	United States	General population	7303	Routine	Unclear
Stemler, J. et al.^63^	63_0	6/22–7/22	Germany	General population	2400	Single	419
Stohr, J.J.J.M., et al.^64^	64_1	12/20–1/21	Netherlands	General population	1758	Single	1595
Stohr, J.J.J.M., et al.^64^	64_2	12/20–1/21	Netherlands	General population	1771	Single	1606
Tinker, S.C., et al.^65^	65_0	2/21–4/21	United States	University students and staff	1347	Routine	9971
Tsang, N.N.Y., et al.^66^	66_0	3/22–4/22	Hong Kong	General population	8636	Routine	Unclear
Tsao, J. et al.^67^	67_0	1/22–1/22	United States	University students and staff	723	Single	723
Tulloch, J. S. P., et al.^68^	68_0	12/20–1/21	United Kingdom	Healthcare Professionals	498	Routine	1638
UK Health Security Agency^69^	69_0	1/21–2/21	United Kingdom	Healthcare Professionals	138	Routine	719
U.o.Liverpool^70^	70_1	12/20–7/21	United Kingdom	General population	321,884	Unclear	1,900,302
U.o.Liverpool^70^	70_2	2/21–8/21	United Kingdom	Healthcare Professionals	Unclear	Unclear	Unclear
Wachinger, J., et al.^71^	71_1	3/21–5/21	Germany	K-12 students and staff	34	Routine	Unclear
Wachinger, J., et al.^71^	71_2	3/21–5/21	Germany	K-12 students and staff	186	Routine	Unclear
Willeit, P., et al.^72^	72_1	3/21–3/21	Austria	K-12 students and staff	360,948	Routine	2,133,203
Willeit, P., et al.^72^	72_2	3/21–3/21	Austria	K-12 students and staff	319,672	Routine	944,631
Willeit, P., et al.^72^	72_3	3/21–3/21	Austria	K-12 students and staff	104,087	Routine	Unclear
Wong, S.C. et al.^73^	73_0	2/22–5/22	Hong Kong	Healthcare Professionals	6452	Routine	Unclear
Wu, S., et al.^74^	74_0	7/21–9/21	Singapore	Healthcare Professionals	8000	Routine	156,000
Yun, G. et al.^75^	75_1	3/22–5/22	South Korea	K-12 students and staff	1,039,087	Routine	3,017,696
Yun, G. et al.^75^	75_2	3/22–5/22	South Korea	K-12 students and staff	16,139,251	Routine	43,263,650
Yun, G. et al.^75^	75_3	3/22–5/22	South Korea	K-12 students and staff	87,387	Routine	206,799
Yun, G. et al.^75^	75_4	3/22–5/22	South Korea	K-12 students and staff	2,192,850	Routine	4,187,241
Davies, M. et al.^76^	76_0	6/21–8/21	United Kingdom	Unclear	105	Routine	280
Del Fiol, G. et al.^77^	77_0	12/22–8/23	United States	General population	2117	Unclear	Unclear
Mwangoka, G. W. et al.^78^	78_0	7/22–10/22	Tanzania	General population	517	Single	Unclear
Nacov, J. A. et al.^79^	79_1	12/22–5/23	Germany	General population	1993	Single	1119
Nacov, J. A. et al.^79^	79_2	6/23–6/23	Germany	General population		Single	310
Qasmieh, S. A. et al. (a)^80^	80_0	3/22–10/23	United States	General population	1918	Unclear	Unclear
Stirrup, O. et al.^81^	81_1	1/23–8/23	United Kingdom	Healthcare professional	Unclear	Routine	Unclear
Stirrup, O. et al.^81^	81_2	1/23–8/23	United Kingdom	Healthcare professional	Unclear	Routine	Unclear
Van Hagen, C. C. E. et al.^82^	82_0	3/22–5/23	Netherlands	General population	3152	Unclear	Unclear
Qasmieh, S. A. et al. (b)^83^	83_0	4/22–5/22	United States	General population	1030	Unclear	Unclear
Qasmieh, S. A. et al. (c)^84^	84_0	6/22–7/22	United States	General population	3042	Unclear	Unclear

Most studies were conducted in Europe (n = 27 studies), followed by North America (n = 18), Asia (n = 13), Africa (n = 2) and Australia (n = 1). C19ST was evaluated in the general population (n = 41 data sets), students and staff in K-12 and university settings (n = 24), healthcare professionals (n = 17), and manufacturing employees (n = 1). The target population was unclear in four data sets. Serial routine interval testing was reported in the majority of data sets (n = 52), while in 16 further data sets, testing was limited to a single instance and in the remaining 19 the testing frequency was unclear. Across all data sets, 25,288,225 study participants were included: 19,760,579 (78.1%) asymptomatic, 4,333,132 (17.1%) symptomatic, and 1,194,514 (4.7%) with unclear symptom status. All but 13 data sets^27^^,^^31^^,^^33^^,^^35^^,^^42^^,^^53^^,^^54^^,^^61^^,^^70^^,^^73^^,^^81^ contributed to at least one meta-analysed outcome. All eight meta-analysed outcomes demonstrated pronounced heterogeneity (I^2^ values ≥ 97%). The remaining outcomes were synthesised narratively.

### Quality assessment

The 74 data sets included in the meta-analysis achieved 58% of all possible quality points. Overall, 47 data sets were rated low quality (<60%) and 27 moderate (60–80%). Quality was highest for outcomes requiring confirmatory testing (missed cases and false-positive proportion), where all data sets were rated moderate using the confirmatory testing interpretation guide. In contrast, the majority of datasets contributing to C19ST participation or positivity were rated low quality when assessed using the corresponding guide (Supplement Figure S1).

### Publication bias

For several of the meta-analysed outcomes (missed cases proportion, false positives, invalid test proportion, test uptake, and test schedule adherence), we found an apparent relationship between the study size and the outcome estimate. We attribute this pattern to systematic differences associated with study size rather than to selective publication of positive or negative results (further details in Supplement Text S7).^85^

### Prioritised outcomes per WHO guideline development group

**Fig. 2:**
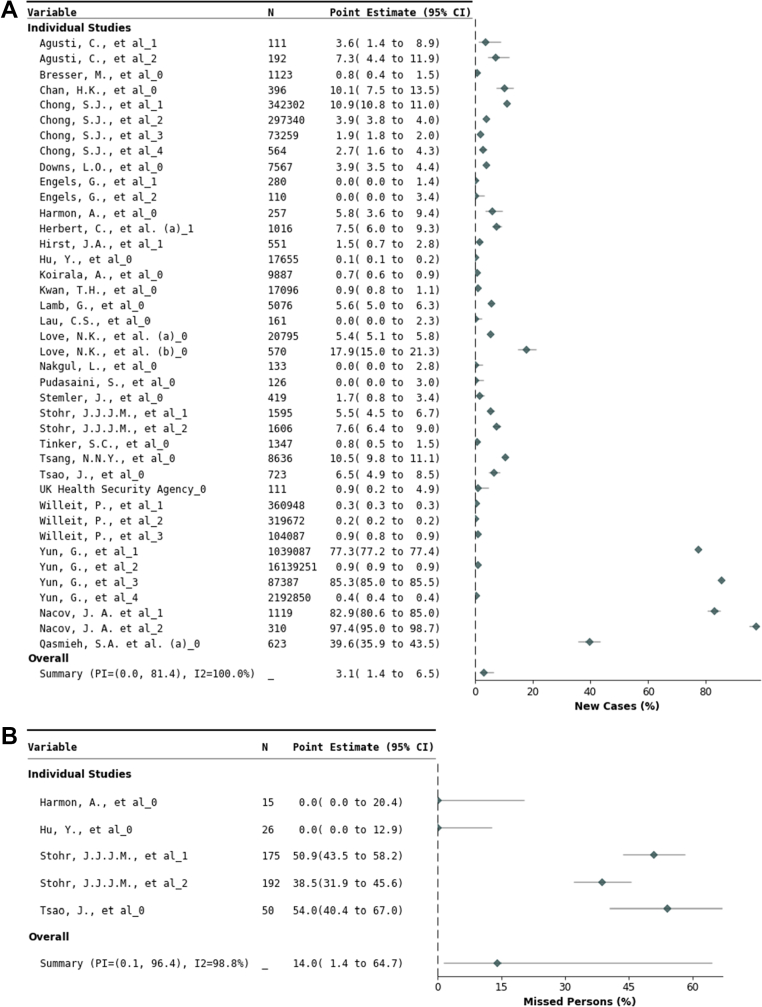
**Forest plots of the case detection and missed individuals.** Forest plots of A) the case detection proportion demonstrating COVID-19 cases identified by self-testing (C19ST), B) missed cases: the proportion of individuals who were SARS-CoV-2 positive by RT-PCR but received negative results only when conducting C19ST. CI = Confidence interval; I^2^ = Inconsistency index; PI = Prediction interval; N = number of people.

*Population health outcome: Epidemiologic impact:* C19ST identified 31 (95% confidence interval [CI] 14–65) new cases per 1000 individuals across 40 data sets (21,056,338 participants; Fig. 2 and Table 3) (Table 1). Most participants (21,048,121 individuals; 29 datasets) underwent routine interval testing; 7198 participants were tested once (from nine data sets); and two data sets with 1019 study participants had an unclear testing frequency (Supplement Text S8). Case detection was higher among mixed symptomatic/asymptomatic study populations (51 [95% CI 21–121] cases per 1000 individuals), compared to strictly asymptomatic populations (9 [95% CI 3–24] cases per 1000 individuals) (Supplement Text S8). In five data sets with all individuals also being tested by RT-PCR (458 mixed symptomatic/asymptomatic RT-PCR positive individuals), C19ST missed 14% (95% CI 1–65%) of cases (Fig. 2 and Table 3, with further details in Supplement Text S9).

**Table 3:** Results of the quantitatively meta-analysed outcomes.

Outcome	Point estimate (95% confidence interval)	I^2^	Prediction interval	Studies included
Case detection	31 (14–65) per 1000 individuals	100.0	0–814 per 1000 individuals	24, 25, 26^,^^28^^,^^30^^,^^32^^,^^34^^,^^35^^,^^37^^,^^40^^,^^45^, ^46^, ^47^, ^48^, ^49^, ^50^^,^^55^^,^^58^^,^^63^, ^64^, ^65^, ^66^, ^67^^,^^69^^,^^72^^,^^75^^,^^79^^,^^80^
Missed cases proportion	14% (1–65%)	98.8	0–96%	^34^^,^^40^^,^^64^^,^^67^
Test positivity	7 (3–15) per 1000 tests NNT: 145 (67–315)	100.0	0–461 per 1000 tests NNT: 2–17,587	^24^^,^^25^^,^^29^^,^^30^^,^^32^^,^^34^^,^^37^, ^38^, ^39^^,^^41^^,^^44^^,^^47^^,^^51^^,^^55^^,^^59^^,^^63^, ^64^, ^65^^,^^67^, ^68^, ^69^, ^70^^,^^74^, ^75^, ^76^^,^^79^
False positive proportion	0.4% (0.2–1.0%)	97.3	0–6%	^34^^,^^39^^,^^41^^,^^60^^,^^64^
Invalid test proportion	0.1% (0.0–0.4%)	99.5	0–40%	^24^^,^^25^^,^^29^^,^^30^^,^^32^^,^^37^, ^38^, ^39^^,^^41^^,^^47^^,^^51^^,^^63^^,^^64^^,^^68^^,^^70^^,^^76^^,^^79^
Test uptake	57% (43–70%)	99.8	8–96%	^24^^,^^26^^,^^32^^,^^37^^,^^43^^,^^50^^,^^51^^,^^55^, ^56^, ^57^, ^58^^,^^63^^,^^68^^,^^69^^,^^71^^,^^77^^,^^78^^,^^82^, ^83^, ^84^
Test schedule adherence	76% (66–84%)	100.0	16–98%	^24^^,^^30^^,^^32^^,^^35^^,^^36^^,^^38^^,^^41^^,^^45^^,^^47^^,^^49^^,^^50^^,^^52^^,^^55^^,^^57^^,^^59^^,^^63^, ^64^, ^65^^,^^68^^,^^69^^,^^72^
Result reporting	89% (78–95%)	99.9	16–100%	^24^^,^^26^^,^^30^^,^^32^^,^^35^^,^^37^^,^^38^^,^^41^^,^^43^^,^^46^^,^^49^^,^^50^^,^^52^^,^^56^^,^^58^^,^^62^, ^63^, ^64^^,^^69^^,^^79^

NNT = number needed to test.

**Fig. 3:**
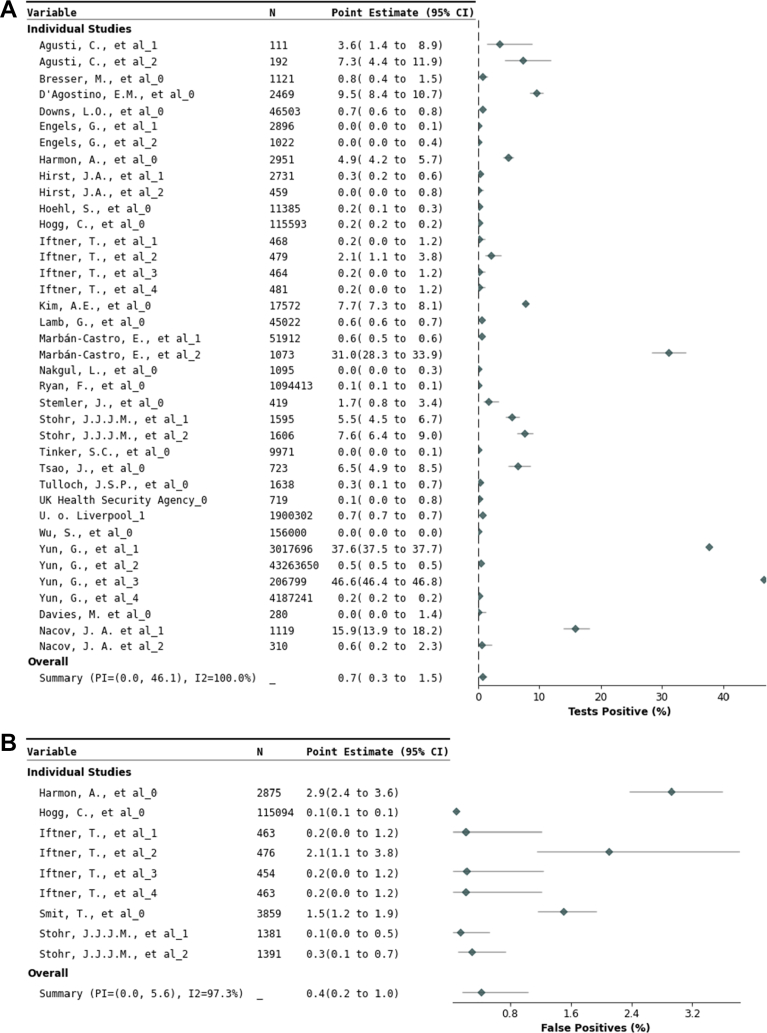
**Forest plots test-positivity and proportion of false-positive COVID-19 self-tests.** Forest plots of A) test positivity: the test positivity proportion of all individual C19ST results and B) false positives: the proportion of negative reverse-transcriptase polymerase chain reaction test results that falsely were positive using COVID-19 self-testing. CI = Confidence interval; I^2^ = Inconsistency index; PI = Prediction interval; N = number of tests.

Test positivity across 38 data sets comprising 54,150,480 C19STs was 7 (95% CI 3–15) positive results per 1000 tests (Fig. 3 and Table 3), corresponding to an overall NNT of 145 (95% CI 67–315). Lower positivity compared to case detection likely reflects serial testing behaviour, where individuals testing positive discontinue testing, while those testing negative continue. In studies with single-instance testing, test positivity was 2% (95% CI 1–4%), yielding a NNT of 51 (95% CI 23–113) (Supplement Text S10). With twice-weekly testing, test positivity was 0.3% (95% CI 0.1–1.0%), corresponding to a NNT of 379 (95% CI 99–1460) (Supplement Text S10). Subgroup analyses also demonstrated that test positivity was 0.1% (95% CI 0.0–0.3%) in asymptomatic populations and 1.3% (95% CI 0.6–3.0%) in mixed symptomatic/asymptomatic populations, corresponding to NNTs of 1002 (95% CI 332–3026) and 75 (95% CI 33–172), respectively (Supplement Text S10). Across nine datasets where every C19ST was paired with RT-PCR (126,456 negatives), the false-positive proportion was 0.4% (95% CI 0.2–1.0%) (Fig. 3 and Table 3, with further details in Supplement Text S11). Invalid results were reported in 0.1% (95% CI 0.0–0.4%) of 2,191,650 C19STs across 26 data sets (Table 3, with further details in Supplement Text S12). Heterogeneity was very high for all outcomes (I^2^ > 97%).

*Population health outcome: Virus transmission, morbidity and mortality:* In two data sets encompassing 27,883 participants, daily C19ST was used as an alternative to self-isolation following exposure to SARS-CoV-2 cases.^49^^,^^50^ 3 of these self-testing participants became SARS-CoV-2 positive (“primary cases”), and infected another 337 individuals (“secondary cases”). The secondary attack proportion, defined as the proportion of secondary cases out of the primary cases, ranged from 6.2% (95% CI 3.4–11.1%)^50^ to 6.3% (95% CI 5.6–7.0%).^49^ The secondary attack proportion in standard self-isolation groups was similar in one study (7.5% [95% CI 6.7–8.3%]; 393 secondary cases out of 5219 primary cases)^49^ and not directly assessed in the other.^50^ Additional studies evaluating population-level impacts of C19ST on transmission are summarised in the (Supplement Table S2), including studies in elderly care homes and community-based test distribution programs.

*Implementation outcome: Linkage for positive and negative tests*: Of 87 data sets, 48 reported actions following a positive C19ST result. In 25 data sets, positive results were followed by RT-PCR confirmation (Supplement Table S3). This was compulsory or strongly recommended in 23 data sets; however, only three data sets reported RT-PCR results demonstrating that every positive C19ST was confirmed (Supplement Table S3). In two data sets where RT-PCR confirmation was voluntary or unclear, the proportion of individuals who underwent confirmatory testing was not reported (Supplement Table S3). The second most frequently reported action was self-isolation, recommended in 20 data sets (Supplement Table S3). Linkage of negative results was reported in 39 datasets, with negative C19ST results commonly allowing continuation of daily activities or exemption from self-isolation (Supplement Table S4).

**Fig. 4:**
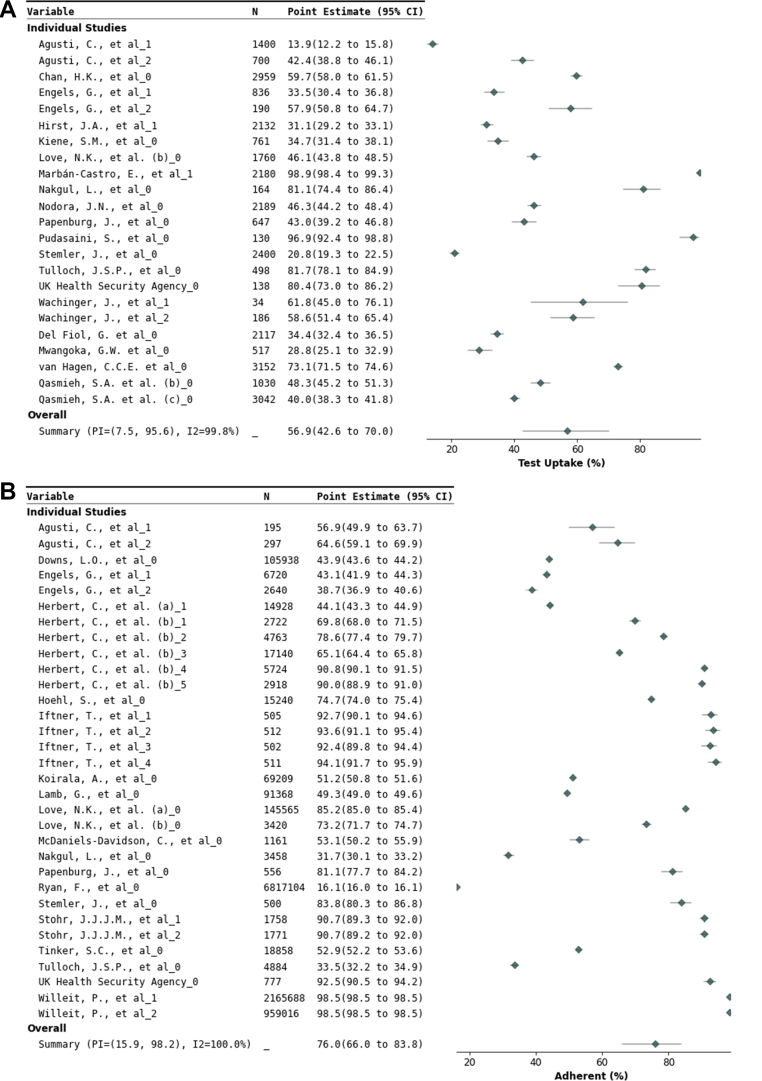

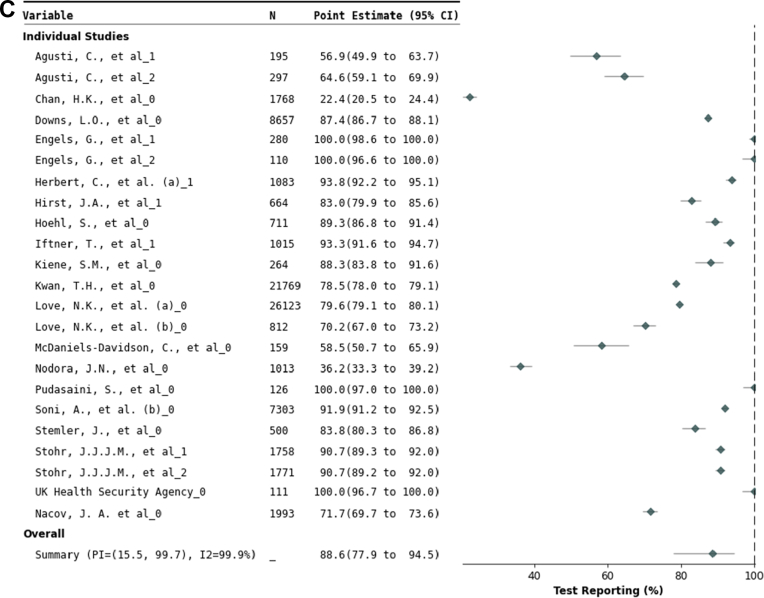
**Forest plots of test uptake proportion, test schedule adherence, and result reporting.** Forest plots of A) test uptake: the proportion of study participants that voluntarily participated in a COVID-19 self-testing (C19ST) study, B) test schedule adherence: the proportion of C19ST tests actually performed relative to those requested per study protocol, and C) test result reporting: the proportion of C19ST results reported to study officials or health authorities. CI = Confidence interval; I^2^ = Inconsistency index; PI = Prediction interval; N = number of people for Part A and number of tests for Part B and C.

*Implementation outcome: Test uptake:* Across 23 data sets offering C19ST voluntarily to 29,162 study participants, pooled uptake was 57% (95% CI 43–70% (Table 3 and Fig. 4, with further details in Supplement Text S13). In mandatory testing study sites,^40^^,^^48^^,^^66^^,^^67^^,^^74^ full compliance was reported.

*Implementation outcome: Test frequency:* Overall adherence to testing schedules was 76% (95% CI 66–84%) across 32 data sets with 4,492,534 C19STs reported (Table 3 and Fig. 4). Adherence was highest for single-instance testing (88% [95% CI 80–93%]; nine data sets with 5815 C19STs), and higher with three or more tests/week (78% [95% CI 64–88%]; five data sets with 174,057 C19STs) versus twice-weekly testing (53% [95% CI 32–73%]; 11 data sets with 3,342,794 C19STs) (Supplement Text S14). Subgroup adherence was comparable in K-12 students and staff (73% [95% CI 45–89%]), healthcare professionals (74% [95% CI 52–89%]), and the general population (81% [95% CI 73–87%]) (Supplement Text S14).

*Implementation outcome: Result reporting:* Across 23 data sets comprising 78,482 study participants, 89% (95% CI 78–95%) reported C19ST results (Table 3 and Fig. 4). Reporting ranged from 22% (95% CI 21–24%) in an employee study^26^ to 100% in child day care centres,^32^ school trips,^58^ and health-care professionals.^69^ An American study found higher reporting with a 28 USD gift card (90–91%) compared to non-incentivised states (70–77%).^36^ Reporting was higher in asymptomatic individuals (96% [95% CI 79–99%]; seven data sets) compared to symptomatic or mixed populations (84% [95% CI 69–92%]; 16 data sets) (Supplement Text S15). Seven additional data sets with complete reporting were excluded from the meta-analysis, as C19ST was either mandatory^40^^,^^48^^,^^66^^,^^67^^,^^74^ or directly supervised.^55^^,^^57^

*Implementation outcome: Other:* Results on outcomes of time to diagnosis and behaviour change were limited and heterogeneous, and are reported in the (Supplement Text S16 and Supplement Table S5).

*Social harm:* No study specifically evaluated social harms, but missed infections and false-positives remain important concerns. In an analysis of ∼3.6 million positive C19STs confirmed by RT-PCR, ∼300,000 (8%) were false-positives, leading to 1.5 million days of unnecessary self-isolation in the absence of confirmatory RT-PCR testing.^33^ Another study reported lower self-isolation and contact notification after a positive C19ST result compared to professional testing.^53^

*Broader societal effects:* In ten data sets, C19ST facilitated school and workplace operations by either reducing the duration of self-isolation^45^^,^^49^^,^^51^^,^^54^^,^^69^^,^^70^^,^^74^ or by enabling activities contingent on a negative result.^34^^,^^58^^,^^76^ One of these studies estimated that replacing self-isolation (after contact with a SARS-CoV-2 case) with daily C19ST reduced work-absenteeism by an average of 5.9 days per contact,^49^ and another similarly reported shortened self-isolation time.^50^ However, another study reported that voluntary twice-weekly self-testing of care-home staff did not affect workplace-absenteeism compared to self-testing only in the event of staff experiencing symptoms of larger outbreaks.^81^ Ten data sets reported that C19ST improved well-being^24^^,^^26^^,^^32^^,^^43^^,^^51^^,^^58^^,^^71^^,^^86^^,^^87^ including greater peace of mind,^51^ perceived safety,^71^ reduced fear of infection,^26^^,^^58^ and appreciation for the privacy afforded by self-testing.^24^ Data on C19ST resource utilisation was limited and varied widely across studies (Supplement Table S6).

## Discussion

This systematic review and meta-analysis provides, to our knowledge, the most comprehensive synthesis to date of population health and implementation outcomes of C19ST. Our findings demonstrate that widespread self-testing can enhance case detection and support pandemic control efforts. While C19ST missed cases, current evidence strongly supports its role as a complementary diagnostic tool to RT-PCR. The convenience and public acceptance of C19ST contributed to reductions in self-isolation, workplace absenteeism, and other societal disruptions, enabling greater social functioning during the pandemic. These findings support the inclusion of self-testing strategies in early response frameworks for future pandemics. However, the overall low quality of included studies and substantial heterogeneity across outcomes limit generalisability and underscore the need for standardised evaluation frameworks.

We estimated that 31 per 1000 individuals (95% CI 14–65) conducting C19ST were positive for SARS-CoV-2, with higher detection in symptomatic individuals. In asymptomatic populations, case detection was 9 per 1000 (95% CI 3–24), comparable to the 3 (95% CI 2–3) per 1000 pooled RT-PCR positivity reported in a prior meta-analysis.^88^ Given that C19ST does not require clinical infrastructure, its performance in this context is encouraging. The NNT to identify one positive was 75 (95% CI 33–172) in symptomatic individuals and 1002 (95% CI 332–3026) in asymptomatic populations with a pooled NNT of 145 (95% CI 67–315), suggesting a modest testing burden to support public health impact. Nonetheless, in paediatric studies pooled saliva or gargle wash RT-PCR testing^32^^,^^42^ was preferred and more sensitive, but required greater infrastructure capacity. However, while large-scale pooled testing might be a feasible approach to SARS-CoV-2 screening, it has so far only been implemented in select contexts, such as Vienna, Austria.^89^

C19ST missed an estimated 14% (95% CI 1–65%) of COVID-19 cases relative to RT-PCR. While this false negative rate is lower than anticipated based on pooled antigen test sensitivities (∼60–80%),^5^^,^^90^, ^91^, ^92^ it still reflects the inherent limitations of LFIA-based testing. Serial testing likely mitigated this discrepancy by capturing initially missed cases.^93^ Notably, the comparable case detection rates between C19ST and pooled RT-PCR at the programmatic level, as discussed above, support the effectiveness of C19ST as a screening tool, despite the lower sensitivity of antigen-based tests compared to RT-PCR in individual head-to-head comparisons. False positives were infrequent: among paired C19ST and RT-PCR datasets, 0.4% (95% CI 0.2–1.0%) were false positives, consistent with prior reports of specificity ≥99%.^5^ However, even low false positive rates can cause disruptions when prevalence is low. One study estimated that false positive C19STs led to 1.5 million days of unnecessary self-isolation,^33^ highlighting a well-recognised limitation of diagnostic testing: even highly specific assays can yield more false positives than true positives when pre-test probability is low,^94^ underscoring the importance of confirmatory testing to minimise harm.

C19ST uptake (57% [95% CI 43–70%]), result reporting (89% [95% CI 78–95%]), and test schedule adherence (76% [95% CI 66–84%]) were high across studies. However, estimates reflect an unavoidable selection bias, as data were drawn from individuals who agreed to participate in research and are likely more engaged than typical C19ST users, contributing to the low-quality ratings of these outcomes and limiting generalisability. Most tests were also provided free of charge; the impact of cost on uptake and adherence remains unclear. Real-world performance may differ, particularly in unsupervised settings or with financial barriers. Additionally, among studies that required confirmatory testing, few reported how many participants actually obtained confirmatory tests, limiting our understanding of adherence to recommended follow-up actions. From a societal perspective, C19ST enabled rapid results (10–30 min),^35^^,^^73^ which informed isolation, permitted continued activities (e.g., work or travel),^34^^,^^49^^,^^70^^,^^76^ and improved perceptions of safety.^24^^,^^58^^,^^71^ Although few studies compared secondary attack proportions between self-isolation and the use of C19ST after contact with a SARS-CoV-2 case, the available data suggest that secondary attack rates were similar. However, lower adherence to isolation and contact notification compared to professionally administered testing^53^ highlights the need for public health measures to reinforce these behaviours.

Strengths of this review include the comprehensive synthesis of population health outcomes, implementation metrics, and societal outcomes across diverse settings using rigorous, policy-driven methods of both peer-reviewed and pre-print studies through October 1st, 2025. However, we did not identify studies conducting a robust causal comparison between settings using professional testing alone and those incorporating C19ST with professional testing, limiting our ability to assess its incremental impact. Furthermore, few studies included were conducted in low- and middle-income countries (LMICs), limiting the generalisability of our findings to these settings. This aligns with prior reports documenting reduced access to and coverage of C19ST in LMICs compared to high-income countries,^95^ highlighting the need to strengthen testing and research capacity in low-resource settings. Our analysis was further limited by substantial heterogeneity (I^2^ > 97% across meta-analysed outcomes), likely reflecting variability in study design, Ag-RDT performance,^90^^,^^96^ and study context, including SARS-CoV-2 incidence, prevalence and transmission dynamics. I^2^ may also appear inflated by large studies with low within-study variance, which amplify between-study differences. We therefore report prediction intervals, which confirmed wide data dispersion, consistent with visual forest plot inspection. Moreover, while antigen-based diagnostics have been shown to be effective in detecting SARS-CoV-2 across different age groups and specimen types, data in our systematic review was too limited to assess the impact of C19ST in these groups in detail.^97^^,^^98^ Lastly, test uptake, adherence, and result reporting were limited by selection bias, and sensitivity analyses could not be conducted because only individuals who consented to study participation were included.

In conclusion, large-scale C19ST implementation can improve case detection with high uptake, testing adherence, and timely reporting in study settings. Despite limited generalisability, C19ST demonstrated reasonable diagnostic accuracy and tangible societal benefits, including reduced social disruption and enhanced perceptions of safety. However, we were unable to assess incremental case detection beyond standard professional testing or to quantitatively synthesise cost-effectiveness, resource utilisation, or linkage to care. These gaps highlight priorities for future research and reinforce the need for standardised methods building on established frameworks^99^^,^^100^ to evaluate the population health and programmatic impact of novel diagnostic tests and strategies. Based on the demonstrated benefits during the COVID-19 pandemic, it may be reasonable to consider antigen-based rapid diagnostic self-tests as a core component of pandemic preparedness and their further integration into structured public health programs.

## Contributors

CMD, RAL, NS, JC, JAS, and CCJ conceived the study. LEB, VF, SM, SK, SY, NRP, BE, AM, and SO further refined the study plan. LEB, VF, AMZ, KW, CE, HT, MG, and RAL performed the literature search, study assessment, and data extraction. LEB, VF, SM, RAL, and CMD accessed and verified the data and performed initial analyses. LEB, VF, SM, RAL, and CMD further wrote the first draft and further improved the manuscript and analysis based on additional comments. All authors provided comments on the manuscript and analyses and approved the final version for submission.

## Data sharing statement

Data extracted from included studies and used for all analysis is publicly available under https://github.com/stmcg/covid-testing-impact-ma-2025. Any questions concerning the data can be directed towards the corresponding author.

## Declaration of interests

LEB is a scientific editor of the Public Health Forum magazine. SO, AM, and BE were employees of FIND, the global alliance for diagnostics, Geneva, Switzerland, while contributing to this manuscript. JAS is currently employed by Roche Diagnostics International, Switzerland, but her input to the overall study design was completed previously, while holding a position at the World Health Organization (WHO), Geneva, Switzerland. NRP is a part-time scientific advisor to the National Institutes of Health (NIH), Bethesda, Maryland, US and the National Institute of Biomedical Imaging and Bioengineering, Bethesda, Maryland, US. NRP was also a visiting professor at the Washington University, St. Louis, Missouri, US (2025) and a lecturer at the Eastern Pennsylvania American Society for Microbiology, US (January 2026). CMD is a member of the WHO advisory group in tuberculosis Diagnostics and Laboratory strengthening as well as vice-chair of the Heidelberg site of the Germany Center for Infection Research (DZIF) and co-chair of the DZIF's tuberculosis working group. CMD is also an editor of the PLOS Medicine magazine. RAL is a full-time employee of the National Institutes of Health, Bethesda, Maryland, US. All other authors declare that they have no competing interests.

## Appendix A Supplementary data

Second revision supplements Impact of C19ST

Renamed_612de
