# Supplementary material for: Population health and implementation outcomes of self-testing for SARS-CoV-2 using antigen detecting diagnostics: a systematic review and meta-analysis
Source: eClinicalMedicine. 2026 Apr 7;94:103838. doi: 10.1016/j.eclinm.2026.103838 (PMC13087455; doi:10.1016/j.eclinm.2026.103838)
Supplement: Second revision supplements Impact of C19ST [file mmc1.docx]

**APPENDIX**

Table of Contents

[Text S1 – Study protocol 2](#_Toc221336324)

[Text S2 – Methods: details of the study inclusion criteria 8](#_Toc221336325)

[Text S3 – Methods: search algorithm and results 9](#_Toc221336326)

[Text S4 – Methods: interpretation guide to the JBI tool for quasi-experimental studies 34](#_Toc221336327)

[Text S5 – Methods: details concerning the use of a binomial-normal generalized linear mixed model for meta-analysis 40](#_Toc221336328)

[Text S6 – Methods: details of subgroup analysis and cost adjustments 42](#_Toc221336329)

[Text S7 – Results: assessment of publication bias 43](#_Toc221336330)

[Text S8 – Results: subgroup analysis of the outcome case detection 45](#_Toc221336331)

[Text S9 – Results: subgroup analysis of the outcome missed cases proportion 48](#_Toc221336332)

[Text S10 – Results: subgroup analysis of the outcome test positivity 51](#_Toc221336333)

[Text S11 – Results: subgroup analysis of the outcome false positives 54](#_Toc221336334)

[Text S12 – Results: analysis of the outcome invalid test proportion 57](#_Toc221336335)

[Text S13 – Results: subgroup analysis of the outcome test uptake 60](#_Toc221336336)

[Text S14 – Results: subgroup analysis of the outcome test schedule adherence 62](#_Toc221336337)

[Text S15 – Results: subgroup analysis of the outcome result reporting 65](#_Toc221336338)

[Text S16 – Results: COVID-19 self-testing time to diagnosis 67](#_Toc221336339)

[Table S1 – List of studies excluded 68](#_Toc221336340)

[Table S2 – Results: Impact of C19ST on virus transmission, morbidity and mortality 69](#_Toc221336341)

[Table S3 – Results: Linkage after positive COVID-19 self-test result 72](#_Toc221336342)

[Table S4 – Results: Linkage after negative COVID-19 self-test result 74](#_Toc221336343)

[Table S5 – Results: Behaviour changes due to C19ST 76](#_Toc221336344)

[Table S6 – Results: COVID-19 self-testing resource usage 78](#_Toc221336345)

[Figure S1 – Results: Summary of the quality assessment 79](#_Toc221336346)

[Sources 80](#_Toc221336347)

# Text S1 – Study protocol

December 11, 2021

**Systematic review on the impact of self-performed Ag-RDTs for SARS-CoV-2 in
populations seeking testing**

Lukas E. Brümmer, Hannah Tolle, Stephan Katzenschlager, Christian Erdmann, Ioana D. Olaru, Maurizio Grilli, Nira R. Pollock, Claudia M. Denkinger

*Updated approach 2025. To further align the analysis’ title and outcomes assessed with the evidence to decision framework of the World Health Organization, the manuscript will be published under the title “Population health and implementation outcomes of self-testing for SARS-CoV-2 using antigen detecting diagnostics: a systematic review and meta-analysis”. Furthermore, due to multiple updates to the initial search and analysis requiring the support of further researchers, the following individuals were added to the list of authors: Verena Faehling, Sean McGrath, Ana-Mihaela Zorger, Karina Worbes, Seda Yerlikaya, Berra Erkosar, Aurelien Mace, Stefano Ongarello, Cheryl C. Johnson, Jilian A. Sacks, Jane Cunningham, Nicole Skoetz, Rose A. Lee. The support of Ioana D. Olaru is recognized in the acknowledgements.*

**BACKGROUND**

With the COVID-19 pandemic continuing to inflict a burden on societies worldwide, self-performed antigen rapid diagnostic tests (Ag-RDTs) for SARS-CoV-2 could be an additional tool to reduce transmission. Self-testing has already been recommended by the World Health Organization (WHO) for various other infectious diseases [1,2]. For SARS-CoV-2, self-testing has a role primarily in the screening of asymptomatic individuals and has been employed for testing to protect (e.g. in high-risk settings such as hospitals), to release (e.g. contact testing) and to enable (e.g. regular school or workplace testing) as well as mass-testing (e.g. in Germany). However, the impact has not been systematically evaluated to date. With this systematic review, we aim to assess the impact self-performed Ag-RDTs for SARS-CoV-2 could have on the COVID-19 pandemic.

**PICO QUESTIONS**

What is the impact (O) of implementing self-performed Ag-RDTs for SARS-CoV-2 (I) in populations during the COVID-19 pandemic (P) compared to when no testing or testing with professional use only is performed (C)?

P: persons during the COVID-19 pandemic

I: Self-performed Ag-RDTs for SARS-CoV-2

C: No testing or testing with professional use only performed

O: listed below

**OUTCOMES**

Outcomes to be ranked by GDG and classified into primary and secondary outcomes

1. Community level health impact
   1. Changes in the transmission of SARS-CoV-2, measured in SARS-CoV-2 incidence, prevalence, cases averted, secondary contacts and secondary cases
   2. Changes in testing uptake / acceptability
2. Individual level health impact
   1. Changes in COVID-19 morbidity, measured in hospitalization, disease severity
   2. Changes in COVID-19 mortality, measured in deaths averted
   3. Changes in proportion of results reported and test-positivity rate
   4. Changes in time to diagnosis and time to isolate
   5. Changes in proportion taking following action upon receiving test results:
      1. Positive result: linkage with confirmatory testing
      2. Negative result: adherence to public health security measurements (PSHM) and / or seeking testing for other diseases if symptomatic
   6. Changes in any other measurements upon receiving test result
3. Effects on the health system (hospitalization, ICU occupancy and/or transmission within health care workers)
4. Effects on not health related aspects: absenteeism, schooling, resource usage, user costs (including opportunity costs) and time-savings
5. Misuse as use out of the intended or fabrication of data, i.e., reporting false results
6. Adverse events
7. Social harms

*Updated approach 2025. To further align the analysis’ results with the evidence to decision framework of the World Health Organization, the outcomes assessed will be clustered in the groups of “population health outcomes” and “implementation outcomes” (see Table 1 of the main manuscript).*

**INCLUSION CRITERIA**

To be included in the review, an article must meet the following criteria:

1. Study designs that implemented a self-performed Ag-RDT for SARS-CoV-2 in a population seeking testing during the COVID-19 pandemic
2. Measured one or more of the outcomes listed above
3. Study sample size greater 100

No restrictions will be placed based on location of the intervention. We will consider retrospective or prospective cohort or nested cohort studies, case-control or cross-sectional studies, before and after studies, as well as randomized studies.

**VAIRABLES CONSIDERED**

When possible, we will stratify outcomes and present by the following categories.

1. Frequency of testing
   1. Once (without a fixed interval)
   2. Routine, i.e., more than 1 occasion as per a fixed interval
2. SARS-CoV-2 exposure
   1. No known exposure
   2. Known exposure – single
   3. Known exposure – frequent
   4. Unknown / not defined
3. Test distributor: workplace, school/university, publicly available (i.e., “Buergertests)
4. Location of testing: work, home, school/university
5. Costs for persons to be tested: tests free of charge vs. provision required
6. Setting:
   1. Rural or urban,
   2. GDP (categorized by WTO)
   3. literacy of the target population
7. Ag-RDT used: company name, test and lot number (if available), sample type
8. Test assistance: testing was assisted by trained operator, was assisted virtually by trained operator, was assisted in any other way or was not assisted at all
9. Risk level of COVID-19 related morbidity and mortality: as defined by paper
10. Vaccination status: vaccinated vs. unvaccinated
11. Seroconvalescence status: convalescent vs. uninfected
12. Age: persons ≤ 18 years of age, persons 18 – 65 years of age, persons > 65 years of age
13. Misuse, adverse events, social harms: specific to self-testing vs. issues that occur in general for SARS-CoV-2 testing
14. Additional interventions: Any other interventions that were implemented to stop transmission besides self-performed Ag-RDTs for SARS-CoV-2, such as mask wearing, isolation / social distancing practices, travel restrictions

**SEARCH STRATEGY**

The following electronic databases will be searched for any article published between December 1, 2020 and November 30, 2021: PubMed and Web of Science. Secondary reference searching will also be conducted on all studies included in the review. Further, selected experts in the field will be contacted to identify additional articles not identified through other search methods.

*Updated approach 2025. The search will be conducted from December 1^st^, 2020 until October 1^st^, 2025. To increase comprehensiveness of the review, the electronic databases Embase, medRxiv, clinicaltrials.gov and the Cochrane Library will also be searched.*

The main search terms will be “Severe Acute Respiratory Syndrome Coronavirus 2”, “COVID-19”, “Betacoronavirus”, Coronavirus”, “Self-testing” and “Antigen”. Details on the search algorithm can be found in the appendix. No language restrictions will be applied. Articles in languages other than English will be translated where necessary.

**STUDY SELECTION**

Two reviewers (LEB and HT) will review the titles and abstracts of all publications identified by the search algorithm independently. Afterwards, they will individually conduct a full-text review for those eligible, to select the articles for inclusion in the systematic review. As a final step, they will compare results with each other and a third reviewer (CE). Any disputes will be solved by discussion or by a fourth reviewer (CMD).

*Updated approach 2025. Title and abstract screening will be performed by LEB and either AMZ or VF. Full-text review will be performed by LEB and either AMZ, KW, or VF. The senior reviewer to resolve disputes will be RL.*

**DATA EXTRACTION AND MANAGEMENT**

Data extraction will be performed by one reviewer, using a standardized Google Sheets form, and controlled by a second. Differences in data extraction will be resolved through consensus or referral to a senior study team member. Studies that assessed multiple self-performed Ag-RDTs or presented results based on differing parameters (e.g., multiple study locations) will be considered as individual datasets.

The following information will be extracted from each included study:

1. Study identification: Author(s), title, peer-review status, year of publication
2. Study characteristics: type of study, start date of study, end date of study, study location, frequency of testing, test distributor, location of testing, setting, test assistance, rural or urban setting, income, literacy
3. Population characteristics: SARS-CoV-2 exposure, risk factors, vaccination status, history of COVID-19 infection, age, number of females
4. Transmission outcomes: Pre- and post-incidence, -prevalence, -number of hospitalizations, -disease severity, -cases averted, -deaths averted
5. Testing characteristics: Ag-RDT used, sample type, persons tested, number of tests done, number of positive test results, number of invalid test results, test uptake, time to diagnosis, time to isolate, measurements upon receiving test results
6. Other effects: effects on school operations, on quarantine duration, on people’s well-being, absenteeism, resource usage, user costs (including opportunity costs), time-savings and effects on the health system (e.g. ICU bed occupancy)
7. Additional interventions: description of measurements taken besides self-performed Ag-RDTs (both within the study and other general measurements
8. Undesirable effect associated with testing: use out of the intended, fabrication of data, social harm, adverse events

**RISK OF BIAS**

To assess the quality of the included studies, we will employ the Newcastle-Ottawa Scale. The tool consists out of eight questions grouped into three categories (selection, comparability and exposure). Depending on the study’s quality, stars are assigned to each of the questions, leading to an overall zero-star-rating (worst) to nine-star-rating (best) each study [3]. Risk of bias assessment will be performed by two reviewers (LEB and HT) independently.

*Updated approach 2025. A quality assessment will be performed instead of a Risk of Bias assessment, utilizing the revised Joanna Briggs Institute (JBI) Critical Appraisal Tool for Quasi-Experimental Studies [4]. The quality assessment will be conducted by LEB and either HT, AK, RL, or VF, with disputes being resolved by discussion.*

**GRADING OF EVIDENCE**

Following the GRADE approach [5], if evidence from randomized controlled trials is limited, evidence from non-randomized but controlled studies or observational studies will be used instead, but also downgraded per the GRADE system. Grading of evidence will be performed by two reviewers (LEB and HT) independently and presented via GRADE evidence profiles.

*Updated approach 2025. A grading of evidence will not be performed.*

**DATA ANALYSIS**

For community and individual level impact (outcome 1-2), we will prepare forest plots and visually evaluate the heterogeneity between studies. We will assess heterogeneity between studies and assess whether a meta-analysis is feasible. We aim to provide point estimates for changes in incidence, prevalence, number of hospitalizations, disease severity, cases averted and deaths averted along with 95% confidence intervals using a random effects model. Also, for health system and non-health related effects (outcomes 3-4), misuse (outcome 5), adverse events (outcome 6) and social harms (outcome 7) a descriptive analysis will be performed.

**SOURCES FOR THE STUDY PROTOCOL**

1. World Health Organization. Guidelines on HIV self-testing and partner notification: supplement to consolidated guidelines on HIV testing services. 2016.

2. World Health Organization. Recommendations and guidance on hepatitis C virus self-testing. 2021.

3. Wells G, Shea B, O’Connell D, Peterson J, Welch V, Losos M, et al. The Newcastle-Ottawa Scale (NOS) for assessing the quality of nonrandomised studies in meta-analyses. 2013.

4. Barker TH, Habibi N, Aromataris E, Stone JC, Leonardi-Bee J, Sears K, et al. The revised JBI critical appraisal tool for the assessment of risk of bias for quasi-experimental studies. JBI Evid Synth. 2024;22(3):378-88

5. Higgins JPT, Thomas J, Chandler J, Cumpston M, Li T, Page MJ, et al. Cochrane Handbook for Systematic Reviews of Interventions version 6.2 (updated February 2021). 2021.

# Text S2 – Methods: details of the study inclusion criteria

For a study to qualify as self-testing, participants had to independently perform all steps, including sample collection, test execution, and result interpretation. In studies involving children, assistance from parents or guardians was permitted, but not from professional healthcare workers. We contacted study authors when it was unclear whether all testing steps were conducted by participants themselves, and excluded studies in which self-testing could not be confirmed.

# Text S3 – Methods: search algorithm and results

**First search – December 1^st^ 2020 until March 14^th^ 2023**

Main concepts definition

P

| SARS-CoV-2 |  |
| --- | --- |

I

| self-testing |  |
| --- | --- |

Strategy

| 1 | P |  |
| --- | --- | --- |
| 2 | I |  |
| 3 | 1 AND 2 |  |

**Limit:** Publication from 01.12.2020

Databases

- PubMed
- EMBASE
- Web of Science Core Collection
- medRxiv (via Europe PMC)
- Databases (Via WHO COVID-19 Database):

PREPRINT-RESEARCHSQUARE

PREPRINT-SSRN

MDPI

Academic Search Complete

COVIDWHO

PREPRINT-ARXIV

GIM

Centers for Disease Control and Prevention

CAB Abstracts

LILACS

Results report

The results were saved in Endnote and deduplicated. Some articles could still appear more than once.

The hits are sorted by database in Endnote. The PubMed hits were the first to be exported in Endnote. This makes them preferred for deduplication. In other words, in the case of duplicates, entries are removed from other databases.

The number of hits for each database in this report is based on its pre-deduplication status.

PubMed

| **Trefferzahl** | **Datum** |
| --- | --- |
| 5819 | 14.03.2023 |

P

| **"Coronavirus"[Mesh] OR**  **"COVID-19"[Mesh] OR**  covid*[tw] OR  coronavirus*[tw] OR  "corona virus*"[tw] OR  betacoronavirus*[tw] OR  ncov*[tw] OR  "n cov*"[tw] OR  2019nCoV*[tw] OR  nCoV2019[tw] OR  "HCoV 19"[tw] OR  HCoV19[tw] OR  Ncovor[tw] OR  Ncorona*[tw] OR  sars2*[tw] OR  "sars 2*"[tw] OR  sarscov*[tw] OR  "sars cov*"[tw] | 361867 |
| --- | --- |

I

| **"Self-Testing"[MeSH] OR**  selftest*[tw] OR  "self test*"[tw] OR  ((home*[tw] OR  self*[tw] OR  personal*[tw] OR  mail*[tw] OR  screen*[tw] OR  serial*[tw] OR  mass*[tw])  AND  **("COVID-19 Testing"[Mesh] OR**  test*[tw] OR  swab*[tw] OR  detection[tw])  AND  **("Antigens"[MeSH Terms] OR**  antigen*[tw] OR  nasal*[tw] OR  nasopharyngeal*[tw] OR  oropharyngeal*[tw] OR  oral*[tw] OR  saliva*[tw] OR  "lateral flow*"[tw] OR  LFA[tw] OR  LFAs [tw] OR  LFD [tw] OR  LFDs [tw] OR  RALFT[tw] OR  RDT[tw] OR  RDTs[tw] OR  RATs[tw] OR  RAT[tw] OR  rapid*[tw] OR  "point of care*"[tw])) | 222423 |
| --- | --- |

**String (title, abstract, keywords)**

As above

P AND I: 7447

Ab 01.12.2020: 5819

Embase

| **Trefferzahl** | **Datum** |
| --- | --- |
| 1499 | 14.03.2023 |

P

| **'Coronavirinae'/exp OR**  **'Coronavirus infection'/exp OR**  covid*:ti,ab,kw OR  coronavirus*:ti,ab,kw OR  "corona virus*":ti,ab,kw OR  betacoronavirus*:ti,ab,kw OR  ncov*:ti,ab,kw OR  "n cov*":ti,ab,kw OR  2019nCoV*:ti,ab,kw OR  nCoV2019:ti,ab,kw OR  "HCoV 19":ti,ab,kw OR  HCoV19:ti,ab,kw OR  Ncovor:ti,ab,kw OR  Ncorona*:ti,ab,kw OR  sars2*:ti,ab,kw OR  "sars 2*":ti,ab,kw OR  sarscov*:ti,ab,kw OR  "sars cov*":ti,ab,kw | 427585 |
| --- | --- |

I

| **'self-testing'/exp OR**  selftest*:ti,ab,kw OR  "self test*":ti,ab,kw OR  ((home*:ti,ab,kw OR  self*:ti,ab,kw OR  personal*:ti,ab,kw OR  mail*:ti,ab,kw OR  screen*:ti,ab,kw OR  serial*:ti,ab,kw OR  mass*:ti,ab,kw)  AND  **('COVID-19 testing'/exp OR**  test*:ti,ab,kw OR  swab*:ti,ab,kw OR  detection:ti,ab,kw)  AND  **('antigen'/exp OR**  "antigen*":ti,ab,kw OR  nasal*:ti,ab,kw OR  nasopharyngeal*:ti,ab,kw OR  oropharyngeal*:ti,ab,kw OR  oral*:ti,ab,kw OR  saliva*:ti,ab,kw OR  "lateral flow*":ti,ab,kw OR  LFA:ti,ab,kw OR  LFAs:ti,ab,kw OR  LFD:ti,ab,kw OR  LFDs:ti,ab,kw OR  RALFT:ti,ab,kw OR  RDT:ti,ab,kw OR  RDTs:ti,ab,kw OR  RATs:ti,ab,kw OR  RAT:ti,ab,kw OR  rapid*:ti,ab,kw OR  "point of care*":ti,ab,kw)) | 285981 |
| --- | --- |

**String (title, abstract, keywords)**

As above

P AND I: 9143

**Limit without PubMed**

NOT ([medline]/lim OR [pubmed-not-medline]/lim): 4166

**Limit document types not indexed in PubMed**

NOT ('Conference Abstract'/it OR 'Note'/it OR 'chapter'/it): 1675

**Limit Publication Year**

AND [01-12-2020]/sd NOT [15-03-2023]/sd: 1499

Web of Science Core Collection

| **Trefferzahl** | **Datum** |
| --- | --- |
| 3299 | 14.03.2023 |

P

| covid* OR  coronavirus* OR  "corona virus*" OR  betacoronavirus* OR  ncov* OR  "n cov*" OR  2019nCoV* OR  nCoV2019 OR  "HCoV 19" OR  HCoV19 OR  Ncovor OR  Ncorona* OR  sars2* OR  "sars 2*" OR  sarscov* OR  "sars cov*" |  |
| --- | --- |

I

| selftest* OR  "self test*" OR  ((home* OR  self* OR  personal* OR  mail* OR  screen* OR  serial* OR  mass*)  AND  (test* OR  swab*” OR  detection”)  AND  (antigen* OR  nasal* OR  nasopharyngeal* OR  oropharyngeal* OR  oral* OR  saliva* OR  "lateral flow*" OR  LFA OR  LFAs OR  LFD OR  LFDs OR  RALFT OR  RDT OR  RDTs OR  RATs OR  RAT OR  rapid* OR  "point of care*")) |  |
| --- | --- |

**String (title, abstract, keywords)**

**P (342289)**

TI=(covid* OR coronavirus* OR "corona virus*" OR betacoronavirus* OR ncov* OR "n cov*" OR 2019nCoV* OR nCoV2019 OR "HCoV 19" OR HCoV19 OR Ncovor OR Ncorona* OR sars2* OR "sars 2*" OR sarscov* OR "sars cov*")

OR

AB=(covid* OR coronavirus* OR "corona virus*" OR betacoronavirus* OR ncov* OR "n cov*" OR 2019nCoV* OR nCoV2019 OR "HCoV 19" OR HCoV19 OR Ncovor OR Ncorona* OR sars2* OR "sars 2*" OR sarscov* OR "sars cov*")

OR

AK=(covid* OR coronavirus* OR "corona virus*" OR betacoronavirus* OR ncov* OR "n cov*" OR 2019nCoV* OR nCoV2019 OR "HCoV 19" OR HCoV19 OR Ncovor OR Ncorona* OR sars2* OR "sars 2*" OR sarscov* OR "sars cov*")

**I (114572)**

TI=(selftest* OR "self test*" OR ((home* OR self* OR personal* OR mail* OR screen* OR serial* OR mass*) AND (test* OR swab*” OR detection”) AND (antigen* OR nasal* OR nasopharyngeal* OR oropharyngeal* OR oral* OR saliva* OR "lateral flow*" OR LFA OR LFAs OR LFD OR LFDs OR RALFT OR RDT OR RDTs OR RATs OR RAT OR rapid* OR "point of care*")))

OR

AB=(selftest* OR "self test*" OR ((home* OR self* OR personal* OR mail* OR screen* OR serial* OR mass*) AND (test* OR swab*” OR detection”) AND (antigen* OR nasal* OR nasopharyngeal* OR oropharyngeal* OR oral* OR saliva* OR "lateral flow*" OR LFA OR LFAs OR LFD OR LFDs OR RALFT OR RDT OR RDTs OR RATs OR RAT OR rapid* OR "point of care*")))

OR

AK=(selftest* OR "self test*" OR ((home* OR self* OR personal* OR mail* OR screen* OR serial* OR mass*) AND (test* OR swab*” OR detection”) AND (antigen* OR nasal* OR nasopharyngeal* OR oropharyngeal* OR oral* OR saliva* OR "lateral flow*" OR LFA OR LFAs OR LFD OR LFDs OR RALFT OR RDT OR RDTs OR RATs OR RAT OR rapid* OR "point of care*")))

**P AND I: 4129**

**Publication Year**

from 01.12.2020: 3299

MedRxiv (via Europe PMC)

<https://europepmc.org/>

| **Records number** | **Date** |
| --- | --- |
| 807 | 14.03.2023 |

P

| covid* OR  coronavirus* OR  "corona virus*" OR  betacoronavirus* OR  ncov* OR  "n cov*" OR  2019nCoV* OR  nCoV2019 OR  "HCoV 19" OR  HCoV19 OR  Ncovor OR  Ncorona* OR  sars2* OR  "sars 2*" OR  sarscov* OR  "sars cov*" | 503696 |
| --- | --- |

I

| selftest* OR  "self test*" OR  ((home* OR  self* OR  personal* OR  mail* OR  screen* OR  serial* OR  mass*)  AND  (test* OR  swab* OR  detection)  AND  (antigen* OR  nasal* OR  nasopharyngeal* OR  oropharyngeal* OR  oral* OR  saliva* OR  "lateral flow*" OR  LFA OR  LFAs OR  LFD OR  LFDs OR  RALFT OR  RDT OR  RDTs OR  RATs OR  RAT OR  rapid* OR  "point of care*")) | 182701 |
| --- | --- |

**String (title, abstract, keywords)**

(TITLE_ABS:(covid* OR coronavirus* OR "corona virus*" OR betacoronavirus* OR ncov* OR "n cov*" OR 2019nCoV* OR nCoV2019 OR "HCoV 19" OR HCoV19 OR Ncovor OR Ncorona* OR sars2* OR "sars 2*" OR sarscov* OR "sars cov*")

OR

KW:(covid* OR coronavirus* OR "corona virus*" OR betacoronavirus* OR ncov* OR "n cov*" OR 2019nCoV* OR nCoV2019 OR "HCoV 19" OR HCoV19 OR Ncovor OR Ncorona* OR sars2* OR "sars 2*" OR sarscov* OR "sars cov*"))

AND

(TITLE_ABS:(selftest* OR "self test*" OR ((home* OR self* OR personal* OR Mail* OR screen* OR serial* OR mass*) AND (test* OR swab* OR detection) AND (antigen* OR nasal* OR nasopharyngeal* OR oropharyngeal* OR oral* OR saliva* OR "lateral flow*" OR LFA OR LFAs OR LFD OR LFDs OR RALFT OR RDT OR RDTs OR RATs OR RAT OR rapid* OR "point of care*")))

OR

KW:(selftest* OR "self test*" OR ((home* OR self* OR personal* OR Mail* OR screen* OR serial* OR mass*) AND (test* OR swab* OR detection) AND (antigen* OR nasal* OR nasopharyngeal* OR oropharyngeal* OR oral* OR saliva* OR "lateral flow*" OR LFA OR LFAs OR LFD OR LFDs OR RALFT OR RDT OR RDTs OR RATs OR RAT OR rapid* OR "point of care*"))))

AND

(PUBLISHER:MedRxiv)

AND

FIRST_PDATE:[2020-12-01 TO 2023-03-15]

WHO COVID-19 Database

<https://search.bvsalud.org/global-literature-on-novel-coronavirus-2019-ncov/advanced/?lang=en>

| **Trefferzahl** | **Datum** |
| --- | --- |
| 593 | 14.03.2023 |

P

| covid* OR  coronavirus* OR  "corona virus" OR  betacoronavirus* OR  ncov* OR  "n cov" OR  2019nCoV* OR  nCoV2019 OR  "HCoV 19" OR  HCoV19 OR  Ncovor OR  Ncorona* OR  sars2* OR  "sars 2" OR  sarscov* OR  "sars cov" |  |
| --- | --- |

I

| selftest OR  "self test" OR  ((home* OR  self* OR  personal* OR  mail* OR  screen* OR  serial* OR  mass*)  AND  (test* OR  swab* OR  detection)  AND  (antigen* OR  nasal* OR  nasopharyngeal* OR  oropharyngeal* OR  oral* OR  saliva* OR  "lateral flow" OR  LFA OR  LFAs OR  LFD OR  LFDs OR  RALFT OR  RDT OR  RDTs OR  RATs OR  RAT OR  rapid* OR  "point of care")) |  |
| --- | --- |

**String (title, abstract, keywords)**

**P (744979)**

(ti:(covid* OR coronavirus* OR "corona virus" OR betacoronavirus* OR ncov* OR "n cov" OR 2019nCoV* OR nCoV2019 OR "HCoV 19" OR HCoV19 OR Ncovor OR Ncorona* OR sars2* OR "sars 2" OR sarscov* OR "sars cov")

OR

ab:(covid* OR coronavirus* OR "corona virus" OR betacoronavirus* OR ncov* OR "n cov" OR 2019nCoV* OR nCoV2019 OR "HCoV 19" OR HCoV19 OR Ncovor OR Ncorona* OR sars2* OR "sars 2" OR sarscov* OR "sars cov")

OR

kw:(covid* OR coronavirus* OR "corona virus" OR betacoronavirus* OR ncov* OR "n cov" OR 2019nCoV* OR nCoV2019 OR "HCoV 19" OR HCoV19 OR Ncovor OR Ncorona* OR sars2* OR "sars 2" OR sarscov* OR "sars cov"))

**I (13259)**

(ti:(selftest OR "self test" OR ((home* OR self* OR personal* OR mail* OR screen* OR serial* OR mass*) AND (test* OR swab* OR detection) AND (antigen* OR nasal* OR nasopharyngeal* OR oropharyngeal* OR oral* OR saliva* OR "lateral flow" OR LFA OR LFAs OR LFD OR LFDs OR RALFT OR RDT OR RDTs OR RATs OR RAT OR rapid* OR "point of care")))

OR

ab:(selftest OR "self test" OR ((home* OR self* OR personal* OR mail* OR screen* OR serial* OR mass*) AND (test* OR swab* OR detection) AND (antigen* OR nasal* OR nasopharyngeal* OR oropharyngeal* OR oral* OR saliva* OR "lateral flow" OR LFA OR LFAs OR LFD OR LFDs OR RALFT OR RDT OR RDTs OR RATs OR RAT OR rapid* OR "point of care")))

OR

kw:(selftest OR "self test" OR ((home* OR self* OR personal* OR mail* OR screen* OR serial* OR mass*) AND (test* OR swab* OR detection) AND (antigen* OR nasal* OR nasopharyngeal* OR oropharyngeal* OR oral* OR saliva* OR "lateral flow" OR LFA OR LFAs OR LFD OR LFDs OR RALFT OR RDT OR RDTs OR RATs OR RAT OR rapid* OR "point of care"))))

**P AND I: 13015**

**Filter Databases: 891**

AND db:("PREPRINT-RESEARCHSQUARE" OR "PREPRINT-SSRN" OR "MDPI" OR "Academic Search Complete" OR "COVIDWHO" OR "PREPRINT-ARXIV" OR "GIM" OR "Centers for Disease Control and Prevention" OR "CAB Abstracts" OR "LILACS")

**Filter Publication Year: 593**

AND entry_date:([20201201 TO 20230309])

**Second search – March 1^st^ 2023 until August 1^st^ 2024**

Main concepts definition

P

| SARS-CoV-2 |  |
| --- | --- |

I

| self-testing |  |
| --- | --- |

Strategy

| 1 | P |  |
| --- | --- | --- |
| 2 | I |  |
| 3 | 1 AND 2 |  |

**Limit:** Publications from 01.03.2023-01.08.2024

Databases

- PubMed
- EMBASE
- Web of Science Core Collection
- medRxiv (via Europe PMC)
- WHO COVID-19 Database (no longer available)

Results report

The results were saved in Endnote and deduplicated. Some articles could still appear more than once.

The hits are sorted by database in Endnote. The PubMed hits were the first to be exported in Endnote. This makes them preferred for deduplication. In other words, in the case of duplicates, entries are removed from other databases.

The number of hits for each database in this report is based on its pre-deduplication status.

PubMed

| **Trefferzahl** | **Datum** |
| --- | --- |
| 1736 | 01.08.2024 |

P

| 1 | **"Coronavirus"[Mesh] OR**  **"COVID-19"[Mesh] OR**  covid*[tiab] OR  coronavirus*[tiab] OR  "corona virus*"[tiab] OR  betacoronavirus*[tiab] OR  ncov*[tiab] OR  "n cov*"[tiab] OR  2019nCoV*[tiab] OR  nCoV2019[tiab] OR  "HCoV 19"[tiab] OR  HCoV19[tiab] OR  Ncovor[tiab] OR  Ncorona*[tiab] OR  sars2*[tiab] OR  "sars 2*"[tiab] OR  sarscov*[tiab] OR  "sars cov*"[tiab] | 455171 |
| --- | --- | --- |

I

| 2 | **"Self-Testing"[MeSH] OR**  selftest*[tiab] OR  "self test*"[tiab] OR  ((home*[tiab] OR  self*[tiab] OR  personal*[tiab] OR  mail*[tiab] OR  screen*[tiab] OR  serial*[tiab] OR  mass*[tiab])  AND  **("COVID-19 Testing"[Mesh] OR**  test*[tiab] OR  swab*[tiab] OR  detection[tiab])  AND  **("Antigens"[MeSH Terms] OR**  antigen*[tiab] OR  nasal*[tiab] OR  nasopharyngeal*[tiab] OR  oropharyngeal*[tiab] OR  oral*[tiab] OR  saliva*[tiab] OR  "lateral flow*"[tiab] OR  LFA[tiab] OR  LFAs [tiab] OR  LFD [tiab] OR  LFDs [tiab] OR  RALFT[tiab] OR  RDT[tiab] OR  RDTs[tiab] OR  RATs[tiab] OR  RAT[tiab] OR  rapid*[tiab] OR  "point of care*"[tiab])) | 204712 |
| --- | --- | --- |

Strings

1-2 as in the tables above

| **Field** | **String** | **Hits** |
| --- | --- | --- |
| 3 | #1 AND #2 | 8768 |

**Publication year from 01.03.2023**

| 4 | #3 AND ("2023/03/01"[PDAT] : "2024/08/01"[PDAT]) | 1736 |
| --- | --- | --- |

Embase

| **Trefferzahl** | **Datum** |
| --- | --- |
| 695 | 01.08.2024 |

P

| 1 | **'Coronavirinae'/exp OR**  **'Coronavirus infection'/exp OR**  covid*:ti,ab,kw OR  coronavirus*:ti,ab,kw OR  "corona virus*":ti,ab,kw OR  betacoronavirus*:ti,ab,kw OR  ncov*:ti,ab,kw OR  "n cov*":ti,ab,kw OR  2019nCoV*:ti,ab,kw OR  nCoV2019:ti,ab,kw OR  "HCoV 19":ti,ab,kw OR  HCoV19:ti,ab,kw OR  Ncovor:ti,ab,kw OR  Ncorona*:ti,ab,kw OR  sars2*:ti,ab,kw OR  "sars 2*":ti,ab,kw OR  sarscov*:ti,ab,kw OR  "sars cov*":ti,ab,kw |  |
| --- | --- | --- |

I

| 2 | **'self-testing'/exp OR**  selftest*:ti,ab,kw OR  "self test*":ti,ab,kw OR  ((home*:ti,ab,kw OR  self*:ti,ab,kw OR  personal*:ti,ab,kw OR  mail*:ti,ab,kw OR  screen*:ti,ab,kw OR  serial*:ti,ab,kw OR  mass*:ti,ab,kw)  AND  **('COVID-19 testing'/exp OR**  test*:ti,ab,kw OR  swab*:ti,ab,kw OR  detection:ti,ab,kw)  AND  **('antigen'/exp OR**  "antigen*":ti,ab,kw OR  nasal*:ti,ab,kw OR  nasopharyngeal*:ti,ab,kw OR  oropharyngeal*:ti,ab,kw OR  oral*:ti,ab,kw OR  saliva*:ti,ab,kw OR  "lateral flow*":ti,ab,kw OR  LFA:ti,ab,kw OR  LFAs:ti,ab,kw OR  LFD:ti,ab,kw OR  LFDs:ti,ab,kw OR  RALFT:ti,ab,kw OR  RDT:ti,ab,kw OR  RDTs:ti,ab,kw OR  RATs:ti,ab,kw OR  RAT:ti,ab,kw OR  rapid*:ti,ab,kw OR  "point of care*":ti,ab,kw)) |  |
| --- | --- | --- |

Strings

| **Field** | **String** | **Hits** |
| --- | --- | --- |
| 1 | 'coronavirinae'/exp OR 'coronavirus infection'/exp OR covid*:ti,ab,kw OR coronavirus*:ti,ab,kw OR 'corona virus*':ti,ab,kw OR betacoronavirus*:ti,ab,kw OR ncov*:ti,ab,kw OR 'n cov*':ti,ab,kw OR 2019ncov*:ti,ab,kw OR ncov2019:ti,ab,kw OR 'hcov 19':ti,ab,kw OR hcov19:ti,ab,kw OR ncovor:ti,ab,kw OR ncorona*:ti,ab,kw OR sars2*:ti,ab,kw OR 'sars 2*':ti,ab,kw OR sarscov*:ti,ab,kw OR 'sars cov*':ti,ab,kw | 558507 |
| 2 | 'self-testing'/exp OR selftest*:ti,ab,kw OR 'self test*':ti,ab,kw OR ((home*:ti,ab,kw OR self*:ti,ab,kw OR personal*:ti,ab,kw OR mail*:ti,ab,kw OR screen*:ti,ab,kw OR serial*:ti,ab,kw OR mass*:ti,ab,kw) AND ('covid-19 testing'/exp OR test*:ti,ab,kw OR swab*:ti,ab,kw OR detection:ti,ab,kw) AND ('antigen'/exp OR 'antigen*':ti,ab,kw OR nasal*:ti,ab,kw OR nasopharyngeal*:ti,ab,kw OR oropharyngeal*:ti,ab,kw OR oral*:ti,ab,kw OR saliva*:ti,ab,kw OR 'lateral flow*':ti,ab,kw OR lfa:ti,ab,kw OR lfas:ti,ab,kw OR lfd:ti,ab,kw OR lfds:ti,ab,kw OR ralft:ti,ab,kw OR rdt:ti,ab,kw OR rdts:ti,ab,kw OR rats:ti,ab,kw OR rat:ti,ab,kw OR rapid*:ti,ab,kw OR 'point of care*':ti,ab,kw)) | 315359 |
| 3 | #1 AND #2 | 11728 |

**Publication year from 01.03.2023**

| 4 | #3 AND [01-03-2023]/sd NOT [01-08-2024]/sd | 3242 |
| --- | --- | --- |

**Embase filter to switch off PubMed**

| 5 | #4 NOT ([medline]/lim OR [pubmed-not-medline]/lim) | 1599 |
| --- | --- | --- |

**Embase filter to exclude document types not of interest**

| 6 | #5 NOT ('Conference Abstract'/it OR 'Note'/it OR 'chapter'/it) | 695 |
| --- | --- | --- |

Web of Science Core Collection

| **Trefferzahl** | **Datum** |
| --- | --- |
| 997 | 01.08.2024 |

P

| 1 | covid* OR  coronavirus* OR  "corona virus*" OR  betacoronavirus* OR  ncov* OR  "n cov*" OR  2019nCoV* OR  nCoV2019 OR  "HCoV 19" OR  HCoV19 OR  Ncovor OR  Ncorona* OR  sars2* OR  "sars 2*" OR  sarscov* OR  "sars cov*" |  |
| --- | --- | --- |

I

| 2 | selftest* OR  "self test*" OR  ((home* OR  self* OR  personal* OR  mail* OR  screen* OR  serial* OR  mass*)  AND  (test* OR  swab*” OR  detection”)  AND  (antigen* OR  nasal* OR  nasopharyngeal* OR  oropharyngeal* OR  oral* OR  saliva* OR  "lateral flow*" OR  LFA OR  LFAs OR  LFD OR  LFDs OR  RALFT OR  RDT OR  RDTs OR  RATs OR  RAT OR  rapid* OR  "point of care*")) |  |
| --- | --- | --- |

Strings

| **Field** | **String** | **Hits** |
| --- | --- | --- |
| 1 (TI,AB,AK) | TI=(covid* OR coronavirus* OR "corona virus*" OR betacoronavirus* OR ncov* OR "n cov*" OR 2019nCoV* OR nCoV2019 OR "HCoV 19" OR HCoV19 OR Ncovor OR Ncorona* OR sars2* OR "sars 2*" OR sarscov* OR "sars cov*") OR  AB=(covid* OR coronavirus* OR "corona virus*" OR betacoronavirus* OR ncov* OR "n cov*" OR 2019nCoV* OR nCoV2019 OR "HCoV 19" OR HCoV19 OR Ncovor OR Ncorona* OR sars2* OR "sars 2*" OR sarscov* OR "sars cov*") OR  AK=(covid* OR coronavirus* OR "corona virus*" OR betacoronavirus* OR ncov* OR "n cov*" OR 2019nCoV* OR nCoV2019 OR "HCoV 19" OR HCoV19 OR Ncovor OR Ncorona* OR sars2* OR "sars 2*" OR sarscov* OR "sars cov*") | 452075 |
| 2 (TI,AB,AK) | TI=(selftest* OR "self test*" OR ((home* OR self* OR personal* OR mail* OR screen* OR serial* OR mass*) AND (test* OR swab*” OR detection”) AND (antigen* OR nasal* OR nasopharyngeal* OR oropharyngeal* OR oral* OR saliva* OR "lateral flow*" OR LFA OR LFAs OR LFD OR LFDs OR RALFT OR RDT OR RDTs OR RATs OR RAT OR rapid* OR "point of care*"))) OR  AB=(selftest* OR "self test*" OR ((home* OR self* OR personal* OR mail* OR screen* OR serial* OR mass*) AND (test* OR swab*” OR detection”) AND (antigen* OR nasal* OR nasopharyngeal* OR oropharyngeal* OR oral* OR saliva* OR "lateral flow*" OR LFA OR LFAs OR LFD OR LFDs OR RALFT OR RDT OR RDTs OR RATs OR RAT OR rapid* OR "point of care*"))) OR  AK=(selftest* OR "self test*" OR ((home* OR self* OR personal* OR mail* OR screen* OR serial* OR mass*) AND (test* OR swab*” OR detection”) AND (antigen* OR nasal* OR nasopharyngeal* OR oropharyngeal* OR oral* OR saliva* OR "lateral flow*" OR LFA OR LFAs OR LFD OR LFDs OR RALFT OR RDT OR RDTs OR RATs OR RAT OR rapid* OR "point of care*"))) | 133606 |

| 3 | #1 AND #2 | 5308 |
| --- | --- | --- |

**Publication year from 01.03.2023**

| 4 | From 2023-03-01 to 2024-08-01 | 997 |
| --- | --- | --- |

MedRxiv (via Europe PMC)

<https://europepmc.org/>

| **Records number** | **Date** |
| --- | --- |
| 114 | 01.08.2024 |

P

| 1 | covid* OR  coronavirus* OR  "corona virus*" OR  betacoronavirus* OR  ncov* OR  "n cov*" OR  2019nCoV* OR  nCoV2019 OR  "HCoV 19" OR  HCoV19 OR  Ncovor OR  Ncorona* OR  sars2* OR  "sars 2*" OR  sarscov* OR  "sars cov*" |  |
| --- | --- | --- |

I

| 2 | selftest* OR  "self test*" OR  ((home* OR  self* OR  personal* OR  mail* OR  screen* OR  serial* OR  mass*)  AND  (test* OR  swab* OR  detection)  AND  (antigen* OR  nasal* OR  nasopharyngeal* OR  oropharyngeal* OR  oral* OR  saliva* OR  "lateral flow*" OR  LFA OR  LFAs OR  LFD OR  LFDs OR  RALFT OR  RDT OR  RDTs OR  RATs OR  RAT OR  rapid* OR  "point of care*")) |  |
| --- | --- | --- |

Strings

| **Field** | **String** | **Hits** |
| --- | --- | --- |
| 1 | (TITLE_ABS:(covid* OR coronavirus* OR "corona virus*" OR betacoronavirus* OR ncov* OR "n cov*" OR 2019nCoV* OR nCoV2019 OR "HCoV 19" OR HCoV19 OR Ncovor OR Ncorona* OR sars2* OR "sars 2*" OR sarscov* OR "sars cov*")  OR  KW:(covid* OR coronavirus* OR "corona virus*" OR betacoronavirus* OR ncov* OR "n cov*" OR 2019nCoV* OR nCoV2019 OR "HCoV 19" OR HCoV19 OR Ncovor OR Ncorona* OR sars2* OR "sars 2*" OR sarscov* OR "sars cov*")) | 911407 |
| 2 | AND  (TITLE_ABS:(selftest* OR "self test*" OR ((home* OR self* OR personal* OR Mail* OR screen* OR serial* OR mass*) AND (test* OR swab* OR detection) AND (antigen* OR nasal* OR nasopharyngeal* OR oropharyngeal* OR oral* OR saliva* OR "lateral flow*" OR LFA OR LFAs OR LFD OR LFDs OR RALFT OR RDT OR RDTs OR RATs OR RAT OR rapid* OR "point of care*")))  OR  KW:(selftest* OR "self test*" OR ((home* OR self* OR personal* OR Mail* OR screen* OR serial* OR mass*) AND (test* OR swab* OR detection) AND (antigen* OR nasal* OR nasopharyngeal* OR oropharyngeal* OR oral* OR saliva* OR "lateral flow*" OR LFA OR LFAs OR LFD OR LFDs OR RALFT OR RDT OR RDTs OR RATs OR RAT OR rapid* OR "point of care*")))) | 4018097 |
| 3 | AND  (PUBLISHER:MedRxiv) | 1395 |
| 4 | AND  FIRST_PDATE:[2023-03-01 TO 2024-08-01] | 114 |

WHO COVID-19 Database (up to date until 06/2023, taken offline in 01/2024)

<https://www.who.int/emergencies/diseases/novel-coronavirus-2019/global-research-on-novel-coronavirus-2019-ncov>

**Third search – August 1^st^ 2024 until October 1^st^ 2025**

Main concepts definition

P

| SARS-CoV-2 |  |
| --- | --- |

I

| self-testing |  |
| --- | --- |

Strategy

| 1 | P |  |
| --- | --- | --- |
| 2 | I |  |
| 3 | 1 AND 2 |  |

**Limit:** Publications from 01.08.2024-01.10.2025 (Cochrane 01.12.2020-01.10.2025)

Databases

- PubMed
- Cochrane Library
- EMBASE
- Web of Science Core Collection
- Clinical Trials.gov
- medRxiv (via Europe PMC)
- WHO COVID-19 Database not searched (updated until june 2023 no longer available from 2024)

Results report

The results were saved in Endnote and deduplicated. Some articles could still appear more than once.

The hits are sorted by database in Endnote. The PubMed hits were the first to be exported in Endnote. This makes them preferred for deduplication. In other words, in the case of duplicates, entries are removed from other databases.

The number of hits for each database in this report is based on its pre-deduplication status.

PubMed

| **Trefferzahl** | **Datum** |
| --- | --- |
| 957 | 01.10.2025 |

P

| 1 | **"Coronavirus"[Mesh] OR**  **"COVID-19"[Mesh] OR**  covid*[tiab] OR  coronavirus*[tiab] OR  "corona virus*"[tiab] OR  betacoronavirus*[tiab] OR  ncov*[tiab] OR  "n cov*"[tiab] OR  2019nCoV*[tiab] OR  nCoV2019[tiab] OR  "HCoV 19"[tiab] OR  HCoV19[tiab] OR  Ncovor[tiab] OR  Ncorona*[tiab] OR  sars2*[tiab] OR  "sars 2*"[tiab] OR  sarscov*[tiab] OR  "sars cov*"[tiab] | 507256 |
| --- | --- | --- |

I

| 2 | **"Self-Testing"[MeSH] OR**  selftest*[tiab] OR  "self test*"[tiab] OR  ((home*[tiab] OR  self*[tiab] OR  personal*[tiab] OR  mail*[tiab] OR  screen*[tiab] OR  serial*[tiab] OR  mass*[tiab])  AND  **("COVID-19 Testing"[Mesh] OR**  test*[tiab] OR  swab*[tiab] OR  detection[tiab])  AND  **("Antigens"[MeSH] OR**  antigen*[tiab] OR  nasal*[tiab] OR  nasopharyngeal*[tiab] OR  oropharyngeal*[tiab] OR  oral*[tiab] OR  saliva*[tiab] OR  "lateral flow*"[tiab] OR  LFA[tiab] OR  LFAs [tiab] OR  LFD [tiab] OR  LFDs [tiab] OR  RALFT[tiab] OR  RDT[tiab] OR  RDTs[tiab] OR  RATs[tiab] OR  RAT[tiab] OR  rapid*[tiab] OR  "point of care*"[tiab])) | 221508 |
| --- | --- | --- |

Strings

1-2 as in the tables above

| **Field** | **String** | **Hits** |
| --- | --- | --- |
| 3 | #1 AND #2 | 9693 |

**Publication year from August 1^st^ 2024**

| 4 | #3 AND ("2024/08/01"[PDAT] : "2025/10/01"[PDAT]) | 957 |
| --- | --- | --- |

Cochrane Library

| **Hits** | **Date** |
| --- | --- |
| 830 | 01.10.2025 |

P

| 1 | **[mh "Coronavirus"] OR**  **[mh "COVID-19"] OR**  covid*:ti,ab,kw OR  coronavirus*:ti,ab,kw OR  corona NEXT/2 virus*:ti,ab,kw OR  betacoronavirus*:ti,ab,kw OR  ncov*:ti,ab,kw OR  n NEXT/2 cov*:ti,ab,kw OR  2019nCoV*:ti,ab,kw OR  nCoV2019:ti,ab,kw OR  "HCoV 19":ti,ab,kw OR  HCoV19:ti,ab,kw OR  Ncovor:ti,ab,kw OR  Ncorona*:ti,ab,kw OR  sars2*:ti,ab,kw OR  sars NEXT/2 2*:ti,ab,kw OR  sarscov*:ti,ab,kw OR  sars NEXT/2 cov*:ti,ab,kw | 25359 |
| --- | --- | --- |

I

| 2 | **[mh "Self-Testing"] OR**  selftest*:ti,ab,kw OR  self NEXT/2 test*:ti,ab,kw OR  ((home*:ti,ab,kw OR  self*:ti,ab,kw OR  personal*:ti,ab,kw OR  mail*:ti,ab,kw OR  screen*:ti,ab,kw OR  serial*:ti,ab,kw OR  mass*:ti,ab,kw)  AND  **([mh "COVID-19 Testing"] OR**  test*:ti,ab,kw OR  swab*:ti,ab,kw OR  detection:ti,ab,kw)  AND  **([mh "Antigens"] OR**  antigen*:ti,ab,kw OR  nasal*:ti,ab,kw OR  nasopharyngeal*:ti,ab,kw OR  oropharyngeal*:ti,ab,kw OR  oral*:ti,ab,kw OR  saliva*:ti,ab,kw OR  lateral NEXT/2 flow*:ti,ab,kw OR  LFA:ti,ab,kw OR  LFAs:ti,ab,kw OR  LFD:ti,ab,kw OR  LFDs:ti,ab,kw OR  RALFT:ti,ab,kw OR  RDT:ti,ab,kw OR  RDTs:ti,ab,kw OR  RATs:ti,ab,kw OR  RAT:ti,ab,kw OR  rapid*:ti,ab,kw OR  point NEXT/2 of NEXT/2 care*:ti,ab,kw)) | 37129 |
| --- | --- | --- |

Strings

1-2 as in the tables above

| **Field** | **String** | **Hits** |
| --- | --- | --- |
| 3 | #1 AND #2 | 1110 |

**Publication year from December 1^st^ 2020**

| 4 | publication date from 01.12.2020 to present | 830 |
| --- | --- | --- |

Embase

| **Trefferzahl** | **Datum** |
| --- | --- |
| 1575 | 01.10.2025 |

P

| 1 | **'Coronavirinae'/exp OR**  **'Coronavirus infection'/exp OR**  covid*:ti,ab,kw OR  coronavirus*:ti,ab,kw OR  "corona virus*":ti,ab,kw OR  betacoronavirus*:ti,ab,kw OR  ncov*:ti,ab,kw OR  "n cov*":ti,ab,kw OR  2019nCoV*:ti,ab,kw OR  nCoV2019:ti,ab,kw OR  "HCoV 19":ti,ab,kw OR  HCoV19:ti,ab,kw OR  Ncovor:ti,ab,kw OR  Ncorona*:ti,ab,kw OR  sars2*:ti,ab,kw OR  "sars 2*":ti,ab,kw OR  sarscov*:ti,ab,kw OR  "sars cov*":ti,ab,kw |  |
| --- | --- | --- |

I

| 2 | **'self-testing'/exp OR**  selftest*:ti,ab,kw OR  "self test*":ti,ab,kw OR  ((home*:ti,ab,kw OR  self*:ti,ab,kw OR  personal*:ti,ab,kw OR  mail*:ti,ab,kw OR  screen*:ti,ab,kw OR  serial*:ti,ab,kw OR  mass*:ti,ab,kw)  AND  **('COVID-19 testing'/exp OR**  test*:ti,ab,kw OR  swab*:ti,ab,kw OR  detection:ti,ab,kw)  AND  **('antigen'/exp OR**  "antigen*":ti,ab,kw OR  nasal*:ti,ab,kw OR  nasopharyngeal*:ti,ab,kw OR  oropharyngeal*:ti,ab,kw OR  oral*:ti,ab,kw OR  saliva*:ti,ab,kw OR  "lateral flow*":ti,ab,kw OR  LFA:ti,ab,kw OR  LFAs:ti,ab,kw OR  LFD:ti,ab,kw OR  LFDs:ti,ab,kw OR  RALFT:ti,ab,kw OR  RDT:ti,ab,kw OR  RDTs:ti,ab,kw OR  RATs:ti,ab,kw OR  RAT:ti,ab,kw OR  rapid*:ti,ab,kw OR  "point of care*":ti,ab,kw)) |  |
| --- | --- | --- |

Strings

| **Field** | **String** | **Hits** |
| --- | --- | --- |
| 1 | 'coronavirinae'/exp OR 'coronavirus infection'/exp OR covid*:ti,ab,kw OR coronavirus*:ti,ab,kw OR 'corona virus*':ti,ab,kw OR betacoronavirus*:ti,ab,kw OR ncov*:ti,ab,kw OR 'n cov*':ti,ab,kw OR 2019ncov*:ti,ab,kw OR ncov2019:ti,ab,kw OR 'hcov 19':ti,ab,kw OR hcov19:ti,ab,kw OR ncovor:ti,ab,kw OR ncorona*:ti,ab,kw OR sars2*:ti,ab,kw OR 'sars 2*':ti,ab,kw OR sarscov*:ti,ab,kw OR 'sars cov*':ti,ab,kw | 644620 |
| 2 | 'self-testing'/exp OR selftest*:ti,ab,kw OR 'self test*':ti,ab,kw OR ((home*:ti,ab,kw OR self*:ti,ab,kw OR personal*:ti,ab,kw OR mail*:ti,ab,kw OR screen*:ti,ab,kw OR serial*:ti,ab,kw OR mass*:ti,ab,kw) AND ('covid-19 testing'/exp OR test*:ti,ab,kw OR swab*:ti,ab,kw OR detection:ti,ab,kw) AND ('antigen'/exp OR 'antigen*':ti,ab,kw OR nasal*:ti,ab,kw OR nasopharyngeal*:ti,ab,kw OR oropharyngeal*:ti,ab,kw OR oral*:ti,ab,kw OR saliva*:ti,ab,kw OR 'lateral flow*':ti,ab,kw OR lfa:ti,ab,kw OR lfas:ti,ab,kw OR lfd:ti,ab,kw OR lfds:ti,ab,kw OR ralft:ti,ab,kw OR rdt:ti,ab,kw OR rdts:ti,ab,kw OR rats:ti,ab,kw OR rat:ti,ab,kw OR rapid*:ti,ab,kw OR 'point of care*':ti,ab,kw)) | 359395 |
| 3 | #1 AND #2 | 14386 |

**Publication year from August 1^st^ 2024**

| 4 | #3 AND [01-08-2024]/sd NOT [01-10-2025]/sd | 2768 |
| --- | --- | --- |

**Embase filter to switch off PubMed**

| 5 | #4 NOT ([medline]/lim OR [pubmed-not-medline]/lim) | 1987 |
| --- | --- | --- |

**Embase filter to exclude document types not of interest**

| 6 | #5 NOT ('Conference Abstract'/it OR 'Note'/it OR 'chapter'/it) | 1575 |
| --- | --- | --- |

Web of Science Core Collection

| **Trefferzahl** | **Datum** |
| --- | --- |
| 573 | 01.10.2025 |

P

| 1 | covid* OR  coronavirus* OR  "corona virus*" OR  betacoronavirus* OR  ncov* OR  "n cov*" OR  2019nCoV* OR  nCoV2019 OR  "HCoV 19" OR  HCoV19 OR  Ncovor OR  Ncorona* OR  sars2* OR  "sars 2*" OR  sarscov* OR  "sars cov*" |  |
| --- | --- | --- |

I

| 2 | selftest* OR  "self test*" OR  ((home* OR  self* OR  personal* OR  mail* OR  screen* OR  serial* OR  mass*)  AND  (test* OR  swab* OR  detection)  AND  (antigen* OR  nasal* OR  nasopharyngeal* OR  oropharyngeal* OR  oral* OR  saliva* OR  "lateral flow*" OR  LFA OR  LFAs OR  LFD OR  LFDs OR  RALFT OR  RDT OR  RDTs OR  RATs OR  RAT OR  rapid* OR  "point of care*")) |  |
| --- | --- | --- |

Strings

| **Field** | **String** | **Hits** |
| --- | --- | --- |
| 1 (TI,AB,AK) | TI=(covid* OR coronavirus* OR "corona virus*" OR betacoronavirus* OR ncov* OR "n cov*" OR 2019nCoV* OR nCoV2019 OR "HCoV 19" OR HCoV19 OR Ncovor OR Ncorona* OR sars2* OR "sars 2*" OR sarscov* OR "sars cov*") OR  AB=(covid* OR coronavirus* OR "corona virus*" OR betacoronavirus* OR ncov* OR "n cov*" OR 2019nCoV* OR nCoV2019 OR "HCoV 19" OR HCoV19 OR Ncovor OR Ncorona* OR sars2* OR "sars 2*" OR sarscov* OR "sars cov*") OR  AK=(covid* OR coronavirus* OR "corona virus*" OR betacoronavirus* OR ncov* OR "n cov*" OR 2019nCoV* OR nCoV2019 OR "HCoV 19" OR HCoV19 OR Ncovor OR Ncorona* OR sars2* OR "sars 2*" OR sarscov* OR "sars cov*") | 514778 |
| 2 (TI,AB,AK) | TI=(selftest* OR "self test*" OR ((home* OR self* OR personal* OR mail* OR screen* OR serial* OR mass*) AND (test* OR swab*” OR detection”) AND (antigen* OR nasal* OR nasopharyngeal* OR oropharyngeal* OR oral* OR saliva* OR "lateral flow*" OR LFA OR LFAs OR LFD OR LFDs OR RALFT OR RDT OR RDTs OR RATs OR RAT OR rapid* OR "point of care*"))) OR  AB=(selftest* OR "self test*" OR ((home* OR self* OR personal* OR mail* OR screen* OR serial* OR mass*) AND (test* OR swab*” OR detection”) AND (antigen* OR nasal* OR nasopharyngeal* OR oropharyngeal* OR oral* OR saliva* OR "lateral flow*" OR LFA OR LFAs OR LFD OR LFDs OR RALFT OR RDT OR RDTs OR RATs OR RAT OR rapid* OR "point of care*"))) OR  AK=(selftest* OR "self test*" OR ((home* OR self* OR personal* OR mail* OR screen* OR serial* OR mass*) AND (test* OR swab*” OR detection”) AND (antigen* OR nasal* OR nasopharyngeal* OR oropharyngeal* OR oral* OR saliva* OR "lateral flow*" OR LFA OR LFAs OR LFD OR LFDs OR RALFT OR RDT OR RDTs OR RATs OR RAT OR rapid* OR "point of care*"))) | 144422 |

| 3 | #1 AND #2 | 5891 |
| --- | --- | --- |

**Publication year from August 1^st^ 2024**

| 4 | From 2024-08-01 to 2025-10-01 | 573 |
| --- | --- | --- |

ClinicalTrial.gov

<http://www.clinicaltrials.gov/>

| **Hits** | **Date** |
| --- | --- |
| 952 | 01.10.2025 |

P

| 1 | covid OR  coronavirus OR  "corona virus" OR  betacoronavirus OR  ncov OR  "n cov" OR  2019nCoV OR  nCoV2019 OR  "HCoV 19" OR  HCoV19 OR  Ncovor OR  Ncorona OR  sars2 OR  "sars 2" OR  sarscov OR  "sars cov" |  |
| --- | --- | --- |

I

| 2 | selftest OR  "self test" OR  ((home OR  self OR  personal OR  mail OR  screen OR  serial OR  mass)  AND  (test OR  swab OR  detection)  AND  (antigen OR  nasal OR  nasopharyngeal OR  oropharyngeal OR  oral OR  saliva OR  "lateral flow" OR  LFA OR  LFAs OR  LFD OR  LFDs OR  RALFT OR  RDT OR  RDTs OR  RATs OR  RAT OR  rapid OR  "point of care")) |  |
| --- | --- | --- |

Strings

| **Field** | **String** | **Hits** |
| --- | --- | --- |
| 1 | (  covid OR coronavirus OR "corona virus" OR betacoronavirus OR ncov OR "n cov" OR 2019nCoV OR nCoV2019 OR "HCoV 19" OR HCoV19 OR Ncovor OR Ncorona OR sars2 OR "sars 2" OR sarscov OR "sars cov"  ) | 10439 |
| 2 | AND  (  selftest OR "self test" OR ((home OR self OR personal OR mail OR screen OR serial OR mass) AND (test OR swab OR detection) AND (antigen OR nasal OR nasopharyngeal OR oropharyngeal OR oral OR saliva OR "lateral flow" OR LFA OR LFAs OR LFD OR LFDs OR RALFT OR RDT OR RDTs OR RATs OR RAT OR rapid OR "point of care"))  ) | 21333 |

| 3 | 1 AND 2 | 952 |
| --- | --- | --- |

MedRxiv (via Europe PMC)

<https://europepmc.org/>

| **Records number** | **Date** |
| --- | --- |
| 41 | 01.10.2025 |

P

| 1 | covid* OR  coronavirus* OR  "corona virus*" OR  betacoronavirus* OR  ncov* OR  "n cov*" OR  2019nCoV* OR  nCoV2019 OR  "HCoV 19" OR  HCoV19 OR  Ncovor OR  Ncorona* OR  sars2* OR  "sars 2*" OR  sarscov* OR  "sars cov*" |  |
| --- | --- | --- |

I

| 2 | selftest* OR  "self test*" OR  ((home* OR  self* OR  personal* OR  mail* OR  screen* OR  serial* OR  mass*)  AND  (test* OR  swab* OR  detection)  AND  (antigen* OR  nasal* OR  nasopharyngeal* OR  oropharyngeal* OR  oral* OR  saliva* OR  "lateral flow*" OR  LFA OR  LFAs OR  LFD OR  LFDs OR  RALFT OR  RDT OR  RDTs OR  RATs OR  RAT OR  rapid* OR  "point of care*")) |  |
| --- | --- | --- |

Strings

| **Field** | **String** | **Hits** |
| --- | --- | --- |
| 1 | (TITLE_ABS:(covid* OR coronavirus* OR "corona virus*" OR betacoronavirus* OR ncov* OR "n cov*" OR 2019nCoV* OR nCoV2019 OR "HCoV 19" OR HCoV19 OR Ncovor OR Ncorona* OR sars2* OR "sars 2*" OR sarscov* OR "sars cov*")  OR  KW:(covid* OR coronavirus* OR "corona virus*" OR betacoronavirus* OR ncov* OR "n cov*" OR 2019nCoV* OR nCoV2019 OR "HCoV 19" OR HCoV19 OR Ncovor OR Ncorona* OR sars2* OR "sars 2*" OR sarscov* OR "sars cov*")) | 1118502 |
| 2 | AND  (TITLE_ABS:(selftest* OR "self test*" OR ((home* OR self* OR personal* OR Mail* OR screen* OR serial* OR mass*) AND (test* OR swab* OR detection) AND (antigen* OR nasal* OR nasopharyngeal* OR oropharyngeal* OR oral* OR saliva* OR "lateral flow*" OR LFA OR LFAs OR LFD OR LFDs OR RALFT OR RDT OR RDTs OR RATs OR RAT OR rapid* OR "point of care*")))  OR  KW:(selftest* OR "self test*" OR ((home* OR self* OR personal* OR Mail* OR screen* OR serial* OR mass*) AND (test* OR swab* OR detection) AND (antigen* OR nasal* OR nasopharyngeal* OR oropharyngeal* OR oral* OR saliva* OR "lateral flow*" OR LFA OR LFAs OR LFD OR LFDs OR RALFT OR RDT OR RDTs OR RATs OR RAT OR rapid* OR "point of care*")))) | 5116155 |
| 3 | AND  (PUBLISHER:MedRxiv) | 1437 |
| 4 | AND  FIRST_PDATE:[2024-08-01 TO 2025-10-01] | 41 |

# Text S4 – Methods: interpretation guide to the JBI tool for quasi-experimental studies

To evaluate the quality of the studies included in our analysis, we utilized the Joanna Briggs Institute (JBI) Critical Appraisal Tool for Quasi-Experimental Studies, a well-recognized instrument applicable to diverse study designs, particularly observational and non-randomized studies [16]. To ensure methodological rigor and reproducibility, we developed tailored interpretation guides aligned with key outcome domains including diagnostic performance, result verification, and implementation processes, allowing us to account for outcome-specific biases, confounders, and methodological variability

**JBI critical appraisal tool for quasi-experimental studies interpretation guide – Outcomes related to test positivity** (proportion of C19ST results positive, proportion of individuals receiving a positive C19ST result, proportion of invalid test results)

1. *Is it clear in the study what is the ‘cause’ and what is the ‘effect’ (i.e., there is no confusion about which variable comes first)?*
   - We answered yes if relevant outcomes were measured after self-testing was completed
   - We judged no if relevant outcomes were measured before self-testing was completed or while it was conducted
     1. For example, studies of only known covid cases who underwent RAT would be no (other data may be extracted from study for other meta-analyzable outcomes)
   - We judged unclear if we could not tell the order of self-testing and measurement of outcomes
2. *Were the participants included in any comparisons similar?*
   - We judged yes if (1) the study authors considered potential confounding factors and (2) respective characteristics of participants included in any comparisons were similar
   - We judged no if the study authors did not consider potential confounding factors or respective characteristics of participants included in any comparisons showed significant differences
   - We judged unclear if we could not sufficiently evaluate relevant characteristics of participants included in any comparisons or the only comparison was between self-testing and a prior situation without dedicated testing
     1. Unclear for studies where control is considered just “pre-implementation” and was not directly measured by study investigators (this is most studies, unless they specified clear comparator group such as gargle PCR for example)
3. *Were the participants included in any comparisons receiving similar treatment/care, other than the exposure or intervention of interest?*
   - We judged yes if the participants included in any comparisons received similar SARS-CoV-2 control measures other than self-testing
   - We judged no if the participants included in any comparisons received different SARS-CoV-2 control measures other than self-testing
   - We judged unclear if we could not sufficiently evaluate the SARS-CoV-2 control measures other than self-testing received by participants included in any comparisons or the only comparison was between self-testing and a prior situation without dedicated testing
     1. Unclear for studies where control is considered just “pre-implementation” and was not directly measured by study investigators (this is most studies, unless they specified clear comparator group such as gargle PCR for example)
4. *Was there a control group?*
   - Given that we consider every study as a before-after comparison, with the before-setting representing the control group, we judged yes for all studies
5. *Were there multiple measurements of the outcome both pre and post the intervention/exposure?*
   - Yes, if in the implementation study it was a multiple-testing regime
   - No, if the study used a single-testing regime
   - Unclear, if not clear how often participants tested or if individuals tested multiple times without a pre-specified testing regime
6. *Was follow-up complete, and if not, was follow-up adequately reported and strategies to deal with loss to follow-up employed?*
   - We judged yes if the study reported results on all participants who adhered the testing scheme (exclusive of lost to follow-up due to non-reporting)
   - We judged no if studies did not report on lost to follow-up or non-adherence
   - We judged unclear if studies used public health system extracted information
7. *Were the outcomes of participants included in any comparisons measured in the same way?*
   - We judged yes if
     1. antigen-based test was specified along with testing method (e.g., nasopharyngeal swab vs. anterior nares) and
     2. participants were actually tested at same frequency
   - We judged no if outcomes of participants included in any comparisons were measured differently, i.e.,
     1. if different antigen-based tests or methods used or
     2. there was or could have been different testing frequencies
   - We judged unclear if the type of antigen-based test was not specified nor testing frequency (e.g., public health extracted data)
8. *Were outcomes measured in a reliable way?*
   - We judged yes if
     1. all C19ST results were directly observed by study personnel or
     2. study participants had to hand in pictures of the C19ST results
   - We judged no if test results were reported by voice, email or similar without specific proof (e.g., requiring to upload a picture) of the test result
   - We judged unclear if the mode of test result reporting was not further specified
9. *Was appropriate statistical analysis used?*
   - We judged yes for studies where results are just count-related
   - We judged unclear if
     1. studies were based on statistical modeling or
     2. studies were based on assumptions that could not be verified or
     3. the results shown in the respective studies were incomplete or incorrect

**JBI critical appraisal tool for quasi-experimental studies interpretation guide – Outcomes related to confirmatory testing** (proportion of false-positive test results, proportion of COVID-19 cases missed)

1. *Is it clear in the study what is the ‘cause’ and what is the ‘effect’ (i.e., there is no confusion about which variable comes first)?*
   - We answered yes if relevant outcomes were measured for the self-testing procedure assessed
   - We judged no if relevant outcomes were measured for any other screening intervention than for the self-testing assessed
2. *Were the participants included in any comparisons similar?*
   - We judged yes if the study authors considered potential confounding factors and respective characteristics of participants included in any comparisons were similar
   - We judged no if the study authors did not consider potential confounding factors or respective characteristics of participants included in any comparisons showed significant differences
3. *Were the participants included in any comparisons receiving similar treatment/care, other than the exposure or intervention of interest?*
   - We judged yes if the participants included in any comparisons received similar SARS-CoV-2 control measures other than when they self-tested
   - We judged no if the participants included in any comparisons received different SARS-CoV-2 control measures other than when they self-tested
4. *Was there a control group?*
   - Given that we consider every study as a before-after comparison, with the before-setting representing the control group, we judged yes for all studies
5. *Were there multiple measurements of the outcome both pre and post the intervention/exposure?*
   - Not applicable
6. *Was follow-up complete, and if not, was follow-up adequately reported and strategies to deal with loss to follow-up employed?*For “missed cases”:
   - We judged yes if all individuals performing self-testing received confirmatory testing
   - We judged no if only part of the individuals performing self-testing received confirmatory testing
   - We judged unclear if we could not tell the proportion of individuals performing self-testing received confirmatory testing

For “false-positive proportion”:

- - We judged yes if all C19ST received confirmatory testing
  - We judged no if all C19ST received confirmatory testing
  - We judged unclear if all C19ST received confirmatory testing

1. *Were the outcomes of participants included in any comparisons measured in the same way?*
   - We judged yes if confirmatory testing was all done using the same method (e.g., all RT-PCR, but doesn’t have to be the same RT-PCR method)
   - We judged no if confirmatory testing was done using different methods
   - We judged unclear if we could not tell the method of confirmatory testing
2. *Were outcomes measured in a reliable way?*

For “missed cases”:

- - We judged yes if all individuals performing self-testing received confirmatory testing
  - We judged no if only part of the individuals performing self-testing received confirmatory testing
  - We judged unclear if we could not tell the proportion of individuals performing self-testing received confirmatory testing

For “false-positive proportion”:

- - We judged yes if all C19ST received confirmatory testing
  - We judged no if all C19ST received confirmatory testing
  - We judged unclear if all C19ST received confirmatory testing

1. *Were appropriate confirmatory used?*
   - We judged yes if confirmatory testing was performed using RT-PCR
   - We judged no if confirmatory testing was performed on any method other than RT-PCR
   - We judged unclear if we could not tell the method of confirmatory testing

**JBI critical appraisal tool for quasi-experimental studies interpretation guide → Outcomes related to operational indices** (proportion of individuals willing to participate in C19St [“uptake”], proportion of individuals reporting test results to study / public health authorities and proportion of test reported out of all tests required to be conducted [“reporting” and “adherence”])

1. *Is it clear in the study what is the ‘cause’ and what is the ‘effect’ (i.e., there is no confusion about which variable comes first)?*
   - We judged ‘yes’ if testing and / or result reporting was voluntarily
   - We judged ‘no’ if testing and / or result reporting was compulsory
2. *Were the participants included in any comparisons similar?*
   - We judged yes if the study authors considered potential confounding factors and respective characteristics of participants included in any comparisons were similar
   - We judged no if the study authors did not consider potential confounding factors or respective characteristics of participants included in any comparisons showed significant differences
   - We judged unclear if we could not sufficiently evaluate relevant characteristics of participants included in any comparisons or the only comparison was between self-testing and a prior situation without dedicated testing
     1. Unclear for studies where control is considered just “pre-implementation” and was not directly measured by study investigators (this is most studies, unless they specified clear comparator group such as gargle PCR for example)
3. *Were the participants included in any comparisons receiving similar treatment/care, other than the exposure or intervention of interest?*
   - We judged yes if the participants included in any comparisons received similar SARS-CoV-2 control measures other than self-testing
   - We judged no if the participants included in any comparisons received different SARS-CoV-2 control measures other than self-testing
   - We judged unclear if we could not sufficiently evaluate the SARS-CoV-2 control measures other than self-testing received by participants included in any comparisons or the only comparison was between self-testing and a prior situation without dedicated testing
     1. Unclear for studies where control is considered just “pre-implementation” and was not directly measured by study investigators (this is most studies, unless they specified clear comparator group such as gargle PCR for example)
4. *Was there a control group?*
   - Given that we consider every study as a before-after comparison, with the before-setting representing the control group, we judged yes for all studies
5. *Were there multiple measurements of the outcome both pre and post the intervention/exposure?*
   - Not applicable
6. *Was follow-up complete, and if not, was follow-up adequately reported and strategies to deal with loss to follow-up employed?*For “uptake”,
   - we judged yes if number eligible is reported and registered number is described
   - we judged no if total number eligible for self-testing is not reported

For “reporting” / “adherence”,

- - we judged ‘yes’ if
    1. C19ST occurred only once or
    2. the study describes the number of tests reported and not-reported (i.e., these are not lost to follow-up) under multiple-test regimes
  - we judged no if we could not determine how many people did not self-report under a routine testing scheme

1. *Were the outcomes of participants included in any comparisons measured in the same way?*
   - N/A
2. *Were outcomes measured in a reliable way?*For “uptake”,
   - We judged yes if proof was available that people were actually willing to test (e.g., they were willing to receive testing kits from the study personnel)
   - We judged no if people only reported by voice, email or similar that they tested, without specific proof (e.g., requiring to upload a picture of the tests received)
   - We judged unclear if we could not tell by which means the number of people that tested was evaluated

For “reporting” / “adherence”,

- - We judged yes if
    1. all C19ST results were directly observed by study personnel or
    2. study participants had to hand in pictures of the C19ST results
  - We judged no if test results were reported by voice, email or similar without specific proof (e.g., requiring to upload a picture) of the test result
  - We judged unclear if the mode of test result reporting was not further specified

1. *Was appropriate statistical analysis used?*
   - We judged yes for studies where results are just count-related
   - We judged unclear if
     1. studies were based on statistical modeling or
     2. studies were based on assumptions that could not be verified or
     3. the results shown in the respective studies were incomplete or incorrect

# Text S5 – Methods: details concerning the use of a binomial-normal generalized linear mixed model for meta-analysis

We conducted a sensitivity analysis to compare our results using a binomial-normal generalized linear mixed model (GLMM) for meta-analysis [18] with those from a conventional inverse-variance weighted meta-analysis of transformed proportions. Specifically, we used a standard logit transformation and added a value of 1 to both the numerator and denominator when the proportion was 0. We used the “rma.uni” function in the “metafor” R package to conduct these analyses [23]. We ran the analyses for the eight primary outcomes: proportion of invalid tests, missed cases, false positives, tests positives, new cases, adherent, test reporting, and test update.

The results of the sensitivity analyses are summarized in the Figure and Table below. For most of the outcomes, the results were nearly identical between the two approaches. There were, however, clear differences in the point estimates for the missed cases outcome (14.0% versus 27.4%). This is likely due to the fact that both methods had a high degree of uncertainty in the pooled estimate (e.g., wide confidence intervals). The conclusions of the analyses were unchanged when using the inverse-variance weighted meta-analytic method.

**Figure.** Sensitivity analyses comparing the meta-analysis pooled estimates from the generalized linear mixed model to an inverse-variance weighted meta-analysis.

*Caption:* The pooled estimates from the general linear mixed model are reflected through the orange dots (point estimate) and lines (95% confidence interval) and those from the inverse-variance weighted meta-analysis through the green dots (point estimate) and lines (95% confidence interval). The lines reflect 95% confidence intervals. GLMM = general linear mixed model.

| Variable | Effect Size | Lower CI | Upper CI | I^2^ | Prediction  interval | Total number of tests / individuals |
| --- | --- | --- | --- | --- | --- | --- |
| Case detection | 3.9 | 2.0 | 7.6 | 100 | (0.0, 77.8) | 21,056,338 |
| Test positivity | 0.9 | 0.5 | 1.9 | 100 | (0.0, 39.6) | 54,150,480 |
| Missed cases proportion | 27.4 | 9.6 | 57.3 | 96.5 | (2.1, 87.1) | 458 |
| False positive proportion | 0.5 | 0.2 | 1.2 | 97.5 | (0.0, 6.7) | 126,456 |
| Invalid test proportion | 0.3 | 0.1 | 0.7 | 98.9 | (0.0, 19.4) | 2,191,650 |
| Test uptake | 56.7 | 42.3 | 70.0 | 99.8 | (7.2, 95.7) | 29,162 |
| Test schedule adherence | 76.0 | 65.7 | 83.9 | 100 | (15.3, 98.2) | 4,492,534 |
| Result reporting | 85.8 | 76.5 | 91.8 | 99.9 | (24.4, 99.1) | 78,482 |

**Table.** Effect sizes, confidence intervals, and measure of heterogeneity when employing an inverse-variance weighted meta-analysis. CI = Confidence Interval.

# Text S6 – Methods: details of subgroup analysis and cost adjustments

The subgroups listed below were defined in the study protocol and relevant data was extracted. However, data was too heterogenous to perform a meta-analysis:

- SARS-CoV-2 exposure (no known exposure, known exposure – single or multiple, unknown / not defined)
- Test distributor (workplace, school/university, publicly available)
- Cost per person to be tested (tests free of charge vs. payment required)
- Setting (rural or urban, gross domestic product (GDP) per capita [categorized by World Trade Organization], literacy of the target population)
- Assistance in sample collection or performing the test (no assistance vs. assisted in person by trained operator vs. assisted virtually by trained operator vs. assisted in any other way)
- Risk of COVID-19 related morbidity and mortality (as defined by study)
- Vaccination status (vaccinated vs. unvaccinated)
- Prior infection (convalescent vs. no known infection)
- Age (persons ≤ 18 years of age, persons 18 – 65 years of age, persons > 65 years of age)
- Misuse, adverse events, social harms (specific to self-testing vs. issues that occur in general for SARS-CoV-2 testing)
- Additional interventions (any other interventions that were implemented to stop transmission besides C19ST for SARS-CoV-2, such as mask wearing or travel restrictions)

For any costs presented in currencies other than United States Dollars (USD), values were converted to USD using the exchange rate of the date the respective manuscript was published [21]. In addition, where applicable, costs were inflation-adjusted to 2024 USD, using the World Bank GDP deflator [22].

# Text S7 – Results: assessment of publication bias

This section presents funnel plots for the meta-analyses of each of the eight outcomes. These plots display the log odds of the study outcomes against the square root of the study sizes. This approach has been recommended for binary outcomes instead of plotting against standard errors because the standard error is a function of the outcome proportion itself, which can distort funnel-plot shape when event rates approach 0 or 1 [20]. Larger studies (toward the top of the plot) are expected to cluster more tightly around the pooled log-odds estimate, while smaller studies (toward the bottom) will show wider scatter due to greater sampling variability. A symmetric shape around the pooled estimate suggests an absence of small-study effects or selective reporting. Asymmetry, such as smaller studies showing systematically higher or lower log-odds than larger studies, may indicate publication bias or sources of heterogeneity.

For several of the outcomes (missed cases proportion, false positives, invalid test proportion, test uptake, and test schedule adherence), we found an apparent relationship between the study size and the outcome estimate. However, such relationship does not necessarily stem from differences in publishing positive compared to negative study results. Instead, heterogeneity in funnel plots may also result from inter-study heterogeneity, e.g., through larger studies addressing different study populations than smaller studies [85]. For the outcomes assessed in our analysis, as described in the main manuscript, we found studies to assess very different populations, leading to large heterogeneity in our study results. In addition, we found data both supporting and opposing the implementation of COVID-19 self-testing (C19ST) to be published widely. Therefore, we believe the relationship between study size and the outcome estimate found in the funnel plots below to result from inter-study heterogeneity rather than being a consequence of publication bias.

| Funnel plot for the outcome  **case detection**  **** | Funnel plot for the outcome  **missed cases proportion**  **** |
| --- | --- |

| Funnel plot for the outcome  **test positivity**  **** | Funnel plot for the outcome  **false positives**  **** |
| --- | --- |
| Funnel plot for the outcome  **invalid test proportion**  **** | Funnel plot for the outcome  **test uptake**  **** |
| Funnel plot for the outcome  **result reporting**  **** | Funnel plot for the outcome  **test schedule adherence**  **** |

# Text S8 – Results: subgroup analysis of the outcome case detection

We assessed the number of SARS-CoV-2 cases detected by COVID-19 self-testing per 1,000 individuals tested (outcome “case detection”). The overall results for this outcome are provided in the main manuscript (see Figure 2 and Table 3). The forest plot below presents the results of the subgroup analysis for this outcome.


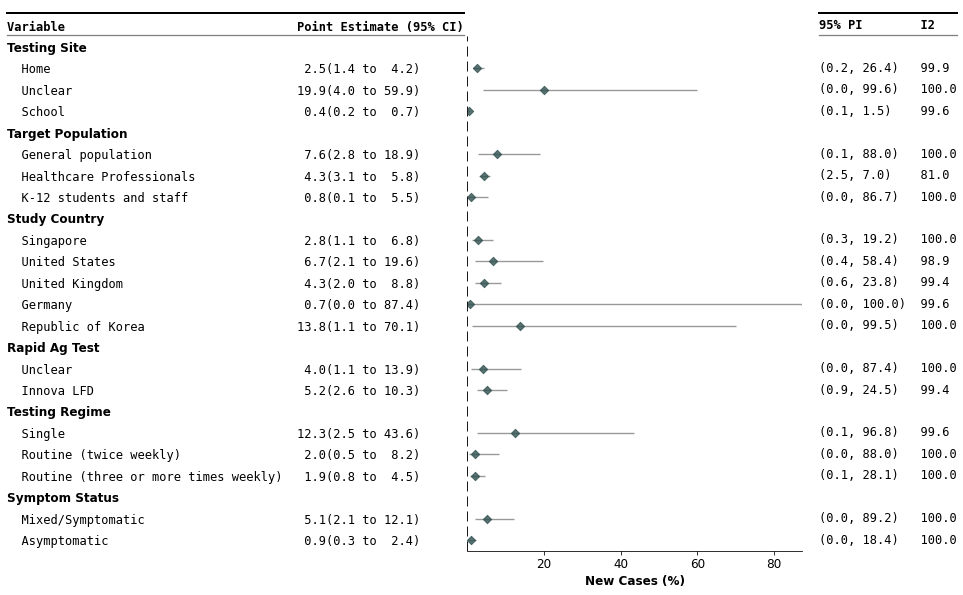


***Caption:*** CI = Confidence interval; I^2^ = Inconsistency index; PI = Prediction interval.

The studies included in each of these subgroups are shown in the table below.

| **Variable** | | **Studies included** |
| --- | --- | --- |
| Testing Site | Home | Agusti, C., et al. [24], Chan, H.K., et al. [26], Chong, S.J., et al. [28], Downs, L.O., et al. [30], Engels, G., et al. [32], Harmon, A., et al. [34], Herbert, C., et al. [35], Hu, Y., et al. [40], Koirala, A., et al. [45], Kwan, T.H., et al. [46], Lamb, G., et al. [47], Lau, C.S., et al. [48], Love, N.K., et al. [49], Love, N.K., et al. [50], Stemler, J., et al. [63], Stohr, J.J.J.M., et al. [64], Tinker, S.C., et al. [65], UK Health Security Agency [69] |
|  | Unclear | Pudasaini, S., et al. [58], Tsang, N.N.Y., et al. [66], Tsao, J., et al. [67], Yun, G., et al. [75], Nacov, J. A. et al. [79], Qasmieh, S.A. et al. [80] |
|  | School | Nakgul, L., et al. [55], Willeit, P., et al. [72] |
| Target Population | General population | Bresser, M., et al. [25], Chong, S.J., et al. [28], Harmon, A., et al. [34], Herbert, C., et al. [35], Kwan, T.H., et al. [46], Lau, C.S., et al. [48], Love, N.K., et al. [49], Love, N.K., et al. [50], Stemler, J., et al. [63], Stohr, J.J.J.M., et al. [64], Tsang, N.N.Y., et al. [66], Nacov, J. A. et al. [79], Qasmieh, S.A. et al. [80] |
|  | Healthcare Professionals | Agusti, C., et al. [24], Downs, L.O., et al. [30], Lamb, G., et al. [47], UK Health Security Agency [69] |
|  | K-12 students and staff | Agusti, C., et al. [24], Engels, G., et al. [32], Koirala, A., et al. [45], Nakgul, L., et al. [55], Pudasaini, S., et al. [58], Willeit, P., et al. [72], Yun, G., et al. [75] |
| Study Country | Singapore | Chong, S.J., et al. [28], Lau, C.S., et al. [48] |
|  | United States | Harmon, A., et al. [34], Herbert, C., et al. [35], Tinker, S.C., et al. [65], Tsao, J., et al. [67], Qasmieh, S.A. et al. [80] |
|  | United Kingdom | Downs, L.O., et al. [30], Hirst, J.A., et al. [37], Lamb, G., et al. [47], Love, N.K., et al. [49], Love, N.K., et al. [50], UK Health Security Agency [69] |
|  | Germany | Engels, G., et al. [32], Pudasaini, S., et a. [58], Stemler, J., et al. [63], Nacov, J. A. et al. [79] |
|  | Republic of Korea | Yun, G., et al. [75] |
| Rapid Ag Test | Unclear | Chong, S.J., et al. [28], Koirala, A., et al. [45], Kwan, T.H., et al. [46], Pudasaini, S., et al. [58], UK Health Security Agency [69], Yun, G., et al. [75], Qasmieh, S.A. et al. [80] |
|  | Innova LFD | Downs, L.O., et al. [30], Hirst, J.A., et al. [37], Lamb, G., et al. [47], Love, N.K., et al. [49], Love, N.K., et al. [50] |
| Testing Regime | Single | Agusti, C., et al. [24], Bresser, M., et al. [25], Stemler, J., et al. [63], Stohr, J.J.J.M., et al. [64], Tsao, J., et al. [67], Nacov, J. A. et al. [79] |
|  | Routine (twice weekly) | Downs, L.O., et al. [30], Engels, G., et al. [32], Harmon, A., et al. [34], Herbert, C., et al. [35], Lamb, G., et al. [47], Nakgul, L., et al. [55], Tinker, S.C., et al. [65], Willeit, P., et al. [72], Yun, G., et al. [75] |
|  | Routine (three or more times weekly) | Chong, S.J., et al. [28], Hu, Y., et al. [40], Koirala, A., et al. [45], Kwan, T.H., et al. [46], Love, N.K., et al. [49], Love, N.K., et al. [50], Pudasaini, S., et al. [58], UK Health Security Agency [69] |
| Symptom Status | Mixed / Symptomatic | Agusti, C., et al. [24], Bresser, M., et al. [25], Chan, H.K., et al. [26], Chong, S.J., et al. [28], Harmon, A., et al. [34], Herbert, C., et al. [35], Hirst, J.A., et al. [37], Hu, Y., et al. [40], Koirala, A., et al. [45], Kwan, T.H., et al. [46], Lamb, G., et al. [47], Lau, C.S., et al. [48], Nakgul, L., et al. [55], Pudasaini, S., et al. [58], Stemler, J., et al. [63], Stohr, J.J.J.M., et al. [64], Tsang, N.N.Y., et al. [66], Tsao, J., et al. [67], UK Health Security Agency [69], Yun, G., et al. [75], Nacov, J. A. et al. [79], Qasmieh, S.A. et al. [80] |
|  | Asymptomatic | Downs, L.O., et al. [30], Engels, G., et al. [32], Love, N.K., et al. [49], Love, N.K., et al. [50], Tinker, S.C., et al. [65], Willeit, P., et al. [72], Yun, G., et al. [75] |

# Text S9 – Results: subgroup analysis of the outcome missed cases proportion

We assessed the proportion of individuals who were SARS-CoV-2 positive by RT-PCR but received negative results only when conducting COVID-19 self-testing (outcome “missed cases proportion”). The overall results for this outcome are provided in the main manuscript (see Figure 2 and Table 3). The forest plot below presents the results of the subgroup analysis for this outcome.


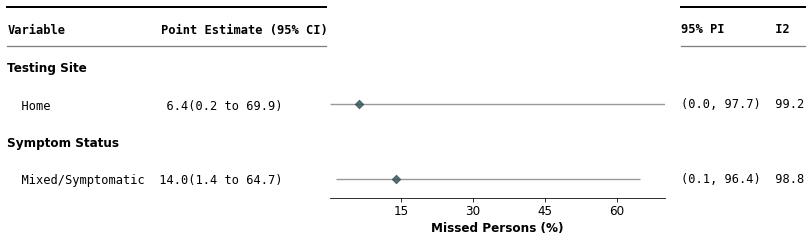


***Caption:*** CI = Confidence interval; I^2^ = Inconsistency index; PI = Prediction interval.

The studies included in each of these subgroups are shown in the table below.

| **Variable** | | **Studies included** |
| --- | --- | --- |
| Testing | Home | Harmon, A., et al. [34], Hu, Y., et al. [40], Stohr, J.J.J.M., et al. [64] |
| Symptom Status | Mixed / Symptomatic | Harmon, A., et al. [34], Hu, Y., et al. [40], Stohr, J.J.J.M., et al. [64], Tsao, J., et al. [67] |

The C19ST test results in studies also reporting confirmatory testing of negative C19ST results are presented below. Of note, to reduce heterogeneity, only data sets where all individuals reporting a positive or negative COVID-19 self-testing (C19ST) result also received confirmatory testing by RT-PCR were included in the meta-analysis (respective data sets are marked with * and colored in blue).

| **Dataset**  **author**  **[reference to study]** | Individuals  reporting a C19ST  result | **Results of COVID-19 self-testing (C19ST; number of individuals)** | | | |
| --- | --- | --- | --- | --- | --- |
|  |  | Individuals with a positive C19ST result | Individuals with a negative C19ST result | Individuals with an invalid C19ST result | Individuals  having falsely interpreted the C19ST result |
| Bresser, M.,  et al. [25] | 1123 | 9 | 1107 | 5 | # |
| Chen, S.H.,  et al. [27] | # | # | # | # | # |
| Chen, S.H.,  et al. [27] | # | # | # | # | # |
| Engels, G.,  et al. [32] | 280 | 0 | 280 | # | # |
| * Harmon, A., et al. [34] | 257 | 15 | 242 | # | # |
| Hirst, J. A.,  et al. [37] | 551 | 8 | # | # | # |
| Hoehl, S.,  et al. [38] | 635 | # | # | # | # |
| * Hu, Y.,  et al. [40] | 17655 | 26 | 17629 | # | # |
| Kheiroddin, P., et al. [42] | # | 2 | 9 | # | # |
| Koirala, A.,  et al. [45] | 9887 | 70 | # | # | # |
| Lamb, G.,  et al. [47] | 5076 | 284 | 4730 | 35 | 27 |
| * Lau, C.S.,  et al. [48] | 160 | 0 | 160 | # | # |
| Love, N.K.,  et al. (b) [50] | 570 | 102 | 464 | 4 | 8 |
| Nakgul, L.,  et al. [55] | 133 | 0 | 133 | # | # |
| * Stohr, J. J. J. M., et al. [64] | 1595 | 88 | 1476 | 31 | # |
| * Stohr, J. J. J. M., et al. [64] | 1606 | 122 | 1467 | 17 | # |
| Tinker, S.C., et al. [65] | 1347 | 11 | # | 0 | # |
| * Tsao, J.,  et al. [67] | 723 | 47 | 676 | # | # |
| Wachinger, J., et al. [71] | # | 0 | # | # | # |

RT-PCR = reverse transcriptase polymerase chain reaction; Ag-RDT = antigen detecting rapid diagnostic test

The results of the confirmatory testing for each of the C19ST results are shown below (as for the table above, data sets included in the meta-analysis are marked with * and colored in blue).

| **Dataset**  **author**  **[reference to study]** | **Confirmatory testing (number of individuals)** | | | | | |
| --- | --- | --- | --- | --- | --- | --- |
|  | Method | Sample type | Individuals true positive on C19ST | Individuals false negative on C19ST (“**missed**  **individual**”) | Individuals true negative on C19ST | Individuals false positive on C19ST |
| Bresser, M.,  et al. [25] | Professional Ag-RDT | # | 9 | 1 | 1107 | # |
| Chen, S.H.,  et al. [27] | RT-PCR | naso-  pharyngeal | 186 | 38 | 24 | 1 |
| Chen, S.H.,  et al. [27] | RT-PCR | naso-  pharyngeal | 78 | 29 | 36 | 5 |
| Engels, G.,  et al. [32] | Antibody | # | # | 0 | # | # |
| * Harmon, A. et al. [34] | RT-PCR | anterior  nasal / mid-turbinate | 15 | 0 | 242 | 0 |
| Hirst, J. A.,  et al. [37] | # | # | 3 | 5 | # | 2 |
| Hoehl, S.,  et al. [38] | RT-PCR | # | # | 4 | # | # |
| * Hu, Y.  et al. [40] | RT-PCR | # | 26 | 0 | 17629 | 0 |
| Kheiroddin, P., et al. [42] | RT-PCR | gargle  lavage | 2 | 9 | # | 0 |
| Koirala, A.  et al. [45] | RT-PCR | # | 2 | 4 | 844 | 6 |
| Lamb, G.,  et al. [47] | RT-PCR | naso-  pharyngeal | 244 | 36 | # | 15 |
| * Lau, C.S.,  et al. [48] | RT-PCR | # | 0 | 0^+^ | 160 | 0 |
| Love, N.K.,  et al. (2) [50] | RT-PCR | anterior  nasal / mid-turbinate | 53 | 11 | 281 | 1 |
| Nakgul, L.  et al. [55] | Ag-RDT self-test | nasal | # | 2 | # | # |
| * Stohr, J. J. J. M., et al. [64] | RT-PCR | naso- / oropharyngeal | 86 | 89 | 1379 | 2 |
| * Stohr, J. J. J. M., et al. [64] | RT-PCR | naso- / oropharyngeal | 118 | 74 | 1387 | 4 |
| Tinker, S.C., et al. [65] | RT-PCR and  antibody | # | 3 | 4 | 317 | 8 |
| * Tsao, J.  et al. [67] | RT-PCR | # | 23 | 27 | 649 | 1 |
| Wachinger, J., et al. [71] | RT-PCR | # | 0 | 1 | # | 0 |

RT-PCR = reverse transcriptase polymerase chain reaction; Ag-RDT = antigen detecting rapid diagnostic test

^+^  One additional individual participated in the Lau, C. S., et al. [48] study and was missed by C19ST. However, this occurred before every individual received confirmatory testing by RT-PCR, wherefore this individual was not considered for the meta-analysis. When including this individual in the meta-analysis, the overall proportion of missed cases would have been estimated at 20% (95% CI: 3-70%; compared to 14% [95% CI: 1-65%] estimated in the main analysis).

# Text S10 – Results: subgroup analysis of the outcome test positivity

We assessed the proportion of positive COVID-19 self-testing (C19ST) results out of all individual C19ST results (outcome “test positivity”). The overall results for this outcome are provided in the main manuscript (see Figure 3 and Table 3). The forest plot below presents the results of the subgroup analysis for this outcome.


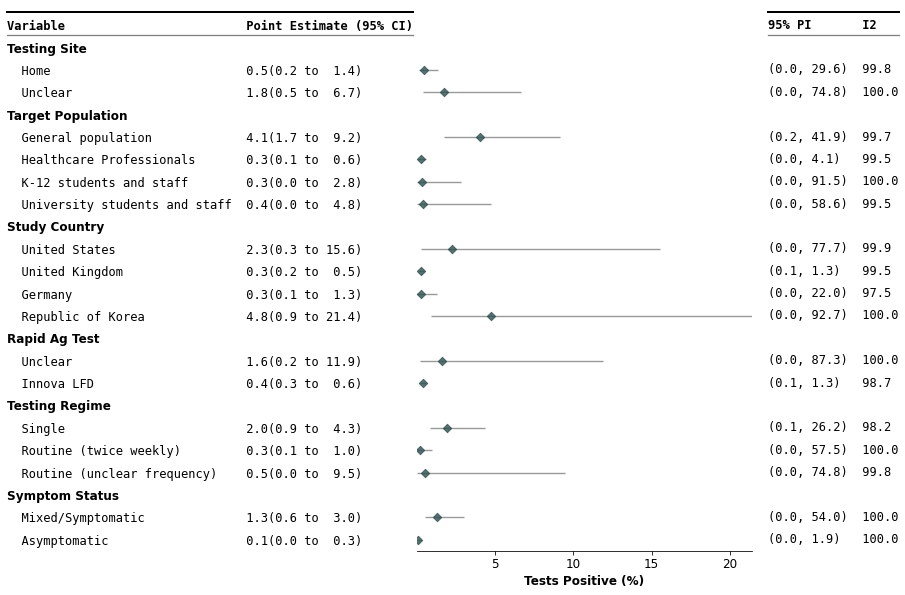


***Caption:*** CI = Confidence interval; I^2^ = Inconsistency index; PI = Prediction interval.

The studies included in each of these subgroups are shown below.

| **Variable** | | **Studies included** |
| --- | --- | --- |
| Testing Site | Home | Agusti, C., et al. [24], D'Agostino, E.M., et al. [29], Downs, L.O., et al. [30], Engels, G., et al. [32], Harmon, A., et al. [34], Hoehl, S., et al. [38], Iftner, T., et al. [41], Lamb, G., et al. [47], Stemler, J., et al. [63], Stohr, J.J.J.M., et al. [64], Tinker, S.C., et al. [65], UK Health Security Agency [69], U. o. Liverpool [70], Wu, S., et al. [74] |
|  | Unlcear | Hogg, C., et al. [39], Kim, A.E., et al. [44], Ryan, F., et al. [59], Tsao, J., et al. [67], Yun, G., et al. [75], Davies, M. et al. [76], Nacov, J. A. et al. [79] |
| Target Population | General population | Bresser, M., et al. [25], D'Agostino, E.M., et al. [29], Harmon, A., et al. [34], Marbán‐Castro, E., et al. [51], Stemler, J., et al. [63], Stohr, J.J.J.M., et al. [64], U. o. Liverpool [70], Nacov, J. A. et al. [79] |
|  | Healthcare Professionals | Agusti, C., et al. [24], Downs, L.O., et al. [30], Hogg, C., et al. [39], Iftner, T., et al. [41], Lamb, G., et al. [47], Ryan, F., et al. [59], Tulloch, J.S.P., et al. [68], UK Health Security Agency [69], Wu, S., et al. [74] |
|  | K-12 students and staff | Agusti, C., et al. [24], Engels, G., et al. [32], Hoehl, S., et al. [38], Nakgul, L., et al. [55], Yun, G., et al. [75] |
|  | University students and staff | Hirst, J.A., et al. [37], Kim, A.E., et al. [44], Tinker, S.C., et al. [65], Tsao, J., et al. [67] |
| Study Country | United States | D'Agostino, E.M., et al. [29], Harmon, A., et al. [34], Kim, A.E., et al. [44], Tinker, S.C., et al. [65], Tsao, J., et al. [67] |
|  | United Kingdom | Downs, L.O., et al. [30], Hirst, J.A., et al. [37], Hogg, C., et al. [39], Lamb, G., et al. [47], Ryan, F., et al. [59], Tulloch, J.S.P., et al. [68], UK Health Security Agency [69], U. o. Liverpool [70], Davies, M. et al. [76] |
|  | Germany | Engels, G., et al. [32], Hoehl, S., et al. [38], Iftner, T., et al. [41], Stemler, J., et al. [63], Nacov, J. A. et al. [79] |
|  | Republic of Korea | Yun, G., et al. [75] |
| Rapid Antigen Test | Unclear | Kim, A.E., et al. [44], UK Health Security Agency [69], Yun, G., et al. [75], Davies, M. et al. [76] |
|  | Innova LFD | Downs, L.O., et al. [30], Hirst, J.A., et al. [37], Hogg, C., et al. [39], Lamb, G., et al. [47], Tulloch, J.S.P., et al. [68], U. o. Liverpool [70] |
| Testing Regime | Single | Agusti, C., et al. [24], Bresser, M., et al. [25], Iftner, T., et al. [41], Stemler, J., et al. [63], Stohr, J.J.J.M., et al. [64], Tsao, J., et al. [67], Nacov, J. A. et al. [79] |
|  | Routine (twice weekly) | Downs, L.O., et al. [30], Engels, G., et al. [32], Harmon, A., et al. [34], Lamb, G., et al. [47], Nakgul, L., et al. [55], Ryan, F., et al. [59], Tinker, S.C., et al. [65], Tulloch, J.S.P., et al. [68], Wu, S., et al. [74], Yun, G., et al. [75] |
|  | Routine (unclear frequency) | Hirst, J.A., et al. [37], Hogg, C., et al. [39], Marbán‐Castro, E., et al. [51] |
| Symptom Status | Mixed / Symptomatic | Agusti, C., et al. [24], Bresser, M., et al. [25], D'Agostino, E.M., et al. [29], Harmon, A., et al. [34], Hirst, J.A., et al. [37], Hoehl, S., et al. [38], Hogg, C., et al. [39], Iftner, T., et al. [41], Kim, A.E., et al. [44], Lamb, G., et al. [47], Marbán‐Castro, E., et al. [51], Nakgul, L., et al. [55], Stemler, J., et al. [63], Stohr, J.J.J.M., et al. [64], Tsao, J., et al. [67], Tulloch, J.S.P., et al. [68], UK Health Security Agency [69], U. o. Liverpool [70], Wu, S., et al. [74], Yun, G., et al. [75], Nacov, J. A. et al. [79] |
|  | Asymptomatic | Downs, L.O., et al. [30], Engels, G., et al. [32], Iftner, T., et al. [41], Ryan, F., et al. [59], Tinker, S.C., et al. [65], Yun, G., et al. [75], Davies, M. et al. [76] |

# Text S11 – Results: subgroup analysis of the outcome false positives

We assessed the proportion of negative reverse-transcriptase polymerase chain reaction test results that falsely were positive using COVID-19 self-testing (C19ST) (outcome “false positives”). The overall results for this outcome are provided in the main manuscript (see Figure 3 and Table 3). The forest plot below presents the results of the subgroup analysis for this outcome.


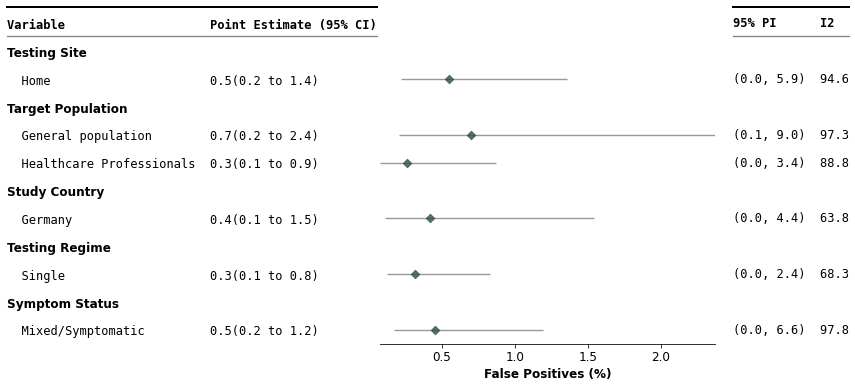


***Caption:*** CI = Confidence interval; I^2^ = Inconsistency index; PI = Prediction interval.

The studies included in each of these subgroups are shown in the table below.

| **Variable** | | **Study included** |
| --- | --- | --- |
| Testing Site | Home | Harmon, A., et al. [34], Iftner, T., et al. [41], Smit, T., et al. [60], Stohr, J.J.J.M., et al. [64] |
| Target Population | General population | Harmon, A., et al. [34], Smit, T., et al. [60], Stohr, J.J.J.M., et al. [64] |
|  | Healthcare Professionals | Hogg, C., et al. [39], Iftner, T., et al. [41] |
| Study Country | Germany | Iftner, T., et al. [41] |
| Testing Regime | Single | Iftner, T., et al. [41], Stohr, J.J.J.M., et al. [64] |
| Symptom Status | Mixed / Symptomatic | Harmon, A., et al. [34], Hogg, C., et al. [39], Iftner, T., et al. [41], Smit, T., et al. [60], Stohr, J.J.J.M., et al. [64] |

The C19ST test results in studies also reporting confirmatory testing of positive C19ST results are presented in the table below. To reduce heterogeneity, only data sets where all individuals reporting a positive or negative C19ST result also received confirmatory testing by RT-PCR were included the meta-analysis (respective data sets are marked with * and colored in blue).

| **Dataset author**  **[reference to study]** | **Number of C19ST reported** | **Results of COVID-19 self-testing**  **(C19ST; number of tests)** | | | | |
| --- | --- | --- | --- | --- | --- | --- |
|  |  | Positive | Negative | Invalid | False  interpretation |  |
| Bresser et al. [25] | 1121 | 9 | 1107 | 5 | # |  |
| Chen, S.H., et al. [27] | # | # | # | # | # |  |
| Chen, S.H., et al. [27] | # | # | # | # | # |  |
| Downs, L. O., et al. [30] | 46503 | 328 | 45710 | 465 | # |  |
| * Harmon, A. et al. [34] | 2951 | 144 | 2807 | # | # |  |
| Hoehl, S., et al. [38] | 11385 | 21 | 11315 | 49 | # |  |
| * Hogg, C. et al. [39] | 115593 | 229 | 115364 | 0 | 0 |  |
| * Iftner, T., et al. [41] | 468 | 1 | 462 | 5 | # |  |
| * Iftner, T., et al. [41] | 479 | 10 | 466 | 3 | # |  |
| * Iftner, T., et al. [41] | 464 | 1 | 453 | 10 | # |  |
| * Iftner, T., et al. [41] | 481 | 1 | 462 | 18 | # |  |
| Kheiroddin, P., et al. [42] | # | 2 | 9 | 0 | 0 |  |
| Kim, A. E. et al. [44] | 17572 | 1350 | # | # | # |  |
| Lamb, G., et al. [47] | 45022 | 284 | 44676 | 35 | 27 |  |
| Ryan, F. et al. [59] | 1094413 | 1142 | 1093271 | # | # |  |
| * Smit, T. et al. [60] | # | 1475 | 4096 | # | # |  |
| * Stohr, J. J. J. M., et al. [64] | 1595 | 88 | 1476 | 31 | # |  |
| * Stohr, J. J. J. M., et al. [64] | 1606 | 122 | 1467 | 17 | # |  |
| Tinker, S.C., et al. [65] | 9971 | 2 | # | # | # |  |
| Tsao, J. et al. [67] | 723 | 47 | 676 | # | # |  |
| Tulloch, J. S. P., et al. [68] | 1638 | 5 | 1633 | 0 | 0 |  |
| UK Health Security Agency [69] | 719 | 1 | # | # | # |  |
| U.o.Liverpool [70] | 1900302 | 13942 | # | 0 | # |  |
| Wu, S., et al. [74] | 156000 | 31 | # | # | # |  |

RT-PCR = reverse transcriptase polymerase chain reaction; Ag-RDT = antigen detecting rapid diagnostic test.

The results of the confirmatory testing for each of the C19ST results are shown in the table below (as for the table above, data sets included in the meta-analysis are marked with * and colored in blue).

| **Dataset author**  **[reference to study]** | **Confirmatory testing (number of tests)** | | | | | |
| --- | --- | --- | --- | --- | --- | --- |
|  | Method | Sample type | True positive | False negative | True negative | False positive |
| Bresser et al. [25] | Professional Ag-RDT testing | # | 9 | 0 | 1107 | 0 |
| Chen, S.H., et al. [27] | RT-PCR | naso-  pharyngeal | 186 | 38 | 24 | 1 |
| Chen, S.H., et al. [27] | RT-PCR | naso-  pharyngeal | 78 | 29 | 36 | 5 |
| Downs, L. O., et al. [30] | RT-PCR | naso- / oro-  pharyngeal | 161 | # | # | 8 |
| * Harmon, A. et al. [34] | RT-PCR | anterior nasal / mid turbinate | 60 | 16 | 2791 | 84 |
| Hoehl, S., et al. [38] | RT-PCR | # | 5 | # | # | 16 |
| * Hogg, C. et al. [39] | RT-PCR | combined nose and throat / dry throat swabs | 128 | 371 | 114993 | 101 |
| * Iftner, T., et al. [41] | RT-PCR | oro-  pharyngeal | 0 | 0 | 462 | 1 |
| * Iftner, T., et al. [41] | RT-PCR | oro-  pharyngeal | 0 | 0 | 466 | 10 |
| * Iftner, T., et al. [41] | RT-PCR | oro-  pharyngeal | 0 | 0 | 453 | 1 |
| * Iftner, T., et al. [41] | RT-PCR | oro-  pharyngeal | 0 | 0 | 462 | 1 |
| Kheiroddin, P., et al. [42] | RT-PCR | gargle  lavage | 2 | 9 | 0 | 0 |
| Kim, A. E. et al. [44] | RT-PCR | anterior  nasal | 456 | # | 6762 | 82 |
| Lamb, G., et al. [47] | RT-PCR | naso-  pharyngeal | 244 | # | # | 15 |
| Ryan, F. et al. [59] | RT-PCR | combined nose and throat | 440 | 1053 | 642672 | 315 |
| * Smit, T. et al. [60] | RT-PCR | nose and throat | 1417 | 295 | 3801 | 58 |
| * Stohr, J. J. J. M., et al. [64] | RT-PCR | naso- / oro-pharyngeal | 86 | 89 | 1379 | 2 |
| * Stohr, J. J. J. M., et al. [64] | RT-PCR | naso- / oro-pharyngeal | 118 | 74 | 1387 | 4 |
| Tinker, S.C., et al. [65] | RT-PCR | # | 2 | # | 661 | 0 |
| Tsao, J. et al. [67] | RT-PCR | # | 23 | 27 | 649 | 1 |
| Tulloch, J. S. P., et al. [68] | RT-PCR | # | 5 | # | 823 | 0 |
| UK Health Security Agency [69] | RT-PCR | # | 1 | # | 55 | 0 |
| U.o.Liverpool [70] | RT-PCR | # | 8495 | # | # | 1314 |
| Wu, S., et al. [74] | RT-PCR | # | 20 | # | # | 11 |

RT-PCR = reverse transcriptase polymerase chain reaction; Ag-RDT = antigen detecting rapid diagnostic test.

# Text S12 – Results: analysis of the outcome invalid test proportion

We assessed the proportion of the proportion of COVID-19 self-testing (C19ST) results with missing control line or uninterpretable (outcome “invalid test proportion”). The upper forest plot presents the results of the overall analysis, while the results of the subgroup analysis are presented in the lower forest plot.


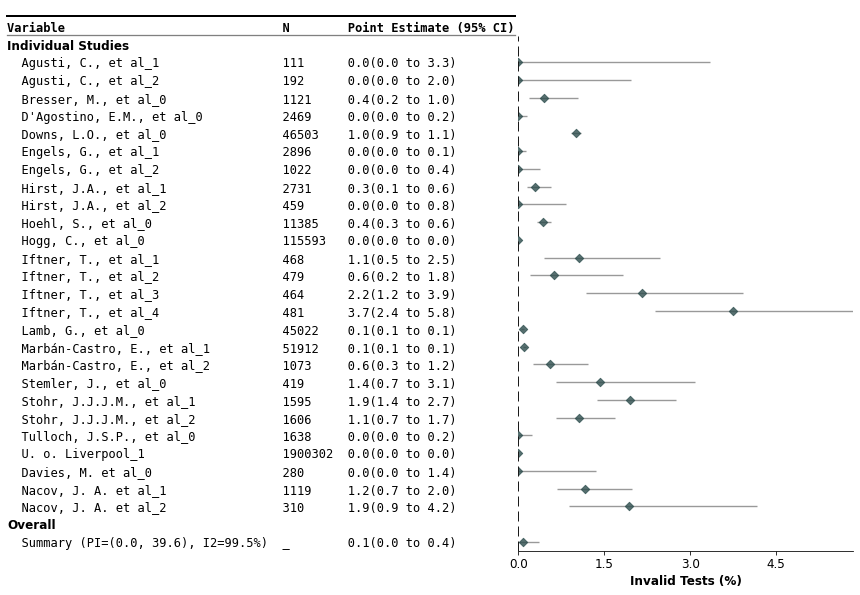


***Caption:*** CI = Confidence interval; I^2^ = Inconsistency index; PI = Prediction interval; n = number of tests.


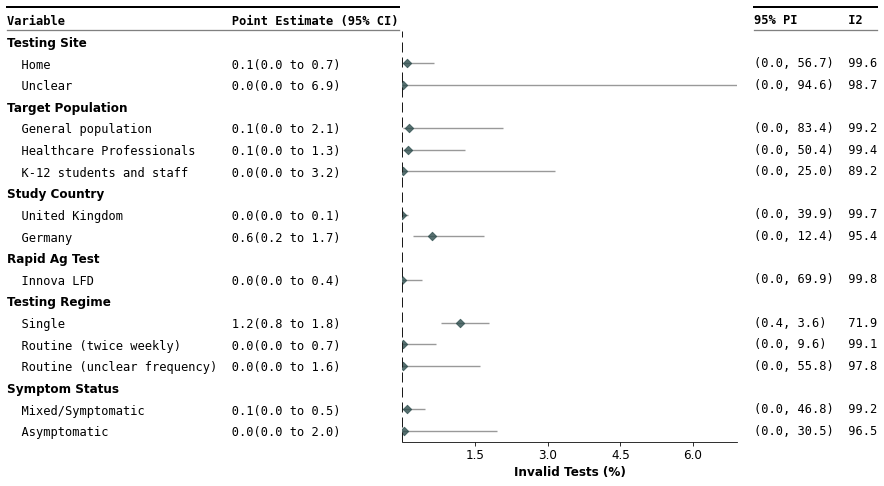


***Caption:*** CI = Confidence interval; I^2^ = Inconsistency index; PI = Prediction interval; n = number of tests.

The studies included in each of the subgroups are shown in the table below.

| **Variable** | | **Studies included** |
| --- | --- | --- |
| Testing site | Home | Agusti, C., et al. [24], D'Agostino, E.M., et al. [29], Downs, L.O., et al. [30], Engels, G., et al. [32], Hoehl, S., et al. [38], Iftner, T., et al. [41], Lamb, G., et al. [47], Stemler, J., et al. [63], Stohr, J.J.J.M., et al. [64], U. o. Liverpool [70] |
|  | Unclear | Hogg, C., et al. [39], Davies, M. et al. [76], Nacov, J. A. et al. [79] |
| Target Population | General population | Bresser, M., et al. [25], D'Agostino, E.M., et al. [29], Marbán‐Castro, E., et al. [51], Stemler, J., et al. [63], Stohr, J.J.J.M., et al. [64], U. o. Liverpool [70], Nacov, J. A. et al. [79] |
|  | Healthcare professionals | Agusti, C., et al. [24], Downs, L.O., et al. [30], Hogg, C., et al. [39], Iftner, T., et al. [41], Lamb, G., et al. [47], Tulloch, J.S.P., et al. [68] |
|  | K-12 students and staff | Agusti, C., et al. [24], Engels, G., et al. [32], Hoehl, S., et al. [38] |
| Study country | United Kingdom | Downs, L.O., et al. [30], Hirst, J.A., et al. [37], Hogg, C., et al. [39], Lamb, G., et al. [47], Tulloch, J.S.P., et al. [68], U. o. Liverpool [70], Davies, M. et al. [76] |
|  | Germany | Engels, G., et al. [32], Hoehl, S., et al. [38], Iftner, T., et al. [41], Stemler, J., et al. [63], Nacov, J. A. et al. [79] |
| Rapid Ag Test | Innova LFD | Downs, L.O., et al. [30], Hirst, J.A., et al. [37], Hogg, C., et al. [39], Lamb, G., et al. [47], Tulloch, J.S.P., et al. [68], U. o. Liverpool [70] |
| Testing Regime | Single | Agusti, C., et al. [24], Bresser, M., et al. [25], Iftner, T., et al. [41], Stemler, J., et al. [63], Stohr, J.J.J.M., et al. [64], Nacov, J. A. et al. [79] |
|  | Routine (twice weekly) | Downs, L.O., et al. [30], Engels, G., et al. [32], Lamb, G., et al. [47], Tulloch, J.S.P., et al. [68] |
|  | Routine (unclear frequency) | Hirst, J.A., et al. [37], Hogg, C., et al. [39], Marbán‐Castro, E., et al. [51] |
| Symptom status | Mixed / Symptomatic | Agusti, C., et al. [24], Bresser, M., et al. [25], D'Agostino, E.M., et al. [29], Hirst, J.A., et al. [37], Hoehl, S., et al. [38], Hogg, C., et al. [39], Iftner, T., et al. [41], Lamb, G., et al. [47], Marbán‐Castro, E., et al. [51], Stemler, J., et al. [63], Stohr, J.J.J.M., et al. [64], Tulloch, J.S.P., et al. [68], U. o. Liverpool. [70], Nacov, J. A. et al. [79] |
|  | Asymptomatic | Downs, L.O., et al. [30], Engels, G., et al. [32], Iftner, T., et al. [41], Davies, M. et al. [76] |

# Text S13 – Results: subgroup analysis of the outcome test uptake

We assessed the proportion of study participants that voluntarily participated in a COVID-19 self-testing (C19ST) study (outcome “uptake”). The overall results for this outcome are provided in the main manuscript (see Figure 4 and Table 3). The forest plot below presents the results of the subgroup analysis for this outcome.


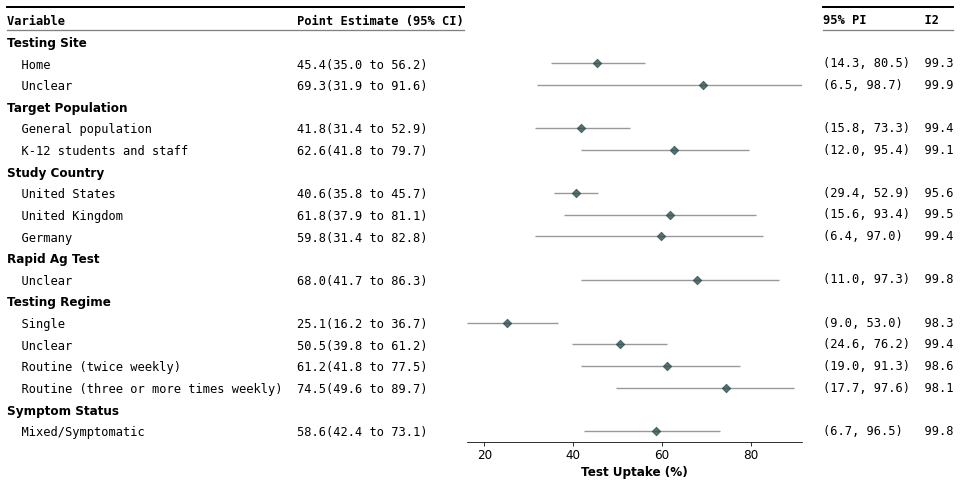


***Caption:*** CI = Confidence interval; I^2^ = Inconsistency index; PI = Prediction interval; n = number of tests.

The studies included in each of the subgroups are shown in the table below.

| **Variable** | | **Studies included** |
| --- | --- | --- |
| Testing Site | Home | Agusti, C., et al. [24], Chan, H.K., et al. [26], Engels, G., et al. [32], Kiene, S.M., et al. [43], Love, N.K., et al. [50], Nodora, J.N., et al. [56], Stemler, J., et al. [63], UK Health Security Agency [69], Wachinger, J., et al. [71], Qasmieh, S.A. et al. [83] |
|  | Unclear | Pudasaini, S., et al. [58], Del Fiol, G. et al. [77], van Hagen, C.C.E. et al. [82], Qasmieh, S.A. et al. [84] |
| Target Population | General population | Love, N.K., et al. [50], Nodora, J.N., et al. [56], Stemler, J., et al. [63], Del Fiol, G. et al. [77], Mwangoka, G.W. et al. [78], van Hagen, C.C.E. et al. [82], Qasmieh, S.A. et al. [83], Qasmieh, S.A. et al. [84] |
|  | K-12 students and staff | Agusti, C., et al. [24], Engels, G., et al. [32], Kiene, S.M., et al. [43], Nakgul, L., et al. [55], Pudasaini, S., et al. [58], Wachinger, J., et al. [71] |
| Study Country | United States | Kiene, S.M., et al. [43], Nodora, J.N., et al. [56], Del Fiol, G. et al. [77], Qasmieh, S.A. et al. [83], Qasmieh, S.A. et al. [84] |
|  | United Kingdom | Hirst, J.A., et al. [37], Love, N.K., et al. [50], Tulloch, J.S.P., et al. [68], UK Health Security Agency [69] |
|  | Germany | Engels, G., et al. [32], Pudasaini, S., et al. [58], Stemler, J., et al. [63], Wachinger, J., et al. [71] |
| Rapid Ag Test | Unclear | Pudasaini, S., et al. [58], UK Health Security Agency [69], Del Fiol, G. et al. [77], van Hagen, C.C.E. et al. [82], Qasmieh, S.A. et al. [83], Qasmieh, S.A. et al. [84] |
| Testing Regime | Single | Agusti, C., et al. [24], Stemler, J., et al. [63], Mwangoka, G.W. et al. [78] |
|  | Unclear | Chan, H.K., et al. [26], Nodora, J.N., et al. [56], Del Fiol, G. et al. [77], van Hagen, C.C.E. et al. [82], Qasmieh, S.A. et al. [83], Qasmieh, S.A. et al. [84] |
|  | Routine (twice weekly) | Engels, G., et al. [32], Nakgul, L., et al. [55], Papenburg, J., et al. [57], Tulloch, J.S.P., et al. [68] |
|  | Routine (three or more times weekly) | Love, N.K., et al. [50], Pudasaini, S., et al. [58], UK Health Security Agency [69], Wachinger, J., et al. [71] |
| Symptom Status | Mixed / Symptomatic | Agusti, C., et al. [24], Chan, H.K., et al. [26], Hirst, J.A., et al. [37], Kiene, S.M., et al. [43], Marbán‐Castro, E., et al. [51], Nakgul, L., et al. [55], Nodora, J.N., et al. [56], Papenburg, J., et al. [57], Pudasaini, S., et al. [58], Stemler, J., et al. [63], Tulloch, J.S.P., et al. [68], UK Health Security Agency [69], Wachinger, J., et al. [71], Del Fiol, G. et al. [77], Mwangoka, G.W. et al. [78], van Hagen, C.C.E. et al. [82], Qasmieh, S.A. et al. [83], Qasmieh, S.A. et al. [84] |

# Text S14 – Results: subgroup analysis of the outcome test schedule adherence

We assessed the proportion of COVID-19 self-testing (C19ST) test actually performed relative to those requested per study protocol (outcome “test schedule adherence”). The overall results for this outcome are provided in the main manuscript (see Figure 4 and Table 3). The forest plot below presents the results of the subgroup analysis for this outcome.


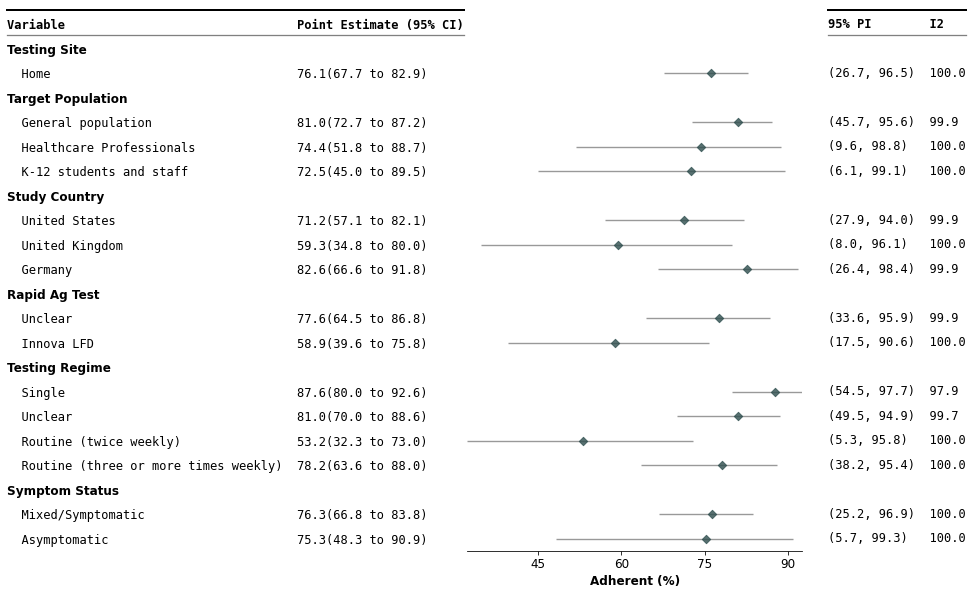


***Caption:*** CI = Confidence interval; I^2^ = Inconsistency index; PI = Prediction interval; n = number of tests.

The studies included in each of the subgroups are shown in the table below.

| **Variable** | | **Studies included** |
| --- | --- | --- |
| Testing Site | Home | Agusti, C., et al. [24], Downs, L.O., et al. [30], Engels, G., et al. [32], Herbert, C., et al. [35], Herbert, C., et al. [36], Hoehl, S., et al. [38], Iftner, T., et al. [41], Koirala, A., et al. [45], Lamb, G., et al. [47], Love, N.K., et al. [49], Love, N.K., et al. [50], McDaniels-Davidson, C., et al. [52], Stemler, J., et al. [63], Stohr, J.J.J.M., et al. [64], Tinker, S.C., et al. [65], UK Health Security Agency [69] |
| Target Population | General population | Herbert, C., et al. [35], Herbert, C., et al. [36], Love, N.K., et al. [49], Love, N.K., et al. [50], Stemler, J., et al. [63], Stohr, J.J.J.M., et al. [64] |
|  | Healthcare Professionals | Agusti, C., et al. [24], Downs, L.O., et al. [30], Iftner, T., et al. [41], Lamb, G., et al. [47], Ryan, F., et al. [59], Tulloch, J.S.P., et al. [68], UK Health Security Agency [69] |
|  | K-12 students and staff | Agusti, C., et al. [24], Engels, G., et al. [32], Hoehl, S., et al. [38], Koirala, A., et al. [45], McDaniels-Davidson, C., et al. [52], Nakgul, L., et al. [55], Willeit, P., et al. [72] |
| Study Country | United States | Herbert, C., et al. [35], Herbert, C., et al. [36], McDaniels-Davidson, C., et al. [52], Tinker, S.C., et al. [65] |
|  | United Kingdom | Downs, L.O., et al. [30], Lamb, G., et al. [47], Love, N.K., et al. [49], Love, N.K., et al. [50], Ryan, F., et al. [59], Tulloch, J.S.P., et al. [68], UK Health Security Agency [69] |
|  | Germany | Engels, G., et al. [32], Hoehl, S., et al. [38], Iftner, T., et al. [41], Stemler, J., et al. [63] |
| Rapid Ag Test | Unclear | Herbert, C., et al. [36], Koirala, A., et al. [45], McDaniels-Davidson, C., et al. [52], UK Health Security Agency [69] |
|  | Innova LFD | Downs, L.O., et al. [30], Lamb, G., et al. [47], Love, N.K., et al. [49], Love, N.K., et al. [50], Tulloch, J.S.P., et al. [68] |
| Testing Regime | Single | Agusti, C., et al. [24], Iftner, T., et al. [41], Stemler, J., et al. [63], Stohr, J.J.J.M., et al. [64] |
|  | Unclear | Herbert, C., et al. [36] |
|  | Routine (twice weekly) | Downs, L.O., et al. [30], Engels, G., et al. [32], Herbert, C., et al. [35], Lamb, G., et al. [47], Nakgul, L., et al. [55], Papenburg, J., et al. [57], Ryan, F., et al. [59], Tinker, S.C., et al. [65], Tulloch, J.S.P., et al. [68], Willeit, P., et al. [72] |
|  | Routine (three or more times weekly) | Hoehl, S., et al. [38], Koirala, A., et al. [45], Love, N.K., et al. [49], Love, N.K., et al. [50], UK Health Security Agency [69] |
| Symptom Status | Mixed / Symptomatic | Agusti, C., et al [24], Herbert, C., et al. [35], Herbert, C., et al. [36], Hoehl, S., et al. [38], Iftner, T., et al. [41], Koirala, A., et al. [45], Lamb, G., et al. [47], McDaniels-Davidson, C., et al. [52], Nakgul, L., et al. [55], Papenburg, J., et al. [57], Stemler, J., et al. [63], Stohr, J.J.J.M., et al. [64], Tulloch, J.S.P., et al. [68], UK Health Security Agency [69] |
|  | Asymptomatic | Downs, L.O., et al. [30], Engels, G., et al. [32], Iftner, T., et al. [41], Love, N.K., et al. [49], Love, N.K., et al. [50], Ryan, F., et al. [59], Tinker, S.C., et al. [65], Willeit, P., et al. [72] |

# Text S15 – Results: subgroup analysis of the outcome result reporting

We assessed the proportion of COVID-19 self-testing (C19ST) results reported to study officials or health authorities (outcome “result reporting”). The overall results for this outcome are provided in the main manuscript (see Figure 4 and Table 3). The forest plot below presents the results of the subgroup analysis for this outcome.


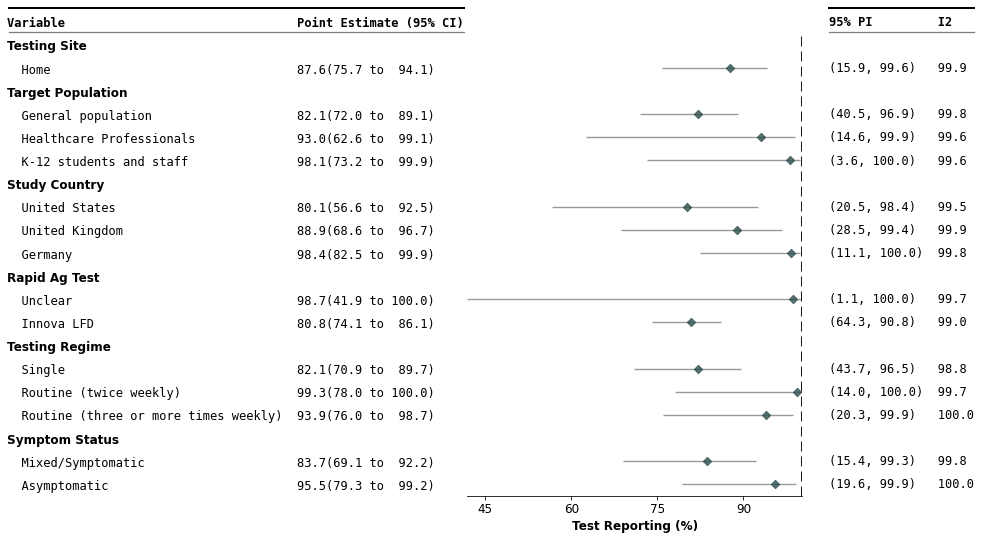


***Caption:*** CI = Confidence interval; I^2^ = Inconsistency index; PI = Prediction interval; n = number of tests.

The studies included in each of the subgroups are shown in the table below.

| **Variable** | | **Studies included** |
| --- | --- | --- |
| Testing Site | Home | Agusti, C., et al. [24], Chan, H.K., et al. [26], Downs, L.O., et al. [30], Engels, G., et al. [32], Herbert, C., et al. [35], Hoehl, S., et al. [38], Iftner, T., et al. [41], Kiene, S.M., et al. [43], Kwan, T.H., et al. [46], Love, N.K., et al. [49], Love, N.K., et al. [50], McDaniels-Davidson, C., et al. [52], Nodora, J.N., et al. [56], Soni, A., et al. [62], Stemler, J., et al. [63], Stohr, J.J.J.M., et al. [64], UK Health Security Agency [69] |
| Target Population | General population | Herbert, C., et al. [35], Kwan, T.H., et al. [46], Love, N.K., et al. [49], Love, N.K., et al. [50], Nodora, J.N., et al. [56], Soni, A., et al. [62], Stemler, J., et al. [63], Stohr, J.J.J.M., et al. [64], Nacov, J. A. et al. [79] |
|  | Healthcare Professionals | Agusti, C., et al. [24], Downs, L.O., et al. [30], Iftner, T., et al. [41], UK Health Security Agency [69] |
|  | K-12 students and staff | Agusti, C., et al. [24], Engels, G., et al. [32], Hoehl, S., et al. [38], Kiene, S.M., et al. [43], McDaniels-Davidson, C., et al. [52], Pudasaini, S., et al. [58] |
| Study country | United States | Herbert, C., et al. [35], Kiene, S.M., et al. [43], McDaniels-Davidson, C., et al. [52], Nodora, J.N., et al. [56], Soni, A., et al. [62] |
|  | United Kingdom | Downs, L.O., et al. [30], Hirst, J.A., et al. [37], Love, N.K., et al. [49], Love, N.K., et al. [50], UK Health Security Agency [69] |
|  | Germany | Engels, G., et al. [32], Hoehl, S., et al. [38], Iftner, T., et al. [41], Pudasaini, S., et al. [58], Stemler, J., et al. [63], Nacov, J. A. et al. [79] |
| Rapid Ag Test | Unlcear | Kwan, T.H., et al. [46], McDaniels-Davidson, C., et al. [52], Pudasaini, S., et al. [58], UK Health Security Agency [69] |
|  | Innova LFD | Downs, L.O., et al. [30], Hirst, J.A., et al. [37], Love, N.K., et al. [49], Love, N.K., et al. [50] |
| Testing Regime | Single | Agusti, C., et al. [24], Iftner, T., et al. [41], Stemler, J., et al. [63], Stohr, J.J.J.M., et al. [64], Nacov, J. A. et al. [79] |
|  | Routine (twice weekly) | Downs, L.O., et al. [30], Engels, G., et al. [32], Herbert, C., et al. [35] |
|  | Routine (three or more times weekly) | Hoehl, S., et al. [38], Kwan, T.H., et al. [46], Love, N.K., et al. [49], Love, N.K., et al. [50], Pudasaini, S., et al. [58], Soni, A., et al. [62], UK Health Security Agency [69] |
| Symptom Status | Mixed / Symptomatic | Agusti, C., et al. [24], Chan, H.K., et al. [26], Herbert, C., et al. [35], Hirst, J.A., et al. [37], Hoehl, S., et al. [38], Kiene, S.M., et al. [43], Kwan, T.H., et al. [46], McDaniels-Davidson, C., et al. [52], Nodora, J.N., et al. [56], Pudasaini, S., et al. [58], Stemler, J., et al. [63], Stohr, J.J.J.M., et al. [64], UK Health Security Agency [69], Nacov, J. A. et al. [79] |
|  | Asymptomatic | Downs, L.O., et al. [30], Engels, G., et al. [32], Iftner, T., et al. [41], Love, N.K., et al. [49], Love, N.K., et al. [50], Soni, A., et al. [62] |

In addition to the studies listed in the table above, the study “Temporal trends in test-seeking behaviour during the COVID-19 pandemic” by Eales et al. (https://doi.org/10.1101/2024.06.06.24308566) assessed the acceptance of COVID-19 RT-PCR tests and antigen-based self-tests through a population-wide survey in Australia between November 2021 and September 2023. While we could not extract results in absolute values (as required for our meta-analysis), it should be mentioned that Eales et al., based on the survey, estimated between ca. 50% and ca. 93% of positive results from COVID-19 antigen-detecting self-tests to be reported to official entities through an online portal.

# Text S16 – Results: COVID-19 self-testing time to diagnosis

C19ST was utilized for diagnosis of SARS-CoV-2 in 11 data sets [25, 27, 31, 51, 66, 67, 78, 79], but the criteria for defining a diagnosis varied across studies. In one dataset, C19ST was used only in individuals with symptoms and/or a known exposure [67], while in the remaining 10, asymptomatic individuals without a known contact history were also eligible for diagnosis based on C19ST results [25, 27, 31, 51, 66, 78, 79]. C19ST was also used to guide self-isolation decisions in 20 data sets [24, 37, 43, 45, 47, 49-51, 53, 54, 59, 65, 67, 69, 71, 74]. Reported turnaround times for C19ST ranged from “a few minutes” [24] and “almost immediate results” [74] to 30 minutes [37, 58, 73], enabling timely isolation or diagnostic decisions following testing.

# Table S1 – List of studies excluded

Please see the Excel file ‘Multimedia Component 2’ under <https://doi.org/10.1016/j.eclinm.2026.103838>.

# Table S2 – Results: Impact of C19ST on virus transmission, morbidity and mortality

| **Study** | **Study description** | **Impact on virus transmission, morbidity, and mortality** |
| --- | --- | --- |
| Du, Z., et al. [31] | Study retrospectively assessed the implementation of mass asymptomatic C19ST efforts in Hong Kong between 02/2022 and 03/2022. They projected state wide proportion of SARS-CoV-2 cases and mortality during this time using public health data. | - **Deaths averted**: In Hong Kong, from the day of mass C19ST implementation to the next day, SARS-CoV-2 case fatality ratio was estimated to decrease from 0.5% (Feb 25 2022) to 0.3% (Feb 26 2022). However, this decrease was likely more due to C19ST leading to an over-night increase in the cases detected than a reduction in SARS-CoV-2 deaths. |
| Hu, Y., et al. [40] | Study took place during 11/2022 in Chongqing, China. 17,655 residents of a university campus were required to self-isolate for 18 days and to perform C19ST three times a day and RT-PCR testing daily. The comparator group consisted of individuals living in the same city but outside of the medical campus, who did not receive the C19ST / RT-PCR testing intervention. | - **Secondary cases:** Individuals who tested positive on C19ST / RT-PCR testing were in close contact with 57 persons outside of the intervention. 11 of those contacts also became infected („secondary cases“; secondary attack proportion of 19.3%). Outside of the campus, 1,225 out of 2,268 close contacts of cases became infected (secondary attack proportion of 53.6%). |
| Koirala, A., et al. [45] | Study took place from 11/2021 to 12/2021 in New South Wales, Australia. In the intervention group, school pupils were allowed to still visit school despite a recent close contact with a SARS-CoV-2 case if they were asymptomatic, had a negative RT-PCR test (on day 0, 6, and 10-12 after being notified of being a close contact) and tested negative on daily C19ST. In the comparator group, individuals had to self-isolate instead of performing C19ST. | - **Secondary cases:** In the schools allowing C19ST instead of self-isolation, 2,852 individuals conducted C19ST after close contact with a case. These 2,852 individuals infected another 96 people (secondary attack rate of 3.4% [95% CI 2.7–4.1]). In the comparator group, 4,961 individuals had been in close contact with a case and were required to self-isolate. These 4,961 individuals infected another 141 people (secondary attack rate of 2.8% [95% CI 2.4–3.3]).* |
| Soni, A., et al. (a) [61] | Study took place from 6/2021 to 8/2021 in the United States. 500,000 free, rapid antigen tests for C19ST were distributed in about 40% of a populated area. The comparator group were the remaining 60% of that area where no C19ST were distributed. The entire area encompassed 226,038 individuals (as of 2019). | - **Cases averted:** The proportion of cases between the intervention and comparator group was reported to not differ significantly during the intervention period. However, two months after the intervention period, the study reported a statistically significant lower number of SARS-CoV-2 cases in the intervention compared to the control group. Using mathematical modeling, this difference was estimated to lead to ca. 40 cases averted per day during the post-intervention period due to C19ST. |
| Tulloch, J. S. P., et al. [68] | Study was conducted from 12/2020 to 01/2021 in the United Kingdom. Staff from 11 care-homes were offered twice-weekly self-testing as routine prevention measure. 81.7% of the 498 staff conducted C19STs, but only 8.6% of those showed high adherence to the twice weekly testing scheme. The comparator group were 71 care-homes similar to the study care-homes in the same city. | - **Incidence:** During the study period, there was no meaningful difference in the proportion of study versus comparator care-homes experiencing outbreaks (54.5% [95% CI 23.4– 83.3%; 6/11] and 36.6% [95% CI 25.5– 48.9%; 26/71], respectively). |
| Stirrup, O. et al. [81] | Study took place from 01/2023 to 08/2023, including 83 care-homes across the United Kingdom. 42 care-homes were randomized to a control group, with staff self-testing in case of symptoms or in the event of outbreaks, as per national guidelines during this time. In addition to the measures in the intervention group, in the 43 intervention care-homes, staff voluntary self-tested twice-weekly. Also, improved corporate communication strategies and support for sick pay were offered. | - **Hospitalisation**: In the intervention group, incidence rate of all-cause hospital admissions per 1,000 care-home resident person-years was 367.3 (204 events), versus 506.6 (293 events) in the control group. - **Deaths averted**: In the intervention group, incidence rate of COVID-19 mortality in residents per 1,000 care-home resident person-years was 5.4 (3 events) and the incidence rate of all-cause mortality in residents per 1,000 care-home resident person-years was 243.4 (136 events). In the control group, these values were 8.4 (5 events) and 225.9 (134 events), respectively.   *Note*: One care-home in the control and two care-homes in the intervention group dropped out before any outcome data was collected. The trial was terminated early as it was not sufficiently powered to assess the intervention’s impact. |

**Additional studies not included in the systematic review**

In addition to the studies listed in the table above, the study “Index Cases First Identified by Nasal-Swab Rapid COVID-19 Tests Had More Transmission to Household Contacts Than Cases Identified by Other Test Types” by Jenny Ji et al. (https://doi.org/ 10.1371/journal.pone.0292389) compared SARS-CoV-2 transmission in households where the index case was identified through C19ST with households where the index case was identified through other means of SARS-CoV-2 testing (mainly RT-PCR). While this study included too few study participants conducting C19ST (n = 28) to be included in our systematic review, it should be mentioned that Ji et al. found a secondary attack proportion of 53.6% (95% CI 38.8–68.3%) in households where the index case was identified by C19ST, compared to a secondary attack proportion of 27.2% 95% CI 19.5– 35.0% when utilizing any other test type for index-test detection. In addition, the study “A Randomized Trial of At-Home COVID-19 Tests, Telemedicine, and Rapid Prescription Delivery for Immunocompromised Individuals” by Vogel et al. (https://doi.org/10.1016/j.mayocpiqo.2025.100627) assessed the impact of an intervention package entailing C19ST, telemedicine, and early prescription of Paxlovid on intensive care unit admission and overall hospitalization in immunocompromised individuals. It suggested that C19ST combined with early Paxlovid treatment in immunocompromised individuals may reduce intensive care unit admission, though not overall hospitalization. This study could not be included in the main analysis, as the majority of the study duration (December 1, 2022 to May 16, 2024) was outside of the time frame relevant for our systematic review (March 11, 2020 to May 5, 2023).

* These values differ from those presented in the manuscript of Koirala et al. [45], but have been confirmed as the correct ones by the study author.

# Table S3 – Results: Linkage after positive COVID-19 self-test result

| **Study**  **author**  **[reference to study]** | **ID of data sets included** | **Study characteristics** | | **Action after positive C19ST result** |
| --- | --- | --- | --- | --- |
|  |  | Study  location | Target population |  |
| Agusti, C., et al. [24] | 24_1 and 24_2 | Spain | Healthcare Professionals | Self-isolate; contact general practitioner as soon as possible |
| Bresser, M., et al. [25] | 25_0 | Zambia | General population | Follow national COVID-19 guidelines |
| Chan, H.K. et al. [26] | 26_0 | Malaysia | Manufacturing employees | Report test result to employer; follow national COVID-19 guidelines |
| Chong, S.J. et al. [28] | 28_1, 28_2, 28_3, and 28_4 | Singapore | General population | Notify government authorities (visit specific website to understand exact steps) |
| Downs, L. O., et al. [30] | 30_0 | United Kingdom | Healthcare Professionals | RT-PCR confirmatory testing (unclear whether this was compulsory or not) |
| Gorgels, K.M.F.  et al. [33] | 33_0 | Netherlands | General population | RT-PCR confirmatory testing (compulsory; as per national COVID-19 guidelines) |
| Harmon, A. et al. [34] | 34_0 | United States | General population | RT-PCR confirmatory testing or professional Ag-RDT (conducting either one was compulsory); do not go to school |
| Herbert, C., et al. (a) [35] | 35_1 and 35_2 | United States | General population | RT-PCR confirmatory testing (compulsory; using at-home collection kit); repeat antigen test on the same day or after 1 day; care directed by participant's primary or urgent care provider |
| Hirst, J. A., et al. [37] | 37_1 and 37_2 | United Kingdom | University students and staff | RT-PCR confirmatory testing (compulsory); self-isolate |
| Kiene, S.M. et al. [43] | 43_0 | United States | K-12 students and staff | Self-isolate |
| Koirala, A. et al. [45] | 45_0 | Australia | K-12 students and staff | RT-PCR confirmatory testing (compulsory); self-isolate |
| Lamb, G., et al. [47] | 47_0 | United Kingdom | Healthcare Professionals | RT-PCR confirmatory testing (compulsory); self-isolate |
| Love, N.K., et al. (a) [49] | 49_0 | United Kingdom | General population | RT-PCR confirmatory testing (compulsory); self-isolate |
| Love, N.K., et al. (b) [50] | 50_0 | United Kingdom | General population | RT-PCR confirmatory testing (voluntary); self-isolate |
| Marbán‐Castro, E.  et al. [51] | 51_1 and 51_2 | Georgia | Healthcare Professionals; K-12 students and staff | Self-isolate (as part of following national COVID-19 guidelines); report own result and those of household-members to study staff |
| McDaniels-Davidson,  C. et al. [52] | 52_0 | United States | K-12 students and staff | RT-PCR confirmatory testing or a second C19ST (conducting either one was compulsory) |
| Moonan, P.K. et al. [53] | 53_0 | United States | General population | Self-isolate (as part of following US CDC COVID-19 guidelines; for 10 days when testing positive during 1/1/21-12/31/21 and for 5 days when testing positive during 1/1/22-3/31/22) |
| Nagasawa M. et al. [54] | 54_0 | Japan | Healthcare Professionals | Self-isolate |
| Nakgul, L. et al. [55] | 55_0 | Thailand | K-12 students and staff | RT-PCR confirmatory testing (compulsory); in case of confirmed case the entire class is closed for one week (as per government guidelines) |
| Nodora, J.N. et al. [56] | 56_0 | United States | General population | Follow national COVID-19 guideline |
| Ryan, F. et al. [59] | 59_0 | United Kingdom | Healthcare Professionals | RT-PCR confirmatory testing (compulsory); self-isolate (at home for 10 days as per UK Government requirements at the time); do not attend work / immediately leave the workplace |
| Smit, T. et al. [60] | 60_0 | Netherlands | General population | RT-PCR confirmatory testing (unclear whether this was compulsory or not) |
| Soni, A. et al. (a) [61] | 61_0 | United States | General population | Stop testing individual with positive result and give remaining test to other household members; encouraged to report result to health authority |
| Soni, A. et al. (b) [62] | 62_0 | United States | General population | Contact health-care provider for any medical questions; continue routine testing |
| Tinker, S.C., et al. [65] | 65_0 | United States | University students and staff | RT-PCR confirmatory testing (compulsory); self-isolate |
| Tsang, N.N.Y. et al. [66] | 66_0 | Hong Kong | General population | Report test result to study team and exit study |
| Tsao, J. et a l. [67] | 67_0 | United States | University students and staff | If asymptomatic: RT-PCR confirmatory testing (compulsory);  If symptomatic: self-isolate (considered "presumed positive") |
| UK Health Security Agency [69] | 69_0 | United Kingdom | Healthcare Professionals | RT-PCR confirmatory testing (compulsory); self-isolate (until RT-PCR result available and when positive); identify close contacts and offer daily testing or self-isolation |
| U.o.Liverpool [70] | 70_1 | United Kingdom | General population | RT-PCR confirmatory testing (voluntarily) |
| Wachinger, J., et al. [71] | 71_1, 71_2 | Germany | K-12 students and staff | Self-isolate; notify school and local public health authority |
| Willeit, P., et al. [72] | 72_1, 72_2, and 72_3 | Austria | K-12 students and staff | Leave school immediately and refer to local health authorities |
| Wong, S.C. et al. [73] | 73_0 | Hong Kong | Healthcare Professionals | Perform another C19ST; if repeated test is also positive: perform RT-PCR confirmatory testing (compulsory) and be interviewed by infection control nurse |
| Wu, S., et al. [74] | 74_0 | Singapore | Healthcare Professionals | RT-PCR confirmatory testing (compulsory); self-isolate; report result to reporting officer and occupational health clinic |
| Yun, G. et al. [75] | 75_1, 75_2, 75_3, and 75_4 | Republic  of Korea | K-12 students and staff | RT-PCR confirmatory testing or professional Ag-RDT (conducting either one was compulsory); do not go to school |
| Mwangoka, G.W. et al. [78] | 78_0 | Tanzania | General population | Education of COVID-19 interventions (self-isolation) and provision of masks and sanitizers for those who tested positive; further referral to healthcare facilities if also symptomatic |

Ag-RDT = antigen rapid diagnostic test; C19ST = COVID-19 self-testing; RT-PCR = reverse transcriptase polymerase chain reaction; US CDC = United States Centers for Disease Control and Prevention.

# Table S4 – Results: Linkage after negative COVID-19 self-test result

| **Study author**  **[reference to study]** | **ID of data sets included** | **Study characteristics** | | **Action after positive C19ST result** |
| --- | --- | --- | --- | --- |
|  |  | Study location | Target population |  |
| Chong, S.J. et al. [28] | 28_1, 28_2, 28_3, and 28_4 | Singapore | General population | Leave self-isolation and continue daily activities; repeat C19ST with the next testing interval; notify authorities if test was taken on last day of isolation period (day 7) |
| Harmon, A. et al. [34] | 34_0 | United States | General population | Continue work; repeat C19ST with next testing interval |
| Herbert, C., et al. (a) [35] | 35_1 | United States | General population | Continue to test twice-weekly until end of 12-week period |
| Herbert, C., et al. (a) [35] | 35_2 | United States | General population | Continue to test twice-weekly until end of 2-week period; then continue weekly symptom survey |
| Hirst, J. A., et al. [37] | 37_1 and 37_2 | United Kingdom | University students and staff | Continue to follow COVID-19 rules |
| Hogg, C. et al. [39] | 39_0 | United Kingdom | Healthcare Professionals | Continue work |
| Kiene, S.M. et al. [43] | 43_0 | United States | K-12 students and staff | Continue work / school |
| Koirala, A. et al. [45] | 45_0 | Australia | K-12 students and staff | Exemption from self-isolation if also asymptomatic and previous RT-PCR tests were negative |
| Love, N.K., et al. (a) [49] | 49_0 | United Kingdom | General population | Exemption from self-isolation for 24h; repeat testing with the next interval |
| Love, N.K., et al. (b) [50] | 50_0 | United Kingdom | General population | Exemption from self-isolation for 24 hours, then test again |
| Marbán‐Castro, E.  et al. [51] | 51_1 and 51_2 | Georgia | Healthcare Professionals; K-12 students and staff; general population | Continue work / school; repeat C19ST after three days if at the time of the negative results, symptoms were also present; report own result and those of household-members to study staff |
| Nagasawa M. et al. [54] | 54_0 | Japan | Healthcare Professionals | Return to work either three days after contact or 5 days after infection (given that the person testing negative is also fever-free for 14h) |
| Nakgul, L. et al. [55] | 55_0 | Thailand | K-12 students and staff | Repeat C19ST with next testing interval |
| Pudasaini, S. et al. [58] | 58_0 | Germany | K-12 students and staff | Continue school music trip |
| Ryan, F. et al. [59] | 59_0 | United Kingdom | Healthcare Professionals | Continue work; repeat C19ST with next testing interval |
| Smit, T. et al. [60] | 60_0 | Netherlands | General population | Random sample of symptomatic individuals were to send in nose + throat swab for RT-PCR testing despite negative self-test result |
| Soni, A. et al. (a) [61] | 61_0 | United States | General population | Continue at-home testing |
| Soni, A. et al. (b) [62] | 62_0 | United States | General population | Repeat C19ST with next testing interval |
| Tinker, S.C., et al. [65] | 65_0 | United States | University students and staff | Continue college life; repeat testing with the next interval |
| Tsang, N.N.Y. et al. [66] | 66_0 | Hong Kong | General population | Report result to study team; repeat C19ST with next testing interval |
| UK Health Security Agency [69] | 69_0 | United Kingdom | Healthcare Professionals | Continue work; repeat C19ST with next testing interval |
| U.o.Liverpool [70] | 70_2 | United Kingdom | Healthcare Professionals | Exemption from self-isolation for 24 hours (go to work); retest with the next interval |
| Wachinger, J., et al. [71] | 71_1 and 71_2 | Germany | K-12 students and staff | Go to school / send child to school; retest with the next interval |
| Willeit, P., et al. [72] | 72_1, 72_2, and 72_3 | Austria | K-12 students and staff | Stay in class; repeat testing with the next interval |
| Wong, S.C. et al. [73] | 73_0 | Hong Kong | Healthcare Professionals | Continue work; repeat C19ST with next testing interval |
| Yun, G. et al. [75] | 75_1, 75_2, 75_3, and 75_4 | Republic of Korea | K-12 students and staff | Resume school; repeat testing with the next interval and after contact |
| Davies, M. et al. [76] | 76_0 | United Kingdom | Unclear | Continue participating in training and competitions; continue work |
| Mwangoka, G.W. et al. [78] | 78_0 | Tanzania | General population | Either receive COVID-19 vaccination or be advised to do so |

Ag-RDT = antigen rapid diagnostic test; C19ST = COVID-19 self-testing; RT-PCR = reverse transcriptase polymerase chain reaction.

# Table S5 – Results: Behaviour changes due to C19ST

| **Study** | **Study description** | **Behaviour changes** |
| --- | --- | --- |
| Kiene, S. M. et al. [43] | Study was conducted from 11/2021 to 03/2022 in the United States. 2,285 middle school students and staff from three different schools were included, with one school (n = 761 students and staff) offering C19ST, while in the other two schools (n = 1,524 students and staff) COVID-19 testing was conducted on-site by trained staff. | - School staff showed higher testing participation and adherence to the testing schedule under at-home C19ST (0.286 [SE 0.040] and 0.470 [SE 0.054], respectively) compared to professional testing on-site (0.132 [SE 0.021] and 0.222 [SE 0.029], respectively). - For school students, participation and adherence rates were similar for at-home C19ST (0.091 [SE 0.012] and 0.380 [SE 0.036], respectively) and receiving testing by trained staff (0.105 [SE 0.012] and 0.400 [SE 0.030], respectively). |
| Love, N.K., et al. (a) [49] | Study was conducted from 04/2021 to 07/2021 in the United Kingdom. Asymptomatic contacts of confirmed SARS-CoV-2 cases were offered daily C19ST for seven days instead of 10 days of self-isolation. 26,123 of the 28,757 eligible contacts consented to daily self-testing. The comparator group consisted of 23,500 individuals that self-isolated. | - The study observed that 82% of the study participants in the self-isolation group and 84% in the C19ST subgroup with a positive self-test result significantly reduced contact with non-household members. Of those individuals performing C19ST and receiving only negative results, only 57% reported reduced contact, with 11% reporting increased contact. - The study suggests that individuals receiving negative antigen test results may perceive themselves as less infectious, leading to greater social interaction, whereas positive C19STs lead to self-isolation |
| Love, N.K. et al. (b) [50] | Study was conducted from 12/2020 to 01/2021 in the United Kingdom. Asymptomatic contacts of confirmed SARS-CoV-2 cases were offered daily COVID-19 self-testing (C19ST) instead of self-isolation. 882 of the 1,760 eligible contacts consented to daily self-testing. The comparator group consisted of all contacts reported to the National Health Service during the study period with the same inclusion criteria. | - 84 individuals who had chosen daily C19ST instead of self-isolation were eventually confirmed for a SARS-CoV-2 infection. 64 of those reported that they had been in contact with other individuals prior to this diagnosis, which they would not have been if they had strictly self-isolated. |
| Moonan, P. K., et al. [53] | Study was conducted from 01/2021 to 03/2022 in the United States. A representative sample of the US population > 18 years, who was diagnosed with SARS-CoV-2 by any means of testing, was interviewed about their behavior after receiving the SARS-CoV-2 diagnosis. A total of 15,923 individuals were included. | - Compared to individuals who received a COVID-19 diagnosis through testing by trained staff, individuals who conducted C19ST were less likely to self-isolate (aOR 0.72 [95% CI 0.57–0.90]), had a lower chance of being compliant with local guidelines if they were isolating (aOR 0.71 [95% CI 0.53–0.95]), and were less likely to inform their contacts about their SARS-CoV-2 diagnosis (aOR 0.79 [95% CI 0.53–1.18]). |
| Wachinger, J., et al. [71] | Study was conducted from 03/2021 to 05/2021 in Germany. During this period, school pupils and staff were offered thrice weekly C19ST to voluntarily be conducted at home. 109 of the 186 school pupils and 21 of the 34 staff participated in C19ST. The behavior of these individuals was compared to their behavior prior to implementing C19ST. | - A riskier behaviour, e.g., less mask wearing than prior to implementing C19ST, compared to before the implementation of self-testing was not reported by any of the study participants |
| Qasmieh, S.A., et al. [80] | Study was conducted from 03/2022 and 10/2023, surveying adults across the United States about their COVID-19 testing behaviour and other COVID-19 related aspects (e.g., vaccination status). A total of 1,918 individuals with symptoms of acute respiratory illness were included in the survey, with 623 of those reporting having self-tested. | - Of the people who tested positive on C19ST and had symptoms of acute respiratory illness (n=184), 47% (n=86) turned towards healthcare providers - Of the people who tested negative on C19ST and had symptoms of acute respiratory illness (n=313), 30% (n=94) turned to healthcare providers |

# Table S6 – Results: COVID-19 self-testing resource usage

| **Study** | **Study description** | **Resource usage** |
| --- | --- | --- |
| Soni, A. et al. (b) [62] | Study was conducted from 10/2021 to 02/2022 in the United States. Individuals from the general population were asked to perform C19ST and a molecular confirmatory-testing at home every other day over a 15-day period. Self-tests were shipped to a total of 7,303 individuals, with 6,711 of those reporting at least one test result. | - About 556 staff hours were required throughout the study to coordinate test distribution and performance of C19ST and molecular confirmatory-testing with study participants. |
| UK Health Security Agency [69] | Study was conducted from 01/2021 to 02/2021 in the United Kingdom. 138 healthcare workers at National Health Services trusts were offered 7 days of daily C19ST instead of self-isolation after close contact to a confirmed SARS-CoV-2 case. 111 healthcare workers participated, performing a total of 719 C19ST. | - Study staff reported a high burden for initial setup. The costs of daily C19ST, including program set-up costs, were estimated to be USD 105 (range: USD 52-219) per potential work absence day averted. |
| Wu, S. et al. [74] | Study was conducted from 07/2021 to 09/2021 in Singapore. 8,000 hospital staff had to conduct C19ST twice weekly as long as asymptomatic, and to take a RT-PCR test when symptomatic. Over the entire course of the study, 156,000 self-tests were distributed to all staff, with 65,500 of those self-tests distributed during the first two weeks of the study. | - When distributing 65,500 self-tests to 8,000 staff during the first two weeks of the study, the per-week costs of C19ST was estimated to be USD 113,668. |
| Davies, M. et al. [76] | Study took place from 06/2021 to 08/2021 in the United Kingdom. Sport athletes and staff after low-risk contact with a confirmed SARS-CoV-2 case were required to conduct daily COVID-19 self-testing for up to seven days after exposure. If self-tests remained negative, individuals were allowed to participate in sport training and competition as well as to continue working. | - Implementing C19ST for individuals with low-risk contact (among other testing interventions) was described as labour-intensive by study authors. |

# Figure S1 – Results: Summary of the quality assessment


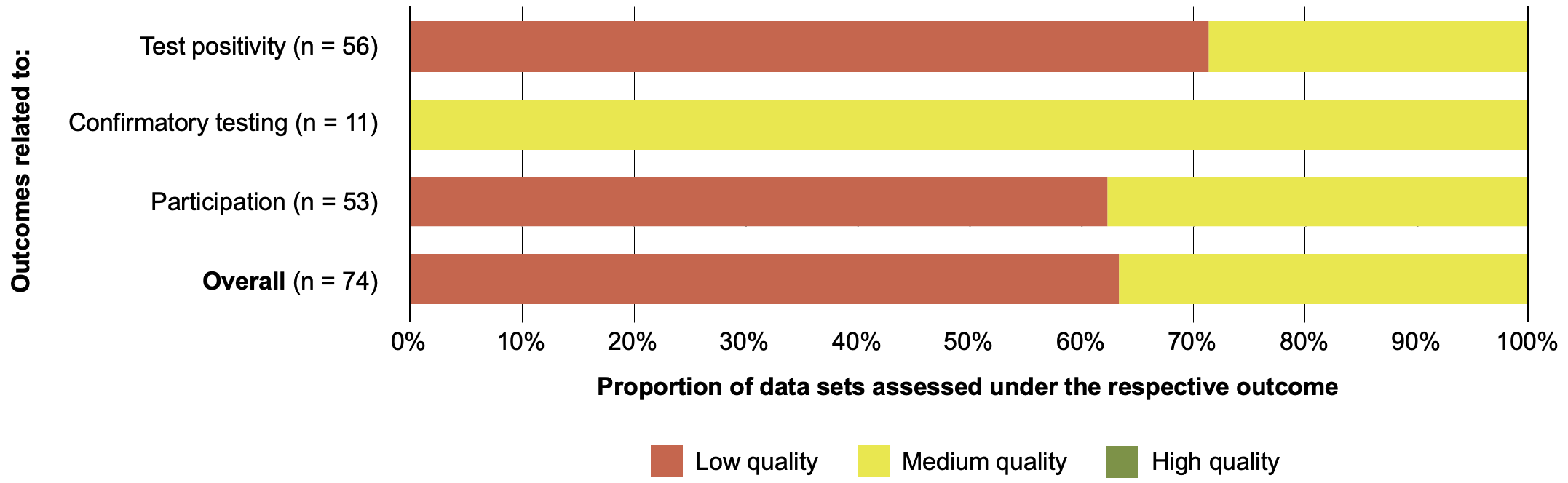


***Caption:*** We assessed the quality of all data sets included in the meta-analysis, utilizing the revised Joanna Briggs Institute (JBI) Critical Appraisal Tool for Quasi-Experimental Studies [16]. Given the heterogeneity of outcomes, tailored interpretation guides were developed for each category of meta-analysed outcomes, assessed individually, and then synthesized collectively (Text S4).

# Sources

To provide a clear link between the studies included in the systematic review and the review’s results presented in the main manuscript, we’ve used the same reference number for the studies cited in the supplements as in the main manuscript.

11. Page MJ, McKenzie JE, Bossuyt PM, Boutron I, Hoffmann TC, Mulrow CD, et al. The PRISMA 2020 statement: an updated guideline for reporting systematic reviews. BMJ. 2021;372:n71.

16. Barker TH, Habibi N, Aromataris E, Stone JC, Leonardi-Bee J, Sears K, et al. The revised JBI critical appraisal tool for the assessment of risk of bias for quasi-experimental studies. JBI Evid Synth. 2024;22(3):378-88.

18. Lin L, Chu H. Meta-analysis of Proportions Using Generalized Linear Mixed Models. Epidemiology. 2020;31(5):713-7.

20. Hunter JP, Saratzis A, Sutton AJ, Boucher RH, Sayers RD, Bown MJ. In meta-analyses of proportion studies, funnel plots were found to be an inaccurate method of assessing publication bias. J Clin Epidemiol. 2014;67(8):897-903.

21. exchange-rates.org. Live Currency Exchange Converter. 2025. Available from: https://www.exchange-rates.org. Accessed on: December 20th, 2025.

22. World Bank Group. GDP deflator. 2025. Available from: https://data.worldbank.org/indicator/NY.GDP.DEFL.ZS. Accessed on: December 20th, 2025.

23. Viechtbauer W. Conducting meta-analyses in R with the metafor package. Journal of statistical software. 2010;36:1-48.

24. Agusti C, Martinez-Riveros H, Gonzalez V, Fernandez-Rivas G, Diaz Y, Montoro-Fernandez M, et al. Feasibility of an online antigen self-testing strategy for SARS-CoV-2 addressed to health care and education professionals in Catalonia (Spain). The TESTA'T- COVID Project. PLoS One. 2022;17(9):e0275006.

25. Bresser M, Erhardt RM, Shanaube K, Simwinga M, Mahlatsi PA, Belus J, et al. Evaluation of COVID-19 antigen rapid diagnostic tests for self-testing in Lesotho and Zambia. PLoS One. 2024;19(2):e0280105.

26. Chan HK, Marban-Castro E, Rahman SA, Sem X, Zulkifli NF, Redzuan S, et al. Implementation pilot study of community self-testing for COVID-19 among employees of manufacturing industries and their household members in 2022 to 2023. PLOS Glob Public Health. 2024;4(6):e0003269.

27. Chen SH, Wu JL, Liu YC, Yen TY, Lu CY, Chang LY, et al. Differential clinical characteristics and performance of home antigen tests between parents and children after household transmission of SARS-CoV-2 during the Omicron variant pandemic. Int J Infect Dis. 2023;128:301-6.

28. Chong SJ, Seah BZ, Jailani RI, Angeles DC, Chong XY, Lee JH. Assessment of COVID-19 Positive Rates Amongst COVID-19 Close Contacts Through the Health Risk Warning System. J Med Syst. 2023;47(1):54.

29. D'Agostino EM, Corbie G, Kibbe WA, Hornik CP, Richmond A, Dunston A, et al. Increasing access and uptake of SARS-CoV-2 at-home tests using a community-engaged approach. Prev Med Rep. 2022;29:101967.

30. Downs LO, Eyre DW, O'Donnell D, Jeffery K. Home-based SARS-CoV-2 lateral flow antigen testing in hospital workers. J Infect. 2021;82(2):282-327.

31. Du Z, Tian L, Jin DY. Understanding the impact of rapid antigen tests on SARS-CoV-2 transmission in the fifth wave of COVID-19 in Hong Kong in early 2022. Emerg Microbes Infect. 2022;11(1):1394-401.

32. Engels G, Forster J, Streng A, Rucker V, Rudolph P, Pietsch F, et al. Acceptance of Different Self-sampling Methods for Semiweekly SARS-CoV-2 Testing in Asymptomatic Children and Childcare Workers at German Day Care Centers: A Nonrandomized Controlled Trial. JAMA Netw Open. 2022;5(9):e2231798.

33. Gorgels KMF, van Iersel S, Keijser SFA, Hoebe C, Wallinga J, van Hoek AJ. Estimating infection prevalence using the positive predictive value of self-administered rapid antigen diagnostic tests: An exploration of SARS-CoV-2 surveillance data in the Netherlands from May 2021 to April 2022. PLoS One. 2024;19(2):e0298218.

34. Harmon A, Chang C, Salcedo N, Sena B, Herrera BB, Bosch I, et al. Validation of an At-Home Direct Antigen Rapid Test for COVID-19. JAMA Netw Open. 2021;4(8):e2126931.

35. Herbert C, Kheterpal V, Suvarna T, Broach J, Marquez JL, Gerber B, et al. Design and Preliminary Findings of Adherence to the Self-Testing for Our Protection From COVID-19 (STOP COVID-19) Risk-Based Testing Protocol: Prospective Digital Study. JMIR Form Res. 2022;6(6):e38113.

36. Herbert C, Shi Q, Kheterpal V, Nowak C, Suvarna T, Durnan B, et al. Use of a Digital Assistant to Report COVID-19 Rapid Antigen Self-test Results to Health Departments in 6 US Communities. JAMA Netw Open. 2022;5(8):e2228885.

37. Hirst JA, Logan M, Fanshawe TR, Mwandigha L, Wanat M, Vicary C, et al. Feasibility and Acceptability of Community Coronavirus Disease 2019 Testing Strategies (FACTS) in a University Setting. Open Forum Infect Dis. 2021;8(12):ofab495.

38. Hoehl S, Schenk B, Rudych O, Gottig S, Foppa I, Kohmer N, et al. High-Frequency Self-Testing by Schoolteachers for Sars-Cov-2 Using a Rapid Antigen Test-Results of the Safe School Hesse study. Dtsch Arztebl Int. 2021;118(14):252-3.

39. Hogg C, Boots S, Howorth D, Williams C, Heginbothom M, Salmon J, et al. Test performance of lateral flow rapid antigen tests for COVID-19 in Welsh adult care home staff using routine surveillance data. PLoS One. 2023;18(8):e0290406.

40. Hu Y, Peng B, Fan J, Yang Z, Xue J, Long Q, et al. Efficacy of rapid antigen self-testing for SARS-CoV-2 screening: Real-world evidence from a prospective cohort study. Genes Dis. 2024;11(5):101151.

41. Iftner T, Iftner A, Pohle D, Martus P. Evaluation of the specificity and accuracy of SARS-CoV-2 rapid antigen self-tests compared to RT-PCR from 1015 asymptomatic volunteers. medRxiv [preprint]. 2022.

42. Kheiroddin P, Schoberl P, Althammer M, Cibali E, Wurfel T, Wein H, et al. Results of WICOVIR Gargle Pool PCR Testing in German Schools Based on the First 100,000 Tests. Front Pediatr. 2021;9:721518.

43. Kiene SM, McDaniels-Davidson C, Lin CD, Rodriguez T, Chris N, Bravo R, et al. At-Home Versus Onsite COVID-19 School-based Testing: A Randomized Noninferiority Trial. Pediatrics. 2023;152(Suppl 1).

44. Kim AE, Bennett JC, Luiten K, O'Hanlon JA, Wolf CR, Magedson A, et al. Comparative Diagnostic Utility of SARS-CoV-2 Rapid Antigen and Molecular Testing in a Community Setting. J Infect Dis. 2024;230(2):363-73.

45. Koirala A, Winkler N, Sharpe C, van Tussenbroek T, Wood P, Macartney K, et al. Real-world utilisation of SARS-CoV-2 rapid antigen testing to enable face-to-face learning in Australian schools, an ecological study. Aust N Z J Public Health. 2024;48(3):100159.

46. Kwan TH, Wong NS, Chan CP, Yeoh EK, Wong SY, Lee SS. Mass Screening of SARS-CoV-2 With Rapid Antigen Tests in a Receding Omicron Wave: Population-Based Survey for Epidemiologic Evaluation. JMIR Public Health Surveill. 2022;8(11):e40175.

47. Lamb G, Heskin J, Randell P, Mughal N, Moore LS, Jones R, et al. Real-world evaluation of COVID-19 lateral flow device (LFD) mass-testing in healthcare workers at a London hospital; a prospective cohort analysis. J Infect. 2021;83(4):452-7.

48. Lau CS, Aw TC. SARS-CoV-2 Antigen Testing Intervals: Twice or Thrice a Week? Diagnostics (Basel). 2022;12(5).

49. Love NK, Ready DR, Turner C, Verlander NQ, French CE, Martin AF, et al. Daily use of lateral flow devices by contacts of confirmed COVID-19 cases to enable exemption from isolation compared with standard self-isolation to reduce onward transmission of SARS-CoV-2 in England: a randomised, controlled, non-inferiority trial. Lancet Respir Med. 2022;10(11):1074-85.

50. Love NK, Ready DR, Turner C, Yardley L, Rubin GJ, Hopkins S, et al. The acceptability of testing contacts of confirmed COVID-19 cases using serial, self-administered lateral flow devices as an alternative to self-isolation. J Med Microbiol. 2022;71(8).

51. Marban-Castro E, Getia V, Alkhazashvili M, Japaridze M, Jikia I, Erkosar B, et al. Implementing a pilot study of COVID-19 self-testing in high-risk populations and remote locations: results and lessons learnt. BMC Public Health. 2024;24(1):511.

52. McDaniels-Davidson C, Arechiga-Romero M, Snyder T, Chris N, Sturgis K, Moore V, et al. Development of an At-Home COVID-19 Test Results-Reporting System for a School District Primarily Serving Underrepresented Minority Groups, San Diego, CA, 2021-2022. Am J Public Health. 2022;112(S9):S883-S6.

53. Moonan PK, Smith JP, Borah BF, Vohra D, Matulewicz HH, DeLuca N, et al. Home-Based Testing and COVID-19 Isolation Recommendations, United States. Emerg Infect Dis. 2023;29(9):1921-4.

54. Nagasawa M, Kato T, Sakaguchi H, Tanaka I, Watanabe M, Hiroshima Y, et al. Single-Facility Analysis of COVID-19 Status of Healthcare Employees during the Eighth and Ninth Pandemic Waves in Japan after Introducing Regular Rapid Antigen Testing. Vaccines (Basel). 2024;12(6).

55. Nakgul L, Pasomsub E, Thongpradit S, Chanprasertyothin S, Prasongtanakij S, Thadanipon K, et al. Saliva and wastewater surveillance for SARS-CoV-2 during school reopening amid COVID-19 pandemic in Thailand. Public Health Pract (Oxf). 2023;5:100378.

56. Nodora JN, Martinez ME, McDaniels-Davidson C, Shen J, Sitapati AM, Torriani F, et al. Distribution of COVID-19 Home Testing Through Community Health Centers: Results of the COVID CoNOce MAS Study. J Prim Care Community Health. 2024;15:21501319241259684.

57. Papenburg J, Campbell JR, Caya C, Dion C, Corsini R, Cheng MP, et al. Adequacy of Serial Self-performed SARS-CoV-2 Rapid Antigen Detection Testing for Longitudinal Mass Screening in the Workplace. JAMA Netw Open. 2022;5(5):e2210559.

58. Pudasaini S, Boldt KL, Hitzek J, Mockel L, Slagman A, Theuring S, et al. The Feasibility of School Music Trips With Safe Cohorts During the COVID-19 Pandemic. Dtsch Arztebl Int. 2022;119(31-32):542-3.

59. Ryan F, Cole-Hamilton J, Dandamudi N, Futschik ME, Needham A, Saquib R, et al. Faster detection of asymptomatic COVID-19 cases among care home staff in England through the combination of SARS-CoV-2 testing technologies. Sci Rep. 2024;14(1):7475.

60. Smit T, Carstens G, Han W, Bulsink K, de Bakker J, Elahi M, et al. Flexible and scalable participatory syndromic and virological surveillance for respiratory infections: our experiences in The Netherlands. PLoS One. 2024;20(8):e0303230.

61. Soni A, Herbert C, Baek J, Shi Q, Marquez J, Harman E, et al. Association of Mass Distribution of Rapid Antigen Tests and SARS-CoV-2 Prevalence: Results from NIH-CDC funded Say Yes! Covid Test program in Michigan. medRxiv [preprint]. 2022.

62. Soni A, Herbert C, Pretz C, Stamegna P, Filippaios A, Shi Q, et al. Design and implementation of a digital site-less clinical study of serial rapid antigen testing to identify asymptomatic SARS-CoV-2 infection. J Clin Transl Sci. 2023;7(1):e120.

63. Stemler J, Salmanton-Garcia J, Weise B, Tobben C, Joisten C, Fleig J, et al. A pilot surveillance report of SARS-CoV-2 rapid antigen test results among volunteers in Germany, 1st week of July 2022. Infection. 2023;51(2):465-9.

64. Stohr J, Zwart VF, Goderski G, Meijer A, Nagel-Imming CRS, Kluytmans-van den Bergh MFQ, et al. Self-testing for the detection of SARS-CoV-2 infection with rapid antigen tests for people with suspected COVID-19 in the community. Clin Microbiol Infect. 2022;28(5):695-700.

65. Tinker SC, Prince-Guerra JL, Vermandere K, Gettings J, Drenzik C, Voccio G, et al. Evaluation of self-administered antigen testing in a college setting. Virol J. 2022;19(1):202.

66. Tsang NNY, So HC, Cowling BJ, Leung GM, Ip DKM. Effectiveness of BNT162b2 and CoronaVac COVID-19 vaccination against asymptomatic and symptomatic infection of SARS-CoV-2 omicron BA.2 in Hong Kong: a prospective cohort study. Lancet Infect Dis. 2023;23(4):421-34.

67. Tsao J, Kussman AL, Costales C, Pinsky BA, Abrams GD, Hwang CE. Accuracy of Rapid Antigen vs Reverse Transcriptase-Polymerase Chain Reaction Testing for SARS-CoV-2 Infection in College Athletes During Prevalence of the Omicron Variant. JAMA Netw Open. 2022;5(6):e2217234.

68. Tulloch JSP, Micocci M, Buckle P, Lawrenson K, Kierkegaard P, McLister A, et al. Enhanced lateral flow testing strategies in care homes are associated with poor adherence and were insufficient to prevent COVID-19 outbreaks: results from a mixed methods implementation study. Age Ageing. 2021;50(6):1868-75.

69. United Kingdom Health Security Agency. An evaluation of the pilot of daily contact testing of healthcare workers in NHS acute hospital and ambulance trusts. London, United Kingdom; 2022.

70. University of Liverpool. Covid-SMART Asymptomatic Testing Pilot in Liverpool City Region: Quantitative Evaluation. Liverpool; 2021.

71. Wachinger J, Schirmer M, Tauber N, McMahon SA, Denkinger CM. Experiences with opt-in, at-home screening for SARS-CoV-2 at a primary school in Germany: an implementation study. BMJ Paediatr Open. 2021;5(1):e001262.

72. Willeit P, Bernar B, Zurl C, Al-Rawi M, Berghold A, Bernhard D, et al. Sensitivity and specificity of the antigen-based anterior nasal self-testing programme for detecting SARS-CoV-2 infection in schools, Austria, March 2021. Euro Surveill. 2021;26(34).

73. Wong SC, Chan VW, Yuen LL, AuYeung CH, Leung JO, Li CK, et al. Infection of healthcare workers despite a high vaccination rate during the fifth wave of COVID-19 due to Omicron variant in Hong Kong. Infect Prev Pract. 2023;5(1):100261.

74. Wu S, Archuleta S, Lim SM, Somani J, Quek SC, Fisher D. Serial antigen rapid testing in staff of a large acute hospital. Lancet Infect Dis. 2022;22(1):14-5.

75. Yun G, Park Y-J, Jang JE, Lee S, Kim KR, Jeong H, et al. COVID-19 Rapid Antigen Test Results in Preschool and School (March 2 to May 1, 2022). Pediatric Infection & Vaccine. 2024;31(1):113-21.

76. Davies M, Hill J, Goggins L, Peirce N, Smith J, Boulter M, et al. Daily SARS-CoV-2 testing after travel-related close contact notifications during elite sporting events hosted in the UK: a longitudinal study. BMJ Open Respir Res. 2025;12(1).

77. Del Fiol G, Kuzmenko TV, Orleans B, Chipman JJ, Greene T, Meads R, et al. Population-Based Digital Health Interventions to Deliver at-Home COVID-19 Testing: SCALE-UP II Randomized Clinical Trial. J Med Internet Res. 2025;27:e74145.

78. Mwangoka GW, Ali AM, Mrisho M, Mkopi A, Mahende M, Msuya HM, et al. Acceptability, Feasibility, and Uptake of COVID-19 Antigen Rapid Diagnostic Self-Testing at the Community Level in Tanzania. Am J Trop Med Hyg. 2025;112(4_Suppl):26-36.

79. Nacov JA, Salmanton-Garcia J, Grimm S, Stewart FA, Cremer LM, Rochel LM, et al. Self-testing for 5 respiratory viruses in adult VACCELERATE volunteers in Germany-a pilot study on multi-pathogen rapid antigen testing to monitor community-acquired acute respiratory infections. Front Public Health. 2025;13:1638280.

80. Qasmieh SA, Ferdinands JM, Chung JR, Wiegand RE, Flannery B, Rane MS, et al. Magnitude of Potential Biases in COVID-19 Vaccine Effectiveness Studies due to Differential Healthcare seeking following Home Testing: Implications for Test Negative Design Studies. medRxiv [preprint]. 2024.

81. Stirrup O, Blackstone J, Cullen-Stephenson I, Fenner R, Adams N, Leiser R, et al. VIVALDI-CT shaping care home COVID-19 testing policy: A pragmatic cluster randomised controlled trial of asymptomatic testing compared to standard care in care home staff. PLoS One. 2025;20(7):e0324908.

82. van Hagen CCE, Vos ERA, Delaunay CL, de Melker HE, Kissling E, Knol MJ. The effect of SARS-CoV-2 testing on healthcare seeking behaviour at primary care level: implications for COVID-19 vaccine effectiveness estimates in test-negative design studies. medRxiv [preprint]. 2025.

83. Qasmieh SA, Robertson MM, Teasdale CA, Kulkarni SG, Jones HE, Larsen DA, et al. The prevalence of SARS-CoV-2 infection and other public health outcomes during the BA.2/BA.2.12.1 surge, New York City, April-May 2022. Commun Med (Lond). 2023;3(1):92.

84. Qasmieh SA, Robertson MM, Teasdale CA, Kulkarni SG, Jones HE, McNairy M, et al. The prevalence of SARS-CoV-2 infection and long COVID in U.S. adults during the BA.4/BA.5 surge, June-July 2022. Prev Med. 2023;169:107461.

85. Lau J, Ioannidis JP, Terrin N, Schmid CH, Olkin I. The case of the misleading funnel plot. BMJ. 2006;333(7568):597-600.
